# A conserved complex of microneme proteins mediates rhoptry discharge in *Toxoplasma*

**DOI:** 10.15252/embj.2022113155

**Published:** 2023-10-27

**Authors:** Dylan Valleau, Saima M Sidik, Luiz C Godoy, Yamilex Acevedo‐Sánchez, Charisse Flerida A Pasaje, My‐Hang Huynh, Vern B Carruthers, Jacquin C Niles, Sebastian Lourido

**Affiliations:** ^1^ Whitehead Institute Cambridge MA USA; ^2^ Department of Biological Engineering Massachusetts Institute of Technology Cambridge MA USA; ^3^ Biology Department Massachusetts Institute of Technology Cambridge MA USA; ^4^ Department of Microbiology and Immunology University of Michigan Medical School Ann Arbor MI USA

**Keywords:** apicomplexan, CLAMP, invasion, rhoptry, secretion, *Toxoplasma*, Microbiology, Virology & Host Pathogen Interaction, Organelles

## Abstract

Apicomplexan parasites discharge specialized organelles called rhoptries upon host cell contact to mediate invasion. The events that drive rhoptry discharge are poorly understood, yet essential to sustain the apicomplexan parasitic life cycle. Rhoptry discharge appears to depend on proteins secreted from another set of organelles called micronemes, which vary in function from allowing host cell binding to facilitation of gliding motility. Here we examine the function of the microneme protein CLAMP, which we previously found to be necessary for *Toxoplasma gondii* host cell invasion, and demonstrate its essential role in rhoptry discharge. CLAMP forms a distinct complex with two other microneme proteins, the invasion‐associated SPATR, and a previously uncharacterized protein we name CLAMP‐linked invasion protein (CLIP). CLAMP deficiency does not impact parasite adhesion or microneme protein secretion; however, knockdown of any member of the CLAMP complex affects rhoptry discharge. Phylogenetic analysis suggests orthologs of the essential complex components, CLAMP and CLIP, are ubiquitous across apicomplexans. SPATR appears to act as an accessory factor in *Toxoplasma*, but despite incomplete conservation is also essential for invasion during *Plasmodium falciparum* blood stages. Together, our results reveal a new protein complex that mediates rhoptry discharge following host‐cell contact.

## Graphical abstract

Three apicomplexan‐specific microneme proteins are shown to form a complex essential for rhoptry discharge and invasion.

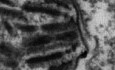

## Introduction

Apicomplexans are obligate unicellular parasites responsible for widespread diseases like malaria (*Plasmodium* spp.), cryptosporidiosis (*Cryptosporidium* spp.), and toxoplasmosis (*Toxoplasma gondii*). During the acute pathogenic stages of infection, apicomplexan parasites like *T. gondii* and *Plasmodium* spp. damage host cells through successive rounds of invasion, replication, and lysis (Blader *et al*, 2015; Bisio & Soldati‐Favre, 2019). Active invasion of host cells by apicomplexans is mediated by specialized organelles—micronemes and rhoptries—that secrete proteins from the apical end of parasites in a highly regulated manner (Carruthers & Sibley, 1997; Bisio & Soldati‐Favre, 2019). For a detailed review of apicomplexan invasion machinery, see (Cova *et al*, 2022). Secretion of the numerous small and oblong micronemes releases proteins throughout the extracellular stages of the parasite's lytic cycle, from egress to invasion. By contrast, an arsenal of effector proteins is directly injected into host cells from the larger club‐shaped rhoptries, in a process dependent on the prior secretion of micronemes (Carruthers & Sibley, 1997; Kessler *et al*, 2008; Cowman *et al*, 2017). Rhoptry proteins locally reshape the host cell membrane in preparation for invasion and contribute to rewiring host cell signaling. Although essential for apicomplexan invasion, the regulatory cascade and protein–protein interactions that mediate host cell detection and engage rhoptry secretion remain poorly understood.

Microneme proteins contribute to gliding motility, which supports displacement of parasites in tissues and invasion of host cells. Invasion additionally requires rhoptry discharge. Ca^2+^ signaling stimulates microneme fusion with the plasma membrane at the apical end of parasites to release proteins that perforate the host cell membrane, interact with the extracellular milieu, and bind host cell proteins (Carruthers & Sibley, 1999; Bisio & Soldati‐Favre, 2019; Dubois & Soldati‐Favre, 2019). Many microneme proteins remain tethered to the parasite's plasma membrane following secretion and act as adhesins that allow parasites to bind host cells (Tomley & Soldati, 2001; Cowman *et al*, 2017; Frénal *et al*, 2017). Some micronemal adhesins enable gliding motility by coupling the parasite's actomyosin system to the host cell surface or extracellular matrix. Adhesion complexes are translocated rearward along the parasite surface to produce forward motility (Frénal *et al*, 2017). Some microneme proteins have dedicated roles during invasion; the *T. gondii* microneme protein MIC8 and the *P. falciparum* proteins EBA175, Ripr, and CyRPA are species‐specific microneme proteins required for rhoptry effector secretion, but not parasite motility (Kessler *et al*, 2008; Singh *et al*, 2010; Volz *et al*, 2016; Knuepfer *et al*, 2019). Other proteins, like the microneme‐associate CRMP complex are required for rhoptry secretion and found across apicomplexans and even in free‐living ciliate secretion systems (Sparvoli *et al*, 2022; Singer *et al*, 2023). After secretion, some rhoptry effectors rewire host cell signaling to suppress innate immune responses, while other effectors, notably the components of the rhoptry neck (RON) complex, prepare the site of rhoptry secretion for apicomplexan invasion. A portion of RON2 becomes surface‐exposed following translocation into the host cell, where it tightly binds the microneme protein AMA1. This AMA1‐RON2 complex mediates the tight apposition between host and parasite membranes at the site of invasion (the moving junction) through which the parasite actively invades host cells using gliding‐like motility (Lamarque *et al*, 2011, 2014; Tyler & Boothroyd, 2011; Cowman *et al*, 2017; Frénal *et al*, 2017). The precise combination of surface‐exposed microneme proteins and host cell molecules that triggers rhoptry discharge is not known.

Recent cryo electron tomography (cryo‐ET) studies have begun to reveal the structure of the rhoptry secretion apparatus (RSA). The machinery involved in rhoptry discharge is more elaborate than the simple fusion of micronemes with the plasma membrane. Rhoptries docked to an apical vesicle that in turn binds the proteinaceous RSA centrally embedded in the apical plasma membrane (Aquilini *et al*, 2021; Mageswaran *et al*, 2021; Martinez *et al*, 2022). Correct assembly of the RSA complex is required for rhoptry discharge, as depletion of the protein Nd9 disrupts the RSA and blocks rhoptry protein secretion in *T. gondii* (Aquilini *et al*, 2021). Structural features of the RSA suggest that rhoptry proteins may be secreted through a channel at its center. Ca^2+^ signaling is also thought to contribute to rhoptry discharge. TgFER2, a conoid‐associated Ca^2+^‐binding protein, localizes to the cytosolic surface of the rhoptries and is essential for their discharge (Coleman *et al*, 2018). Moreover, treatment of parasites with Ca^2+^ ionophores leads to apparent fusion between the apical vesicle and the docked rhoptries (Segev‐Zarko *et al*, 2022). The common reliance of both micronemes and rhoptries on Ca^2+^ signaling complicates deconvolution of the series of events that mediate invasion.

Proteins associated with rhoptry discharge appear conserved to different extents across the phylum. The RSA appears to be related to the secretory organelles of free‐living ciliates, which are members of the superphylum Alveolata along with apicomplexans (Aquilini *et al*, 2021). Apicomplexans, therefore, appear to have adapted this conserved secretion machinery to their parasitic lifestyles. The CRMP proteins—required for rhoptry discharge but not the assembly of the apical vesicle or RSA—are broadly conserved among Alveolata much like the Nd proteins (Sparvoli *et al*, 2022) and bind additional microneme proteins in *T. gondii* (Sparvoli *et al*, 2022; Singer *et al*, 2023). MIC8, a microneme protein required for rhoptry discharge in *T. gondii*, is limited to coccidians (Kessler *et al*, 2008) suggesting apicomplexans have clade‐specific determinants of rhoptry discharge that potentially tune the process to their respective niches.

We previously demonstrated that the microneme protein CLAMP is necessary for *T. gondii* invasion of host cells (Sidik *et al*, 2016b). CLAMP is conserved across Apicomplexa, and its homolog is necessary for *P. falciparum* to progress through its lytic cycle, implying a conserved function. Here, we identify two additional microneme proteins that interact with CLAMP and uncover the essential function of this complex in rhoptry discharge. Our results strengthen the link between micronemes and rhoptries during invasion, adding to the understanding of the mechanisms that apicomplexan parasites use to establish their intracellular replicative niche.

## Results

### CLAMP is a transmembrane microneme protein with a cytosolic C terminus

**Figure 1:**
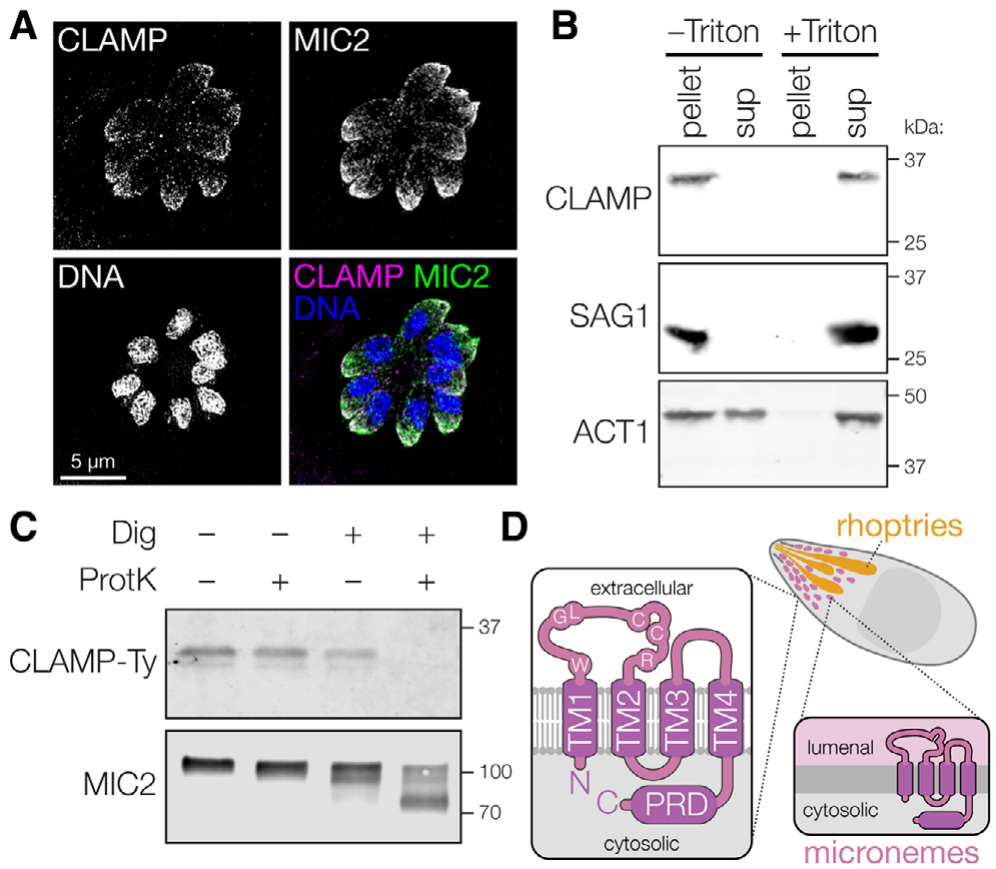
CLAMP is a microneme membrane protein with a cytosolic C terminus Immunofluorescence microscopy revealed that CLAMP‐Ty colocalizes with the microneme protein MIC2.Parasites expressing CLAMP‐Ty were disrupted by freeze‐thawing in the presence or absence of detergent (Triton X‐100), then separated into pellet and supernatant (sup) fractions by ultracentrifugation. Anti‐Ty immunoblotting revealed that CLAMP‐Ty fractionates with the membrane protein SAG1, and differentially from cytosolic actin (ACT1).Sensitivity of CLAMP‐Ty and MIC2 to Proteinase K digestion in intact parasites or those treated with digitonin to selectively disrupt their plasma membrane. Anti‐Ty immunoblotting revealed that CLAMP's cytosolic Ty tag was degraded in semi‐permeabilized parasites while the luminal portion of MIC2 remained intact.Model of the topology for lumenal CLAMP and on the plasma membrane following microneme exocytosis. Residues shared with claudins are highlighted. Source data are available online for this figure.

As previously reported, CLAMP is a transmembrane protein that colocalizes with the microneme marker MIC2 (Fig 1A) (Sidik *et al*, 2016b). Several transmembrane microneme proteins have been investigated (Carruthers & Tomley, 2008; Dubois & Soldati‐Favre, 2019), but none with the predicted topology of CLAMP. To examine the association of CLAMP with membranes, we prepared lysates from parasites in which the endogenous CLAMP locus was C‐terminally tagged with the Ty epitope (CLAMP‐Ty) (Bastin *et al*, 1996), and fractionated them by ultracentrifugation in the presence or absence of the detergent Triton X‐100. Consistent with its membrane association, CLAMP co‐fractionated with the GPI‐anchored protein SAG1, moving from the pellet to the supernatant in the presence of detergent (Fig 1B). By contrast, the cytosolic protein actin was largely found in the supernatant regardless of the presence of detergent.

Topology modeling (Dobson *et al*, 2015) predicts that CLAMP has four transmembrane helices (TMs), with its N and C termini extending into the cytosol. To confirm that CLAMP's C terminus is cytosolic we used a Proteinase K protection assay in which the plasma membrane is gently permeabilized with digitonin to keep internal membranes intact and inaccessible to protease digestion. The single‐pass transmembrane microneme protein MIC2 was used to calibrate the assay, based on availability of a monoclonal antibody against its lumenal domain and exposure of a short segment of its C terminus to the cytosol. Under conditions that preserve microneme integrity, the apparent molecular weight of MIC2 decreased when the samples were exposed to proteinase K, consistent with exclusive degradation of its cytosolic portion (Fig 1C). Similarly, CLAMP's cytosolic C‐terminal Ty tag was degraded under conditions that preserved the lumenal MIC2 signal, in support of the predicted topology (Fig 1D).

### CLAMP‐deficient parasites establish normal contact with host cells prior to invasion

**Figure EV1:**
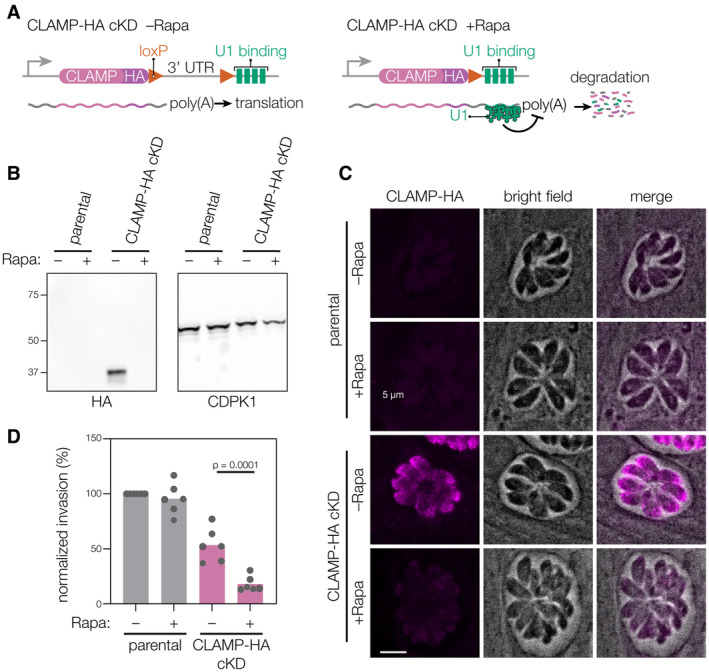
Generation of a non‐fluorescent CLAMP conditional knockdown in *T. gondii* strain ADiagram of the CLAMP‐HA cKD scheme. Induction of DiCre dimerization by rapamycin (Rapa) treatment leads to excision of the 3′UTR between the stop codon and an array of U1 binding sites, leading to U1‐mediated degradation of the mRNA transcript.B, CKnockdown of CLAMP‐HA expression was assessed following a 2 h treatment with rapamycin. Samples were analyzed 48 h after the treatment by anti‐HA immunoblotting (B), or after 24 h after the treatment by anti‐HA immunofluorescence (C).DCLAMP‐HA knockdown leads to inhibition of host cell invasion for parasites allowed to invade for 20 min. Normalized percent invasion refers to the number of intracellular parasites/host cell nuclei, which is then normalized to the vehicle‐treated parental condition. *P* values derive one‐way ANOVA followed by Sidak's multiple comparison test for *n* = 6 biological replicates. Source data are available online for this figure.

Our previous work showed that CLAMP‐deficient parasites fail to invade host cells (Sidik *et al*, 2016b). Invasion is a multi‐step process involving host cell attachment, secretion of microneme and rhoptry contents, and formation of the moving junction. To pinpoint the precise step of invasion affected by CLAMP knockdown, we generated a CLAMP conditional knockdown strain (CLAMP‐HA cKD) using the U1 system. In this strain, a brief rapamycin pulse induces recombination of loxP sites flanking the 3′ UTR of the tagged gene and places an array of U1‐binding sequences in line with the transcript, interfering with mRNA expression (Pieperhoff *et al*, 2015). We confirmed that CLAMP‐HA cKD loses CLAMP expression when treated with rapamycin, as observed by immunoblot and immunofluorescence (Fig EV1A–C).

**Figure 2:**
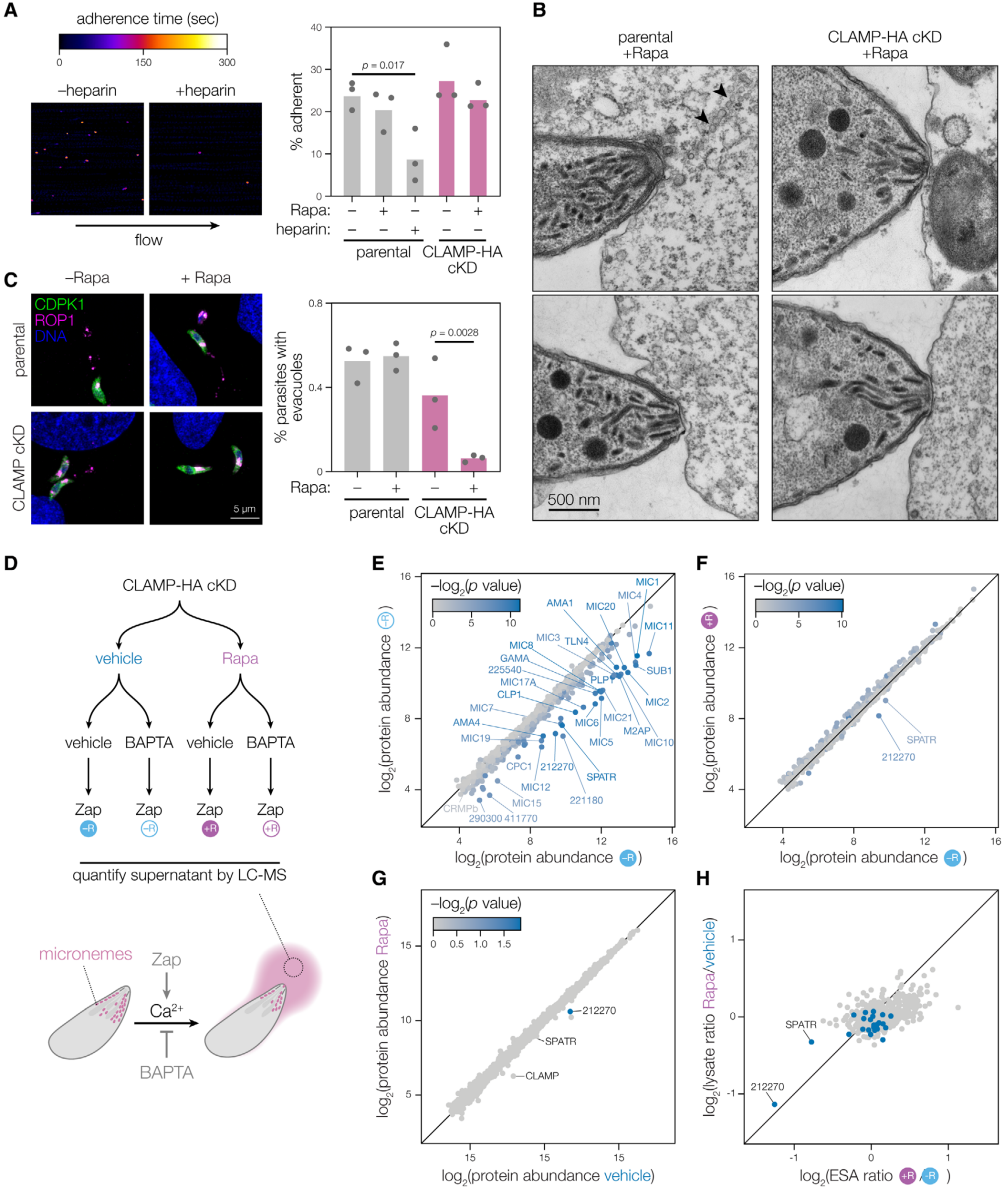
CLAMP loss specifically affects rhoptry discharge Mycalolide B–treated parasites were imaged as they interacted with host cells under flow conditions. The percent of parasites remaining adhered to host cells for over 3 s was quantified. Pre‐treating host cells with heparin reduced adhesion, but CLAMP‐deficient parasites did not differ significantly from control strains. Mean is plotted for *n* = 3 biological replicates; *P* value for one‐way ANOVA with Sidak's multiple comparison test.Electron microscopy of mycalolide B–treated parasites interacting with K562 cells. Sections of the apical complexes of CLAMP cKD and parental strains treated with rapamycin (+Rapa) are shown. Black arrows indicate the position of structures reminiscent of evacuoles.Evacuole quantification of CLAMP cKD and parental parasites pre‐treated with rapamycin (+Rapa) or vehicle (–Rapa). CLAMP depletion resulted in decreased association with evacuoles. Isolated parasites were pre‐treated with cytochalasin D to prevent invasion before adding them to host cell monolayers. Evacuoles and parasites were visualized staining for ROP1 and CDPK1, respectively. Mean is plotted for *n* = 3 biological replicates; *P* value for one‐way ANOVA with Sidak's multiple comparisons test.Experimental setup for the quantitative mass spectrometric comparison of excretory‐secretory antigens (MS‐ESA). CLAMP cKD parasites were treated with zaprinast following pre‐treatment with the calcium chelator BAPTA‐AM or a vehicle control.Comparison of protein abundances in MS‐ESA from the –Rapa samples treated with either vehicle (*x* axis) or BAPTA‐AM (y axis) demonstrates detection of known secreted microneme proteins. Color scale indicates corrected *P* value for the comparison of the two axes.Comparison of MS‐ESA abundance from induced, zaprinast‐treated samples comparing the CLAMP cKD following pre‐treatment with vehicle (*x* axis; as in E) or rapamycin (*y* axis).Quantifying the abundance of proteins in the total lysate of parasites in either the –Rapa or +Rapa conditions used for the MS‐ESA. CLAMP depletion affects the abundance of only certain proteins.Comparison of the relative ratios of secretion to the overall abundance when CLAMP is present or knocked down shows that TGGT1_212270 abundance decreases in both the ESA fraction and the parasite lysate when CLAMP is depleted, while SPATR secretion is more affected than its overall protein abundance. The microneme proteins annotated in (E) are displayed in blue. Source data are available online for this figure.

Many microneme proteins function as adhesins that facilitate attachment to host cells. We therefore quantified the ability of CLAMP‐deficient parasites to adhere to human fibroblasts under flow conditions. Parasites were perfused over fibroblasts grown in microfluidic chambers, and we counted those that stably adhered to host cells for at least 3 s (Fig 2A, Movie EV1). In agreement with prior studies, pre‐treating host cells with heparin inhibited parasite attachment (Monteiro *et al*, 1998). By contrast, CLAMP knockdown had no effect on *T. gondii* adhesion to host cells (Movie EV2).

**Figure EV2:**
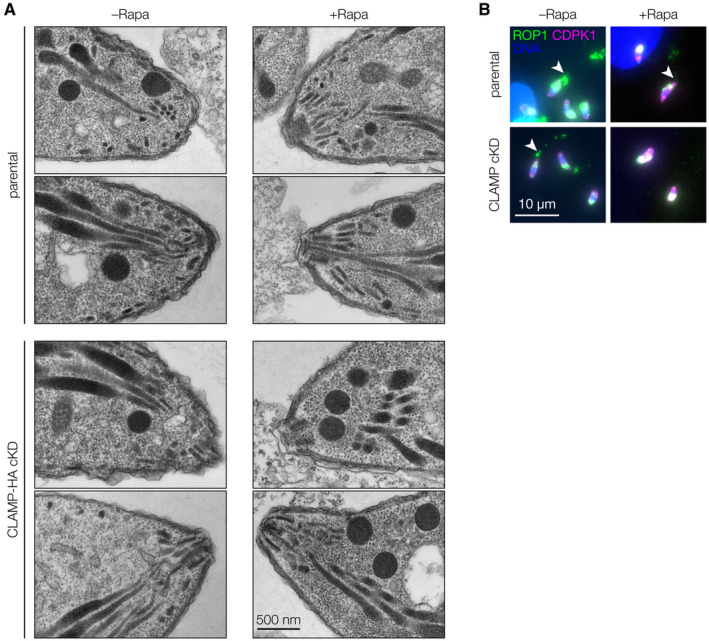
CLAMP knockdown does not alter apical‐complex morphology in *T. gondii* Additional images of CLAMP‐HA cKD and parental parasites prepared for electron microscopy 48 h after a 2 h treatment with 50 nM rapamycin (+Rapa) or a DMSO vehicle control (–Rapa).Representative images of evacuoles collected with an epifluorescence microscope and used for evacuole quantification. Arrowheads indicate examples of evacuole formation used in quantification.

Following adhesion, parasites reorient to make apical contact with the host cell plasma membrane. To examine the ultrastructure of the parasite apical end during this stage of the invasion process, we performed transmission electron microscopy on the CLAMP cKD and its parental strain as they interacted with K562 human lymphoblast cells. The non‐adherent nature of K562 cells facilitated preparations for electron microscopy. Parasites were pre‐treated with mycalolide B to prevent actin‐based invasion without affecting host cell adhesion (Cirelli *et al*, 2014). No morphological differences could be observed between the apical ends of the CLAMP‐HA cKD and its parental strain, irrespective of rapamycin treatment (Fig EV2A). In both cases, examples of tightly apposed parasite apical ends and regions of the host plasma membrane could be observed, regardless of CLAMP knockdown (Fig 2B). In rare cases, apical ends from the parental strain were associated with trails of small vacuoles, reminiscent of the previously described evacuoles that result from rhoptry discharge (Håkansson *et al*, 2001). Similar structures were not observed in proximity to CLAMP‐knockdown parasites. Nevertheless, these results demonstrate that, in the absence of CLAMP, parasites retain the ability to correctly reorient and establish apical contact with host cells, despite failing to initiate invasion.

### CLAMP is required for rhoptry discharge and secretion of two other microneme proteins

Following microneme‐dependent apical attachment to host cells, parasites discharge rhoptries. Rhoptry discharge is essential, as the secreted proteins include critical components of the tight junction required for invasion of host cells (Carruthers & Sibley, 1997; Ben Chaabene *et al*, 2021). Inhibition of parasite gliding motility (e.g., preventing actin polymerization) blocks invasion but does not affect rhoptry discharge, which can be observed by examining the localization of rhoptry proteins to evacuoles (Håkansson *et al*, 2001; Kessler *et al*, 2008; Sibley, 2010). To determine whether CLAMP‐deficient parasites secrete evacuoles, we quantified ROP1‐containing evacuole formation (Fig 2C). CLAMP‐depleted parasites and controls were pre‐treated with the actin polymerization inhibitor cytochalasin D and allowed to interact with host cells for a few minutes prior to fixation. Staining for the rhoptry protein ROP1 revealed that 30–50% of control parasites were associated with evacuoles (Figs 2C and EV2B, arrows). By contrast, evacuoles were only associated with 6% of CLAMP‐deficient parasites—low enough to be attributable to the small proportion of CLAMP‐HA cKD parasites that fail to undergo recombination. We conclude that loss of CLAMP results in a failure to discharge rhoptries, preventing invasion.

Only two groups of surface‐exposed proteins, MIC8 and the CRMPs, have been specifically associated with rhoptry discharge in *T. gondii* (Kessler *et al*, 2008; Sparvoli *et al*, 2022; Singer *et al*, 2023). We previously showed that CLAMP depletion does not affect secretion of the microneme protein MIC2 (Sidik *et al*, 2016b). Nevertheless, the similarity between the CLAMP phenotype and that of the CRMPs and MIC8 motivated a systematic evaluation of CLAMP's role in microneme secretion. Since micronemes are secreted in a Ca^2+^‐dependent manner, we developed a quantitative mass spectrometry–based method to measure the Ca^2+^‐dependent excretory‐secretory antigen secretome of *T. gondii* strains (MS‐ESA; Fig 2D). Zaprinast is a phosphodiesterase inhibitor that triggers Ca^2+^ release from intracellular stores by activating the cGMP‐dependent protein kinase (Yuasa *et al*, 2005; Lourido *et al*, 2012; Brown *et al*, 2016; Sidik *et al*, 2016a). We first compared the ESA fractions from zaprinast‐stimulated parasites that had been pre‐treated with either the Ca^2+^‐chelator BAPTA‐AM or a vehicle control (Carruthers & Sibley, 1999). As anticipated, microneme proteins were more abundant in the ESA from the vehicle control compared to the BAPTA‐AM–treated parasites (Fig 2E). Twenty‐seven of the proteins significantly enriched from parasites with an intact Ca^2+^‐signaling pathway were either annotated as microneme proteins or found to be microneme‐localized by spatial proteomics (Barylyuk *et al*, 2020) (Fig 2E).

Our MS‐ESA results highlight several potential microneme proteins that have been poorly characterized or only recently identified. Among the Ca^2+^‐dependent secreted proteins we found MIC19—which was not predicted to be microneme localized by spatial proteomics—as well as the recently identified microneme protein MIC21 (Barylyuk *et al*, 2020; Tagoe *et al*, 2021). We also found CRMPb, which inconsistently localizes with micronemes and forms a complex with CRMPa and MIC15 (Sparvoli *et al*, 2022; Singer *et al*, 2023). Several additional proteins secreted in a Ca^2+^‐dependent manner have not been examined in detail. TGGT1_212270 and TGGT1_221180, were annotated as microneme‐localized by spatial proteomics, but have not been studied further (Barylyuk *et al*, 2020). TGGT1_411770 has not been localized by other datasets, but its paralog TGGT1_319090 is predicted to be microneme‐localized. It is possible that some of the proteins secreted in a Ca^2+^‐dependent manner do not originate from micronemes. TGGT1_225540 and TGGT1_290300 were also secreted in a Ca^2+^‐dependent manner yet were not microneme‐localized by spatial proteomics. Nevertheless, our MS‐ESA method is a useful tool to characterize the Ca^2+^‐dependent secretome and measure its alteration by chemical or genetic perturbations.

Using MS‐ESA, we studied the effect of CLAMP knockdown on microneme secretion. Nearly all microneme proteins were secreted to a similar extent in the presence or absence of CLAMP (Fig 2F). Notably, MIC8 and CRMP complex components were unaffected by CLAMP repression. However, two proteins showed a striking CLAMP dependency: SPATR and one of the previously unstudied proteins, TGGT1_212270. We examined whether changes in SPATR and TGGT1_212270 abundance in the ESA samples were due to a secretion defect or protein destabilization. Measuring the relative abundance of proteins in the lysates of rapamycin or vehicle‐treated CLAMP‐HA cKD by quantitative mass spectrometry, the only significantly depleted protein was TGGT1_212270 (Fig 2G). Comparing the abundance ratios of proteins in the ESA fraction and whole‐parasite lysates suggests that CLAMP knockdown leads to decreased TGGT1_212270 stability, whereas the reduction of SPATR in the ESA fraction is likely due to a secretion or trafficking defect (Fig 2H). Prior studies suggest that SPATR and TGGT1_212270 localize to micronemes where CLAMP could indeed influence their trafficking and stability (Huynh *et al*, 2014; Sidik *et al*, 2016b; Barylyuk *et al*, 2020).

### CLAMP, CLIP, and SPATR comprise a stable microneme complex

**Figure 3:**
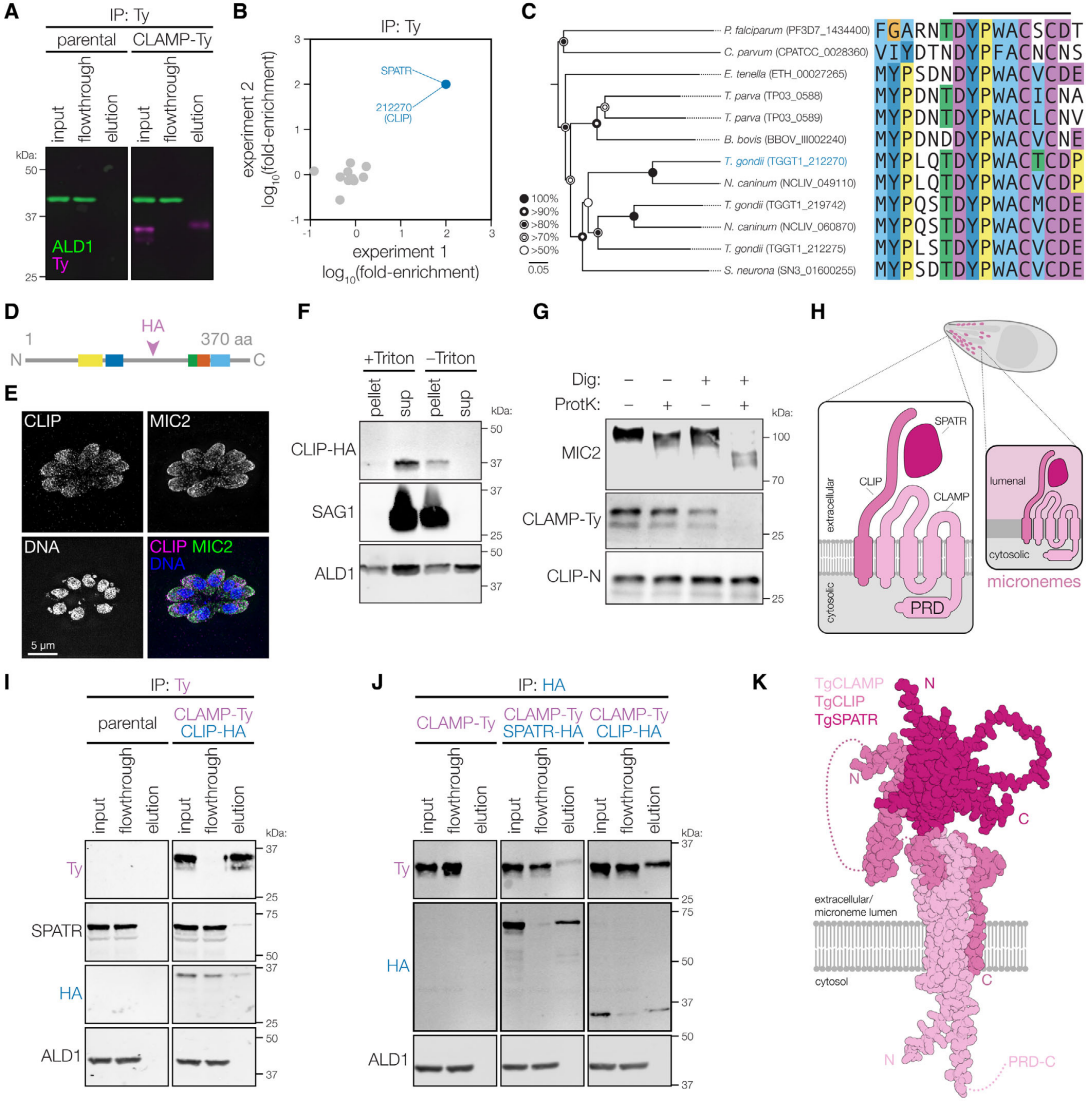
CLAMP forms a complex with CLIP (TGGT1_212270) and SPATR in micronemes AImmunoblot of samples from an immunoprecipitation of CLAMP‐Ty comparing the tagged strain to the parental control. ALD1 is used as a soluble antigen to monitor the specificity of the enrichment.BSPATR and TGGT1_212270 (CLIP) were reproducibly co‐immunoprecipitated with CLAMP‐Ty compared to the untagged control. Label‐free quantification of eluates by mass spectrometry was used to calculate fold‐enrichment.CNeighbor‐joining phylogenetic tree of selected CLIP homologs from across the apicomplexan phylum. The region around conserved motif 1 is shown within the sequence alignment. Bootstrap values for 1,000 trials are displayed. Abbreviated species names are provided for *Toxoplasma gondii*, *Plasmodium falciparum*, *Theileria parva*, *Babesia bovis*, *Neospora caninum*, *Sarcocystis neurona*, *Hammondia hammondi*, *Eimeria tenella*, and *Cryptosporidium parvum*. An extended tree and additional conserved motifs are provided in Fig EV3A.DDiagram of CLIP's internal HA tagging site (after Arg210) between conserved motifs.EImmunofluorescence staining with anti‐HA reveals that CLIP‐HA colocalizes with the microneme marker MIC2.FCLAMP‐Ty/CLIP‐HA parasites were disrupted using multiple freeze/thaw cycles, then fractionated by ultracentrifugation in the presence or absence of detergent (Triton X‐100). CLIP partitions similarly to the membrane protein SAG1 and differently from the cytosolic protein Aldolase.GCLIP is insensitive to Proteinase K treatment, while the cytosolic Ty tag of CLAMP‐Ty and cytosolic portion of MIC2 are degraded by Proteinase K in parasites with digitonin‐permeabilized plasma membranes.HModel of CLAMP, CLIP, and SPATR topology at the microneme membrane or plasma membrane, following secretion.I, JImmunoblot of samples from an immunoprecipitation of CLAMP‐Ty (I) or the HA‐tagged interacting partners (J), confirming the stable interaction of all three proteins.KA structural model of the CLAMP complex, predicted using Alphafold2. The unstructured loop regions of all three proteins have been truncated for clarity, including the C‐terminal proline‐rich domain of CLAMP and the internal unstructured region of CLIP (dotted lines). Source data are available online for this figure.

We used co‐immunoprecipitation to identify proteins that interact with CLAMP. Using parasites in which CLAMP was endogenously tagged with the Ty epitope (CLAMP‐Ty), we immunoprecipitated CLAMP‐Ty from detergent‐solubilized parasite lysates (Fig 3A), and analyzed captured proteins by mass spectrometry. Throughout our experiments, CLAMP has been inconsistently detected by MS, despite being clearly observed in the samples by immunoblot. We attribute the absence of CLAMP from our MS results to its hydrophobicity and unusual pattern of tryptic fragmentation. Nevertheless, comparing the fold enrichment in protein abundance between immunoprecipitations from CLAMP‐Ty parasites over their parental counterparts revealed two proteins enriched in both biological replicates: SPATR and TGGT1_212270 (Fig 3B)—notably, the two CLAMP‐dependent constituents of the ESA (Fig 2F).

**Figure EV3:**
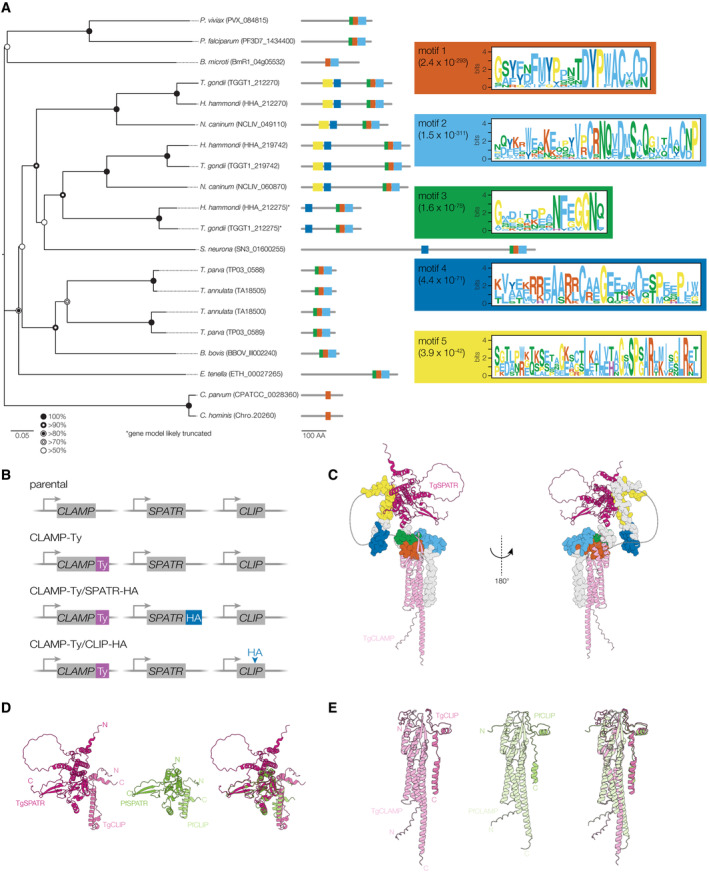
Extended analysis of CLIP homologs ANeighbor‐joining phylogenetic tree of CLIP homologs. Bootstrap values for 1,000 trials are displayed. The logo plots for the top five motifs identified using MEME analysis are shown in boxes colored to match the domains on the protein models. E‐value for each motif within the set of 20 CLIP homologs is indicated in parentheses. Sequence analysis suggests some of the models are incomplete (*), and a *N. caninum* sequence syntenic with TGGT1_212275 could be detected, but is not annotated in the current version of the genome (ToxoDB release 60). Abbreviated species names are provided for *Toxoplasma gondii*, *Plasmodium vivax*, *Plasmodium falciparum*,*Theileria parva*, *Theileria annulata*, *Babesia bovis*, *Babesia microti*, *Neospora caninum*, *Sarcocystis neurona*, *Hammondia hammondi*, *Eimeria tenella*, and *Cryptosporidium parvum*.BDiagram of strains carrying HA tags on CLIP and/or SPATR used for reciprocal immunoprecipitations SPATR was tagged at its C terminus and CLIP internally, following Arg210.CThe Alphafold model of the *T. gondii* CLAMP complex showing the conserved CLIP motifs identified in EV3A. Secondary structure cartoons are displayed for CLAMP and SPATR, while for CLIP the motifs are highlighted in a space‐filling model. Two views are displayed with the indicated rotation. Colors for CLIP correspond to the assigned motif colors in Fig EV3A.D, EThe *T. gondii* and *P. falciparum* Alphafold complex models for CLAMP, CLIP and SPATR share consistent interaction surfaces, with SPATR binding the N‐terminal portion of CLIP (D) and CLAMP binding the C‐terminal portion of CLIP (E). Data information: For C–E, the models have been trimmed of their large unstructured regions as in Fig 3K.

Based on the observed protein–protein interaction, we named TGGT1_212270 CLAMP‐Linked Invasion Protein (CLIP). Searches of apicomplexan sequences (veupathdb.org) using the basic local alignment search tool (BLAST) identified multiple CLIP‐related sequences broadly distributed across the apicomplexan phylum. Examining the sequences revealed highly conserved motifs near the C‐terminal TM of homologous sequences (DYP[WF]ACxC), which recovered 1–3 matching sequences from all apicomplexan genomes examined (Figs 3C and EV3A). Most apicomplexans have a single ortholog, including malaria‐causing parasites from the genus *Plasmodium*. However, gene‐duplication appears to have given rise to groups of paralogs, with *Theileria* spp. harboring two, and most cyst‐forming coccidians like *T. gondii* harboring three. The early branching coccidian *Sarcocystis neurona* appears to have a single CLIP ortholog. Both CLIP paralogs in *T. gondii* appear to be dispensable during the tachyzoite stages (Sidik *et al*, 2016b) and show peaks of expression during the chronic stages (TGGT1_212275) or in the definitive host (TGGT1_219742) (Hehl *et al*, 2015; Ramakrishnan *et al*, 2019; Waldman *et al*, 2020), suggesting they might function during other stages of the life cycle. MEME analysis (Bailey & Elkan, 1994; Bailey *et al*, 2009) of the CLIP‐related sequences revealed additional regions of high conservation (Fig EV3A), despite overall low sequence similarity. We conclude that CLIP may have as broad a pattern of conservation as previously proposed for CLAMP (Sidik *et al*, 2016b), but appears to be under stronger positive selection.

We sought to confirm the subcellular localization of CLIP. However, attempts to modify the *CLIP* locus for N‐ or C‐terminal tagging proved unsuccessful, likely due to the presence of N‐terminal secretion signals and the high degree of conservation along the C terminus (Fig EV3A). We successfully targeted a less conserved internal region, after Arg210 of the coding sequence, by marker‐free integration of an HA epitope (Fig 3D). Using super‐resolution microscopy on the strain in which CLIP was HA tagged, we found that, like CLAMP (Fig 1A) and SPATR (Huynh *et al*, 2014), CLIP adopts a punctate apical pattern that largely colocalizes with the microneme marker MIC2 (Fig 3E). Differential ultracentrifugation of parasite lysates in the presence or absence of detergent confirmed the membrane association of CLIP, based on its co‐fractionation with the known membrane protein SAG1 (Fig 3F). This is consistent with an *in silico* topology analysis (Dobson *et al*, 2015) predicting that CLIP has a single transmembrane helix at its C terminus. To confirm the topology of CLIP in the microneme membrane, we performed a proteinase K protection assay, detecting CLIP using a polyclonal antibody we generated against its N terminus (anti‐CLIP‐N). We found no degradation or molecular weight shift for CLIP, while the C‐terminally tagged Ty epitope on CLAMP‐Ty and the cytosolic domain of MIC2 were degraded (Fig 3G), confirming CLIP separated from the cytosol. CLIP peptides are detected in the Ca^2+^‐dependent ESA fraction (Fig 2E), consistent with residency in the microneme lumen and secretion to the extracellular space. These results suggest a model where SPATR and the ectodomain of CLIP reside in the microneme lumen while CLAMP and the C‐terminal transmembrane helix of CLIP tether the complex to the microneme membrane and the parasite surface following microneme exocytosis (Fig 3H).

To validate the putative interactions between CLAMP, CLIP, and SPATR, we generated strains in which SPATR and CLIP were HA‐tagged in the CLAMP‐Ty background (Fig EV3B). We immunoprecipitated CLAMP‐Ty and used immunoblotting to confirm its interaction with both CLIP and SPATR (Fig 3I). We also performed reciprocal immunoprecipitations using the HA‐tags of the interacting partners and found that CLIP‐HA and SPATR‐HA both co‐immunoprecipitated CLAMP (Fig 3J). Further corroborating the formation of the complex, Alphafold Multimer (Mirdita *et al*, 2022) predicted a model consistent with the expected topology of the subunits and aligning CLIP's C‐terminal TM helix with CLAMP's 4 TMs. The model also predicts that the C terminus of CLIP interacts with CLAMP while its N terminal region contacts SPATR, with a flexible linker between the two regions (Fig 3K). This model also predicted structural contacts between the three highly conserved CLIP C‐terminal motifs (Fig EV3A) and CLAMP's structured lumenal region (Fig EV3C), supporting conservation of the binding interface. Independent prediction of the *Plasmodium falciparum* complex between *Pf*CLAMP, *Pf*CLIP, and *Pf*SPATR revealed a similar overall structure—notably, the CLAMP‐CLIP and the CLIP‐SPATR interfaces occupy the same grooves in the *T. gondii* and *P. falciparum* models (Fig EV3D and E). Taken together, these results provide strong evidence for a stable and conserved three‐way microneme complex between CLAMP, CLIP, and SPATR (Fig 3H).

### CLIP is essential for *T. gondii* invasion

**Figure 4:**
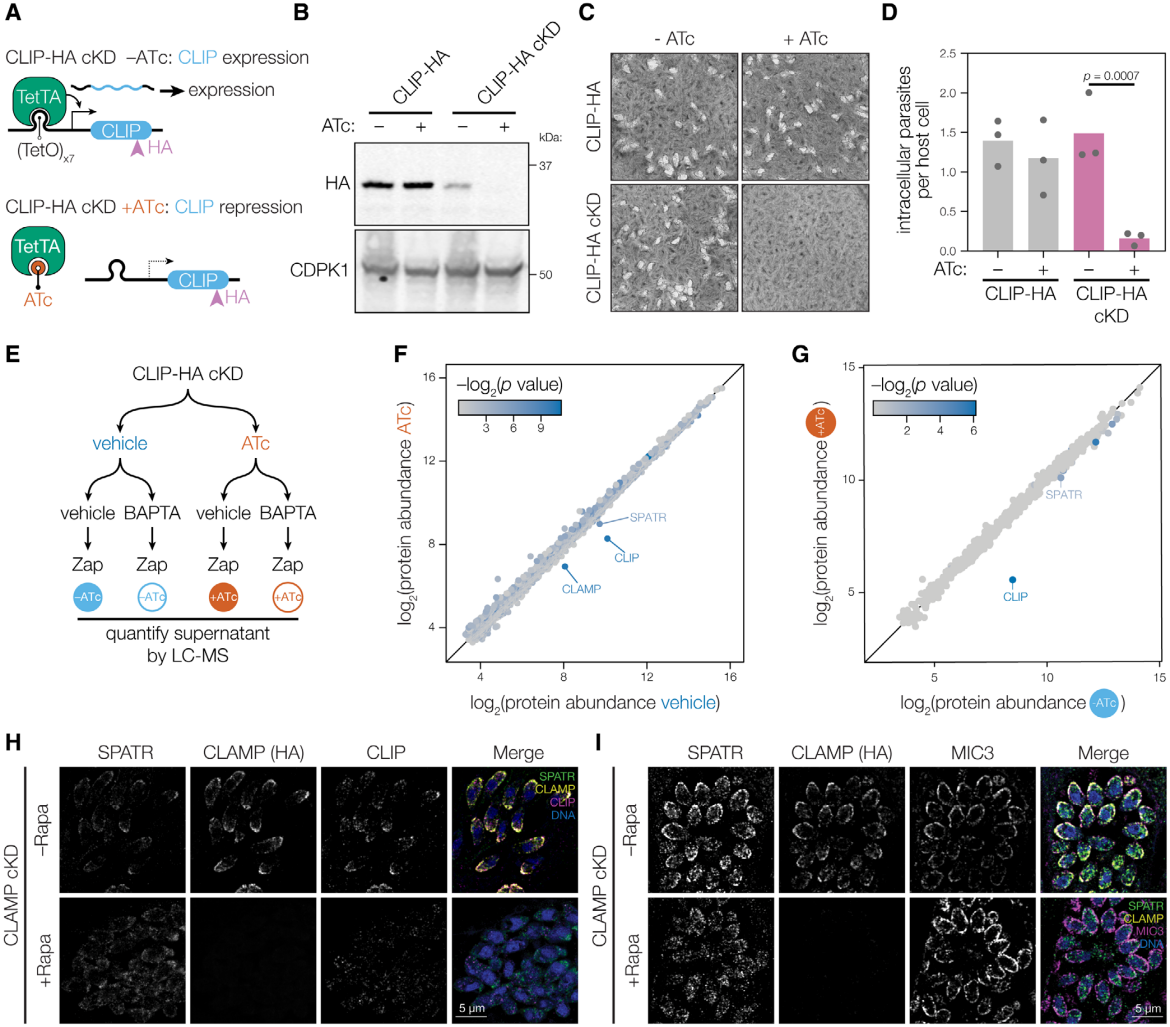
CLIP is a secreted single‐pass transmembrane microneme protein that is necessary for invasion AThe CLIP conditional knockdown strain (CLIP‐HA cKD) was made by replacing the region 5′ to the coding sequence of CLIP‐HA with the TetO_7_‐*SAG1* promoter in a Tet‐transactivator expressing background (TetTA). Addition of anhydrotetracycline (ATc) results in release of the TetTA and inhibition of transcription.BGrowth in the presence of ATc for 48 h leads to conditional knockdown of CLIP‐HA cKD as measured by anti‐HA immunoblot. CDPK1 is used as a loading control. Quantification and replicates of the knockdown blot can be found in Fig EV4C and D.CCLIP is essential to the lytic cycle. CLIP‐HA cKD parasites failed to form plaques when treated with ATc, while the CLIP‐HA parental strain formed plaques regardless of ATc treatment.DCLIP‐HA cKD parasites fail to invade host cells after 48 h of ATc treatment. Mean is plotted for *n* = 3 biological replicates; *P* values derive from a one‐way ANOVA followed by Sidak's multiple comparison test.EExperimental setup for the MS‐ESA and whole proteome analysis of CLIP conditional knockdown. CLIP‐HA cKD parasites that had CLIP knocked down with ATc or were treated with vehicle control for 48 h. For the MS‐ESA, parasites were then stimulated with zaprinast following pre‐treatment with the Ca^2+^ chelator BAPTA‐AM or a vehicle control.F, GDepletion of CLIP by 48 h ATc treatment (y axis) did not dramatically affect the stability of proteins other than CLIP in total parasite lysate compared to vehicle control (*x* axis; F). Comparison of the MS‐ESA for CLIP knockdown (+ATc, *y* axis) to vehicle control (−ATc, *x* axis) reveals that CLIP knockdown leads to depletion of CLIP, CLAMP, and SPATR in the ESA fraction (G). Color scale indicates corrected *P* value for the comparison of the two axes for F‐G.H, IDepletion of CLAMP leads to decreased CLIP abundance and disruption of SPATR localization (H and I). The microneme protein MIC3 is used for reference (I). Source data are available online for this figure.

**Figure EV4:**
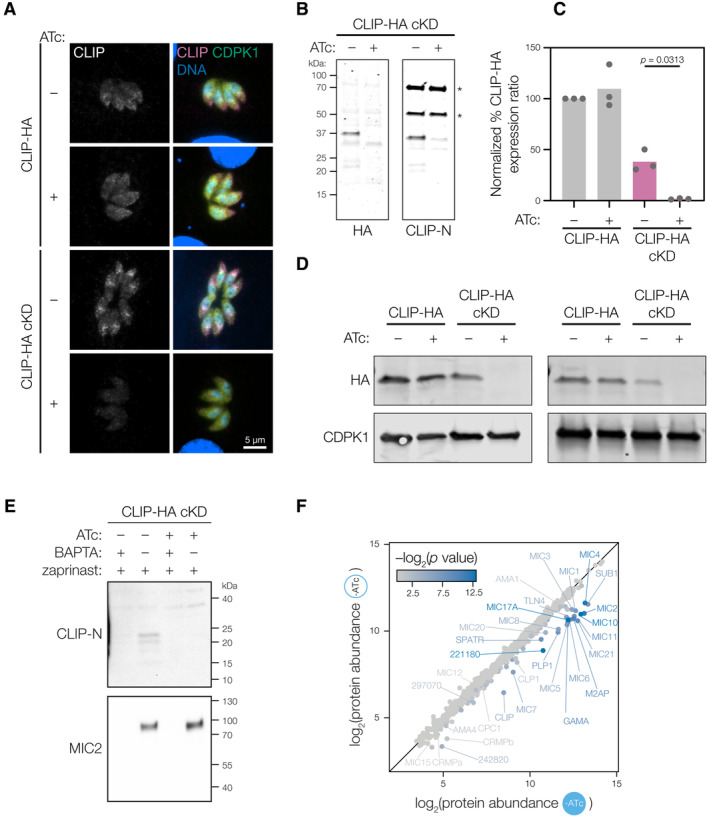
Conditional CLIP knockdown disrupts microneme localization and blocks detection of CLIP in the extracellular secreted antigen fraction ATreatment with anhydrotetracycline (ATc) leads to loss of CLIP‐HA signal in the CLIP‐HA cKD strain, as visualized by anti‐HA immunofluorescence.BFurther validation of CLIP knockdown with anti‐HA and the anti‐CLIP‐N antibody generated against the N‐terminal 187 residues of CLIP. Non‐specific bands detected by the anti‐CLIP‐N serum are marked with an asterisk (*).C, DAddition of the TetO7 operator upstream of the CLIP coding sequence leads to a reduction in basal expression of CLIP, while treatment of this strain with ATc completely blocks CLIP expression (C). Quantification of CLIP‐HA and CLIP‐HA cKD strains in the presence and absence of vehicle or ATc was done by western blot in triplicate, using the gel shown in Fig 4B as well as the 2 displayed in (D). *P* values in C derive from one‐way ANOVA followed by Sidak's multiple comparison test for *n =* 3 biological replicates.EProbing the CLIP‐HA cKD MS‐ESA samples with anti‐CLIP‐N confirms that a form of cleaved CLIP is found in the ESA fraction, as is the microneme protein MIC2. Knockdown of CLIP with ATc leads to loss of CLIP in the ESA fraction. As expected, microneme secretion is inhibited by BAPTA‐AM and induced by zaprinast treatment.FMicroneme proteins are generally found in the ESA fraction of the vehicle‐treated CLIP‐HA cKD strain as shown by treatment with BAPTA‐AM to inhibit secretion (*y* axis) or a vehicle control (*x* axis). Source data are available online for this figure.

Both CLAMP and SPATR mutants have invasion defects (Huynh *et al*, 2014; Sidik *et al*, 2016b), which, in the case of CLAMP, leads to a complete block in the parasite's lytic cycle. To determine whether CLIP knockdown phenocopies that of its binding partners, we constructed an inducible CLIP‐HA cKD mutant using the Tet‐off transactivator system (Meissner *et al*, 2002) (Fig 4A). We confirmed that growth of CLIP‐HA cKD in anhydrotetracycline (ATc) leads to loss of CLIP‐HA expression as observed by anti‐HA and anti‐CLIP‐N immunoblot and immunofluorescence assays (Figs 4B and EV4A and B). We noted that addition of the TetO7 sequence ahead of CLIP's CDS partially suppressed CLIP expression to approximately 40% of what was observed in the parental CLIP‐HA strain (Fig EV4C and D). However, CLIP‐HA cKD parasites grown under conditions lacking ATc did not have a plaquing defect, while addition of ATc led to the complete failure of plaque formation (Fig 4C). These results demonstrate that CLIP is essential for completion of the parasite lytic cycle—consistent with its low phenotype score (−3.05) in our genome‐wide screens (Sidik *et al*, 2016b)—and suggests parasites tolerate a modest reduction of CLIP expression. However, invasion assays showed that the lytic cycle arrest following CLIP knockdown is likely due to an inability of parasites to enter host cells (Fig 4D), resembling CLAMP knockdown.

### SPATR and CLIP are absent from micronemes upon CLAMP knockdown

Many single‐pass transmembrane microneme proteins are released from the plasma membrane by parasite‐encoded rhomboid proteases (Opitz *et al*, 2002; Dowse *et al*, 2005). However, CLIP lacks a typical rhomboid protease cleavage site (Sheiner *et al*, 2010), raising the possibility that the extracellular domain is cleaved outside of the predicted C‐terminal transmembrane helix. With our anti‐CLIP‐N antibody, we immunoblotted the ESA fractions from our MS‐ESA assay, detecting two major ~20 kDa bands in the ESA fraction. These bands are lost when CLIP is depleted by ATc treatment (Fig EV4E). This reveals the N‐terminal ectodomain of CLIP, which putatively binds SPATR (Figs 3K and EV3D), is likely released from the parasite plasma membrane into the supernatant.

To assess whether CLIP affects secretion or stability of additional microneme proteins, as observed for CLAMP, we performed whole‐proteome profiling and an MS‐ESA assay of the CLIP‐HA cKD strain (Fig 4E). CLIP depletion specifically destabilized CLAMP, and to a lesser degree SPATR in parasite lysates (Fig 4F). As with the CLAMP MS‐ESA, microneme proteins were found in the calcium‐dependent ESA fraction of zaprinast‐induced CLIP‐HA cKD confirming intact microneme secretion in the absence of CLIP (Fig EV4F). During CLIP knockdown, the protein most dramatically depleted from the ESA fraction was CLIP itself, with a modest effect on SPATR secretion (Fig 4G); however, CLAMP was not detected in this experiment.

**Figure EV5:**
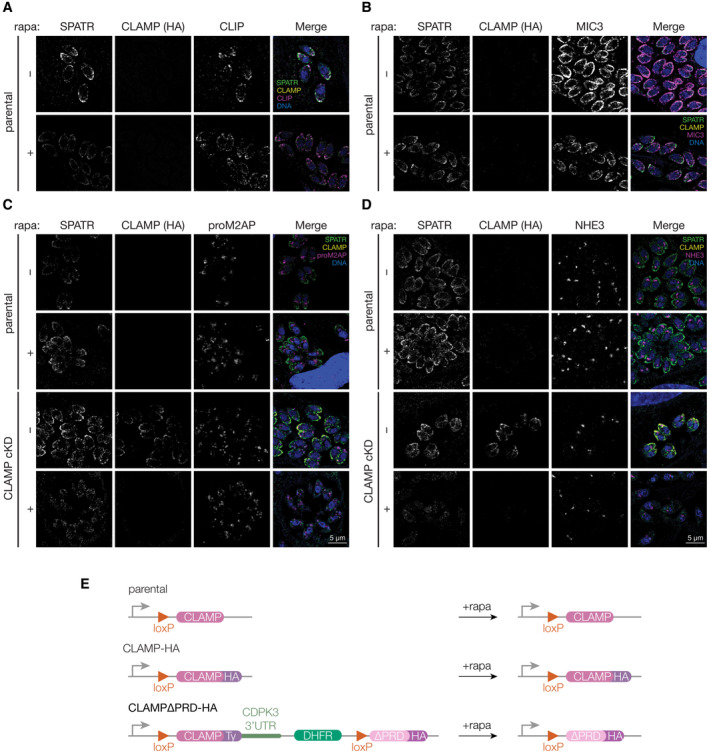
SPATR trafficking is affected by CLAMP knockdown AThe SPATR and CLIP localization is unchanged regardless of rapamycin or vehicle treatment in the parental strain for CLAMP‐HA cKD. Complements Fig 4H.BThe SPATR and MIC3 localization is unchanged regardless of rapamycin or vehicle treatment in the parental strain for CLAMP‐HA cKD. Complements Fig 4I.C, DProbing parental and CLAMP‐HA cKD with anti‐SPATR upon rapamycin treatment reveals partial overlap with anti‐proM2AP when SPATR is mislocalized following CLAMP knockdown (C). However, there is no overlap between mislocalized SPATR signal and NHE3 when CLAMP is knocked down (D).EA schematic for CLAMP locus before and after rapamycin treatment in the three strains used for the CLAMP^ΔPRD^ experiments (Fig 5G–J).

While CLAMP and CLIP stability depends on both proteins being present (Figs 2G and 4F), SPATR appeared largely stable despite its lowered abundance in the ESA fraction (Figs 2F and 4G). Using the CLAMP‐HA cKD strain we tracked the effect of removing CLAMP and CLIP on SPATR's trafficking. As expected, CLAMP‐HA and CLIP were both depleted upon CLAMP knockdown. SPATR, however, was only modestly reduced in expression, but no longer localized to micronemes (Fig 4H and I). Treatment of the parental untagged strain with rapamycin had no effect on the localization of SPATR, CLIP, or MIC3 (Fig EV5A and B). To determine where SPATR was being mislocalized, we probed for proM2AP, a marker of the trans‐golgi network (TGN) and early endosomes (Harper *et al*, 2006). Upon CLAMP knockdown, the remaining SPATR signal predominantly shifts to internal, non‐micronemal puncta that partially overlap with proM2AP (Fig EV5C), but were distinct from the endosome‐like compartments labeled by NHE3 (Fig EV5D) (Francia *et al*, 2011). These results suggest that in the absence of CLAMP or CLIP trafficking of SPATR is compromised.

### The CLAMP complex is necessary for rhoptry secretion

**Figure 5:**
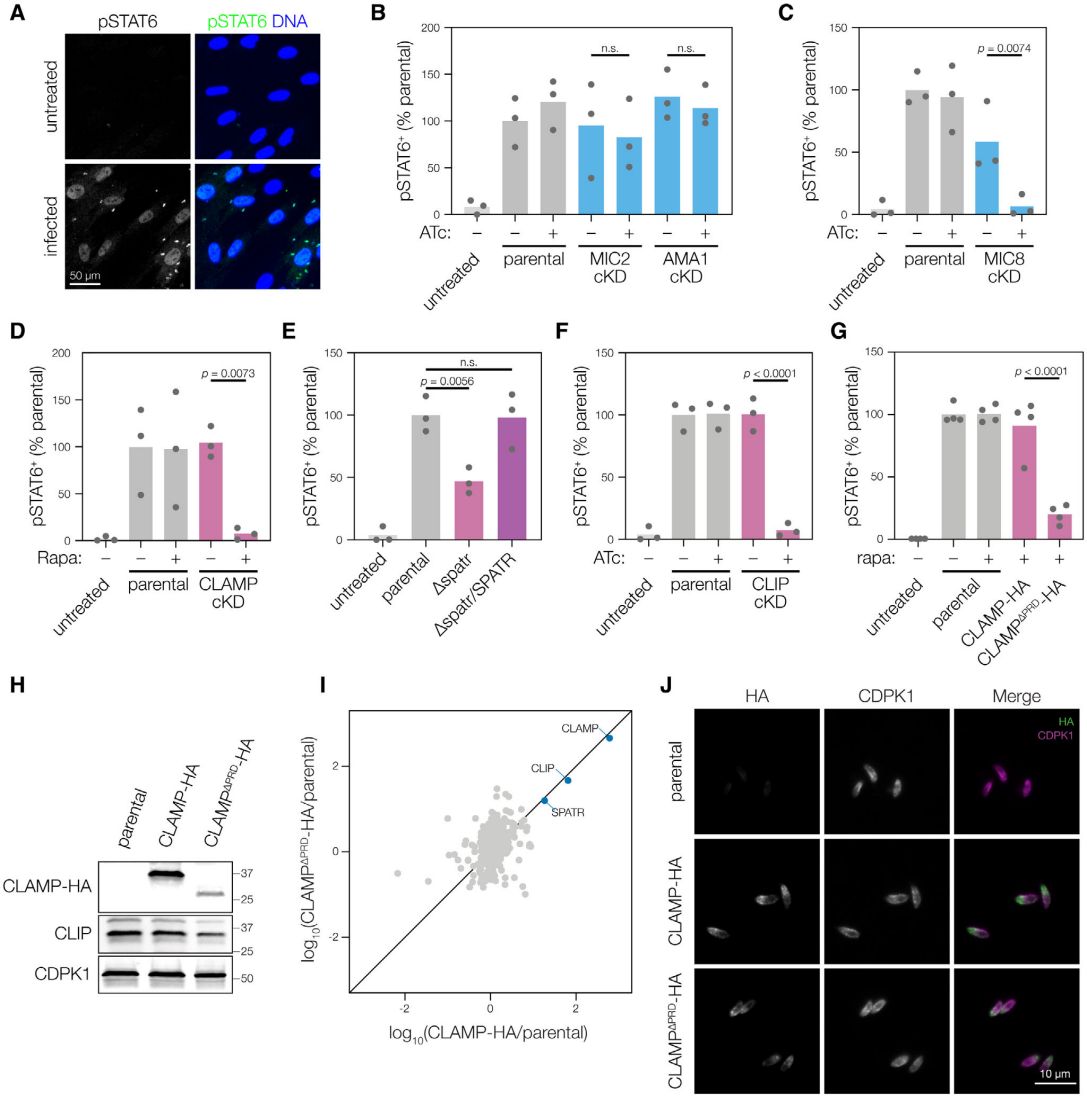
All members of the CLAMP complex are necessary for robust secretion of rhoptry contents AExamples of HFF cells stained with anti‐pSTAT6 and Hoechst (to visualize DNA) after treatment with mycalolide B‐treated parasites. Green‐fluorescent parasites appear as small dots in the pSTAT6 channel.B, CAnhydrotetracycline (ATc) treatment did not significantly change the ability of MIC2‐cKD or AMA1‐cKD parasites to elicit pSTAT6‐positive host cell nuclei (B). However, ATc treatment significantly reduced the ability of MIC8‐cKD parasites to elicit pSTAT6‐positive host cell nuclei (C). Mean is plotted for *n* = 3 biological replicates; *P* values derive from one‐way ANOVA followed by Sidak's multiple comparison test prior to data normalization.D–FAll members of the CLAMP complex are required for robust nuclear translocation of host pSTAT6. Mean is plotted for *n* = 3 biological replicates; *P* values derive from one‐way ANOVAs followed by Sidak's multiple comparison tests.GSwapping CLAMP for CLAMP^ΔPRD^ blocks rhoptry secretion measured by nuclear pSTAT6. Mean is plotted for *n* = 4 biological replicates; *P* values derive from one‐way ANOVAs followed by Sidak's multiple comparison tests.HCLAMP^ΔPRD^ is expressed at the expected size. Parental, CLAMP‐HA, and CLAMP^ΔPRD^‐HA are all treated with rapamycin.ICLAMP‐HA and CLAMP^ΔPRD^‐HA both co‐immunoprecipitate similarly with CLIP and SPATR. The log_10_(abundance ratio) for both HA‐tagged CLAMP constructs is relative to the parental IP.JCLAMP‐HA and CLAMP^ΔPRD^‐HA both have the same microneme localization in extracellular parasites. Source data are available online for this figure.

We examined whether CLIP and SPATR are also necessary for rhoptry secretion, like CLAMP. Because the morphology of evacuoles is extremely variable, counting these structures is subject to investigator bias. Others have measured rhoptry secretion by assessing the phosphorylation of the host transcription factor STAT6, which is targeted by the rhoptry kinase ROP16 following secretion into host cells (Saeij *et al*, 2006; Ong *et al*, 2010; Butcher *et al*, 2011; Lamarque *et al*, 2014). We incubated mycalolide B–treated parasites with human fibroblasts, which we then stained for host phospho‐STAT6 (pSTAT6). Using a custom analysis script, we quantified the percentage of host cells with nuclear pSTAT6 (Fig 5A) as an unbiased readout for rhoptry secretion.

We benchmarked the pSTAT6 assay using conditional mutants for various microneme proteins. The adhesin MIC2 mediates attachment to host cells during gliding motility and the moving junction component AMA1 forms stable interactions during invasion, but neither is expected to influence rhoptry discharge (Huynh & Carruthers, 2006; Lamarque *et al*, 2014). As expected, knocking down MIC2 or AMA1 did not substantially alter the number of host cells displaying nuclear accumulation of pSTAT6 (Fig 5B). On the other hand, MIC8 has been shown to be necessary for rhoptry secretion (Kessler *et al*, 2008) and its knockdown resulted in a near complete block in rhoptry discharge (Fig 5C). Based on these results we conclude that our assay robustly distinguishes between effects on rhoptry exocytosis and other mechanisms that impact invasion.

We next examined the effect of depleting different components of the CLAMP complex on rhoptry discharge. As expected from quantification of evacuoles, knockdown of CLAMP severely diminished the number of pSTAT6‐positive nuclei observed in infected cultures (Fig 5D). To examine the role of SPATR, we took advantage of strains in which *SPATR* was replaced with a chloramphenicol acetyltransferase cassette (Δ*spatr*) and later complemented (Δ*spatr/SPATR*) (Huynh *et al*, 2014). Loss of SPATR reduced the number of host cells that accumulate pSTAT6 by approximately half (Fig 5E), consistent with the modest invasion defect reported for the mutant in cell culture (Huynh *et al*, 2014) and its minimal impact on fitness in genome‐wide screens (Sidik *et al*, 2016b). During CLIP knockdown, we observed a near complete loss of pSTAT6 in host cell nuclei, replicating the phenotype seen with CLAMP knockdown (Fig 5F).

Based on the structural predictions, the C terminal proline‐rich domain (PRD) of CLAMP does not participate in complex formation and protrudes into the parasite cytosol (Fig 3K). Cytosolic domains of other microneme proteins play roles linking their adhesive properties to other cellular functions. For example, the C‐terminal domain of MIC2 and related adhesins is necessary for contacting the glideosome and driving motility (Kappe *et al*, 1999; Starnes *et al*, 2006; Jacot *et al*, 2016), raising the possibility that the CLAMP complex binds host cells and transmits a rhoptry secretion signal via the cytosolic PRD of CLAMP. In a rapamycin‐inducible Cre (DiCre) background, we engineered an inducible allelic exchange system such that rapamycin treatment swaps the endogenous CLAMP sequence with an HA‐tagged copy of CLAMP lacking the PRD (CLAMP^ΔPRD^‐HA; Fig EV5F). Compared to either the parental or a strain with a wildtype HA‐tagged allele, CLAMP^ΔPRD^‐HA had severely impaired rhoptry secretion (Fig 5G). CLAMP^ΔPRD^‐HA was expressed at the expected size (Fig 5H), bound CLIP and SPATR to a similar extent as full‐length CLAMP (Fig 5I), and retained microneme localization (Fig 5J). Based on these results we conclude the PRD is essential for linking the CLAMP complex to cellular pathways that control rhoptry secretion.

### CLAMP and SPATR are essential for blood‐stage growth of *Plasmodium falciparum*

**Figure 6:**
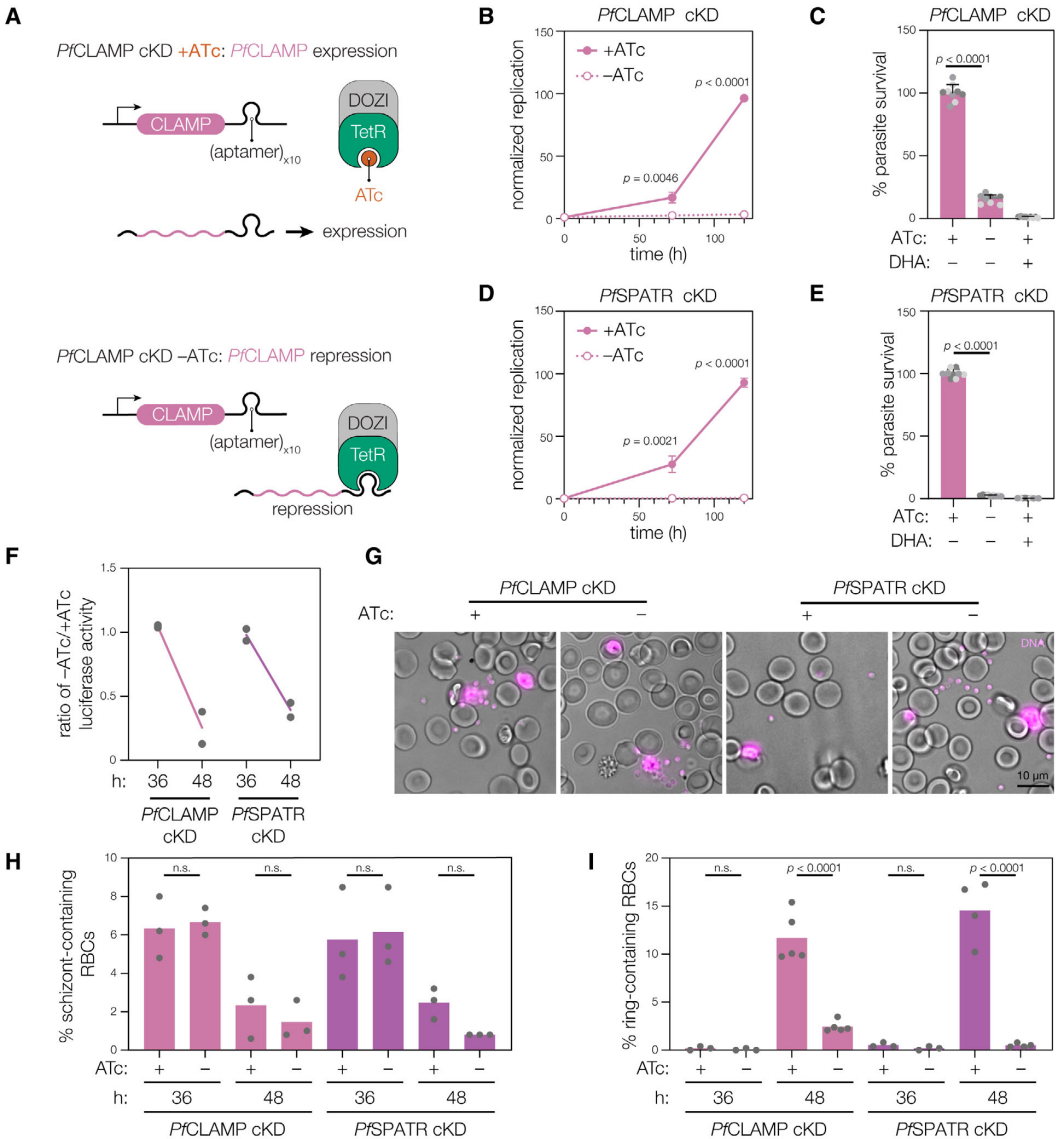
*Pf*CLAMP and *Pf*SPATR are essential and required for invasion AThe *P. falciparum* conditional knockdown system used to downregulate CLAMP or SPATR. Removal of ATc from the culture medium allows transcript binding of the TetR‐DOZI fusion protein to the Tet aptamer sequences and leads to conditional loss of protein translation.B–EConditional knockdown of *Pf*CLAMP (B and C) or *Pf*SPATR (D and E) by removal of ATc from the culture medium leads to complete loss of parasite growth. Mean with s.d. is plotted for (B) and (D) for *n* = 3 biological replicates. For (C) and (E), the mean of *n* = 3 biological replicates is plotted, which were normalized to the +ATc condition mean of three technical replicates for each experiment (technical replicates displayed, shaded by biological replicate).FKnockdown of *Pf*CLAMP or *Pf*SPATR by removal of ATc does not impair growth of ring‐stage synchronized parasites at 36 h (schizonts) after invasion, but does at 48 h (new rings). Mean is plotted for *n* = 2 biological replicates.GExtracellular merozoites are seen next to schizonts in synchronized parasite cultures regardless of *Pf*CLAMP or *Pf*SPATR knockdown. Single plane of a Z‐stack taken of live culture. DNA is visualized by DRAQ5 addition to culture.H
*Pf*CLAMP and *Pf*SPATR knockdown does not lead to a difference in schizont numbers at both 36 or 48 h post invasion in synchronized culture. For all, the mean of *n =* 3 biological replicates are plotted.I
*Pf*CLAMP and *Pf*SPATR knockdown leads to lower numbers of ring stage parasites during the first round of RBC invasion (48 h) following ATc removal in synchronized parasite cultures. For all, the mean of *n =* 3–5 biological replicates are plotted (as indicated by number of points for each condition). Data information: Activity of constitutively expressed *Renilla* luciferase was used to track growth of each *P. falciparum* conditional knockdown line (B–F). Where individual parasite stages were counted (H and I), > 500 RBCs were counted for each biological replicate. Dihydroartemisinin (DHA) is used as a control for growth inhibition (C and E); *P* values derive from multiple *t*‐tests for each time point using the Benjamini‐Hochberg FDR approach (B and D) or one‐way ANOVA followed by Sidak's multiple comparison test (C, E, H, and I). Source data are available online for this figure.

All three members of the CLAMP complex have homologs throughout the Apicomplexa, including the causative agent of the most severe forms of malaria, *Plasmodium falciparum*. Though *Pf*CLIP has been refractory to N‐ or C‐terminal tagging, as with *Tg*CLIP, we were able to construct conditional knockdown strains for both *Pf*CLAMP and *Pf*SPATR by inserting tandem Tet repressor (TetR) aptamer sequences at the endogenous locus in a parental strain expressing the Tet repressor fused to the translational repressor DOZI (TetR‐DOZI). The strains additionally express *Renilla* luciferase. When ATc is removed from the culture media, TetR‐DOZI can bind the transcript and inhibit protein translation (Fig 6A). Monitoring *Renilla* luciferase activity as a metric for growth, we confirmed that *Pf*CLAMP is essential for blood‐stage growth of *P. falciparum* (Fig 6B and C), as previously described (Sidik *et al*, 2016b). When *Pf*SPATR expression was repressed by the removal of ATc, we found a similarly complete block of blood‐stage growth (Fig 6D and E), demonstrating that *Pf*SPATR and *Pf*CLAMP are both essential during blood‐stage growth of *P. falciparum*. To establish which stage of the life cycle is affected by loss of *Pf*SPATR or *Pf*CLAMP, sorbitol‐synchronized ring‐stage parasites were split and grown in media with or without ATc. Growth was assessed by luciferase activity at time points when wildtype parasites are predominantly schizonts (36 h) or newly invaded rings (48 h). Knockdown of *Pf*SPATR or *Pf*CLAMP had no effect on growth at 36 h, during the first intraerythrocytic cycle, but decreased the parasitemia at 48 h (Fig 6F). These results are consistent with a block in invasion resulting from knockdown of either complex subunit. Using live microscopy, we observed that synchronized schizonts egressed normally in both the presence and absence of ATc (Fig 6G, Movies [Link], [Link]). The number of schizonts decreased in all cases between 36 and 48 h post invasion, even when *Pf*SPATR or *Pf*CLAMP were knocked down, confirming parasites replicate and egress normally in the absence of these factors (Fig 6H). However, knockdown of either *Pf*SPATR or *Pf*CLAMP resulted in a significantly lower number of ring‐stage parasites during the establishment of the second intraerythrocytic cycle, at 48 h (Fig 6I). We conclude that the essentiality of both *Pf*SPATR and *Pf*CLAMP in *P. falciparum* is due to an invasion defect, most likely due to a conserved role in rhoptry secretion among apicomplexans.

## Discussion

The microneme protein CLAMP was previously identified as an invasion factor necessary for *T. gondii* and *P. falciparum* fitness (Sidik *et al*, 2016b). Here, we discover that CLAMP is part of a complex together with two additional microneme proteins: SPATR and the newly characterized protein CLIP (TGGT1_212270). All members of this complex are necessary for normal secretion of rhoptry contents—with the membrane‐associated CLIP and CLAMP being absolutely required. CLAMP knockdown parasites are unaltered in host cell adhesion, microneme secretion, or apical complex morphology, leading us to conclude that their invasion defect stems specifically from an inability to discharge rhoptries.

Microneme proteins perform diverse functions as parasites spread between host cells, including membrane disruption during egress, adhesion during gliding motility, and establishment of the moving junction during invasion. However, only a handful of microneme proteins appear to mediate rhoptry discharge. MIC8, a prototypical single‐pass transmembrane microneme protein, was the first factor shown to be specifically required for rhoptry discharge in *T. gondii* (Kessler *et al*, 2008). More recently, two large multipass transmembrane proteins (CRMPa and CRMPb) were similarly associated with rhoptry discharge and shown to form a complex with two single‐pass microneme proteins bearing thrombospondin domains (Sparvoli *et al*, 2022; Singer *et al*, 2023). Although the CRMP complex proteins do not localize to micronemes unambiguously (Barylyuk *et al*, 2020; Singer *et al*, 2023), they accumulated at the apical end of extracellular parasites (Sparvoli *et al*, 2022). Despite extensive analysis through co‐immunoprecipitation, there is no evidence that MIC8 and the CRMP complex interact with one another or with the CLAMP complex, suggesting the three interact transiently or perform independent essential roles during rhoptry discharge. The two complexes and MIC8 possess domains that could potentially interact with the surface of host cells (Kessler *et al*, 2008; Huynh *et al*, 2014; Sparvoli *et al*, 2022), implying that coordinated binding to various host cell molecules may determine the site of apical adhesion and rhoptry discharge.

The mechanics of how microneme proteins induce rhoptry content secretion have not been established. Unlike microneme or dense granule discharge, the release of rhoptry contents occurs specifically upon contact with host cells. Rhoptry discharge therefore requires transduction of molecular or physical cues that signal attachment to host cells. Our data indicate that apical attachment to host cells is indeed intact in the absence of CLAMP, placing the function of the CLAMP complex downstream from general physical attachment to host cells. Given that SPATR has host‐cell binding features like a thrombospondin‐like motif, a potential model is that during intimate interactions with host cell membranes, receptor binding by the apically localized CLAMP complex induces a transient interaction with the RSA, CRMPs, or additional unknown signaling components to induce rhoptry secretion. CLAMP was originally named for its similarity to claudins although its C terminus is distinct, possessing a cytoplasmic proline‐rich domain (PRD) in place of the PDZ‐binding motif that mediates assembly of tight junctions by most mammalian claudins (Hou *et al*, 2013). The PRD was not required for complex formation or micronemal localization, but rhoptry discharge was lost in its absence. This suggests that the CLAMP PRD might function analogously to the mammalian claudin PDZ‐binding motif, engaging local signaling networks to form a tight adhesion with another cell.

The relationship between the CLAMP complex and the RSA remains unclear. The specific identities of proteins forming the RSA have not been confirmed; however, knockdown of the non‐discharge protein Nd9 abolishes proper RSA assembly (Mageswaran *et al*, 2021). The CLAMP complex does not co‐precipitate with the Nd9 interactome in our experiments, nor in prior work (Aquilini *et al*, 2021), suggesting that CLAMP does not reside at the RSA, at least in absence of host cell apposition. By contrast, components of the CRMP complex do colocalize with the RSA prior to invasion, but dissociate from the apical end of the parasite once the moving junction is formed (Sparvoli *et al*, 2022). The transient association of the CRMP complex with the RSA led to speculation that it serves to detect and transduce the signals of host‐cell attachment, as we propose for the CLAMP complex. In the complex milieu of animal tissues, distinguishing attachment to host cells from adherence to extracellular matrix components may require the use of multiple coincident signals by the parasite. For example, the CRMP complex could sense mechanical forces in a manner analogous to the Piezo ion channels (Murthy *et al*, 2017), while the CLAMP complex may signal attachment to specific ligands on the host cell surface. Alternatively, rhoptry discharge factors may help define the site of rhoptry protein translocation.

Rhoptry discharge factors display varying conservation among alveolates. CRMPs and the Nd proteins (e.g. Nd9) were identified by their homology to factors involved in the mucocyst secretion machinery of ciliates, and show broad conservation across apicomplexans (Aquilini *et al*, 2021; Mageswaran *et al*, 2021; Sparvoli *et al*, 2022). By contrast, orthologs of MIC8 are limited to *T. gondii* and its closest relatives (Li *et al*, 2003), suggestive of more recent adaptations, perhaps mediating interactions with cell types uniquely encountered by these parasites (Boothroyd, 2009). Similarly, the complex formed by *Pf*Rh5, *Pf*Ripr, and *Pf*CyRPA is required for rhoptry discharge in *P. falciparum*, but is not found in *T. gondii*. Indeed *Pf*Rh5 displays differing essentiality even across *Plasmodium* spp., further implicating species‐specific rhoptry discharge proteins as an important determinant of host tropism (Volz *et al*, 2016; Cowman *et al*, 2017; Knuepfer *et al*, 2019). SPATR also displays incomplete conservation across the phylum—orthologs are found in *T. gondii*, *N. caninum*, and *Plasmodium* spp. (Li *et al*, 2003). Notably, *P. falciparum* SPATR fails to complement *T. gondii* SPATR, implying species‐specific functional diversification (Huynh *et al*, 2014). While *T. gondii* can survive without SPATR in cell culture, other evidence suggests the factor may be essential in mice (Huynh *et al*, 2014; preprint: Giuliano *et al*, 2023). By contrast, *Pf*SPATR is essential for *P. falciparum* invasion during blood stages. The latter corroborates prior work, which found that inhibitory antibodies to *Pf*SPATR can block *P. falciparum* invasion of blood cells (Chattopadhyay *et al*, 2003). In *P. berghei*, *Pb*SPATR is required for blood stages and sporozoites, suggesting the CLAMP complex is important for host cell invasion in multiple stages of the parasitic life cycle (Gupta *et al*, 2020; preprint: Costa *et al*, 2022; Loubens *et al*, 2023). Though we were unable to generate a conditional knockdown strain for *Pf*CLIP, the PlasmoGEM project finds that the CLIP homolog in *P. berghei* is also essential during blood stages (Bushell *et al*, 2017). Together, we predict a central function for CLAMP and CLIP in rhoptry secretion, based on their essential phenotypes and conservation throughout the Apicomplexa, with SPATR adapting and modifying the core functions of the CLAMP complex to the individual requirements of certain species.

Although CLIP orthologs are highly divergent, representatives can be identified in nearly all the parasite genomes examined based on conserved motifs. Although we did not identify CLIP homologs in gregarines or hemogregarines, they were represented in the cyst forming coccidia, monoxenic coccidia, piroplasms, hemosporidia, and cryptosporidia. While divergent in length and specific motif composition, these homologs shared at least one highly conserved motif at their C terminus, proximal to the single TM helix present in CLIP homologs. This is likely the region interacting with CLAMP, as predicted in the Alphafold model and our topology analysis, with the most conserved motifs forming what appears to be a beta‐sheet that slots into a groove in the surface‐exposed portion of CLAMP. It is noteworthy that the region of CLIP that binds SPATR in the Alphafold model is less conserved, consistent with the incomplete conservation of SPATR throughout apicomplexans. The increased diversity of SPATR and CLIP suggests they may be involved in host cell binding. This is supported by the release of SPATR and the N‐terminal region of CLIP into the ESA fraction. By contrast, the more conserved CLAMP and the C‐terminal region of CLIP help form the stable complex and mediate rhoptry discharge. In *T. gondii*, we identified two additional CLIP homologs: TGGT1_212275 and TGGT1_219742. While CLIP is regulated by the tachyzoite cell cycle in a manner consistent with other microneme proteins, TGGT1_212275 is upregulated in bradyzoites (Waldman *et al*, 2020). TGGT1_219742 is expressed at very low levels throughout the asexual life cycle but appears to be upregulated in merozoites within the definitive host (Hehl *et al*, 2015; Ramakrishnan *et al*, 2019; Waldman *et al*, 2020). Intriguingly, this pattern of conservation is reminiscent of homologs of the moving junction components AMA1 and RON2, which are upregulated during different stages in the parasite life cycle (Lamarque *et al*, 2014). Other cyst‐forming coccidians have at least two CLIP homologs, whereas apicomplexans outside this clade typically possess a single homolog. As has been speculated for paralogs of AMA1 and RON2 (Lamarque *et al*, 2014), the diversification of CLIP paralogs may optimize function of the CLAMP complex during invasion in different tissue or host contexts.

This work describes a novel complex of three microneme proteins (CLAMP, CLIP, and SPATR) that mediates rhoptry content secretion in *T. gondii*. Patterns of conservation and essentiality in *Plasmodium* spp. lead us to conclude this complex likely performs analogous functions during invasion of host cells by other apicomplexan parasites. The discovery of the CLAMP complex will provide a foundation for future work aiming to identify the specific host and parasite interactions that trigger rhoptry secretion and the mechanisms underlying apicomplexan invasion.

## Materials and Methods

### *T. gondii* parasite maintenance and strain construction

*T. gondii* parasites from the strain RH and derived strains were cultured in human foreskin fibroblasts using Dulbecco's Modified Eagle's Medium (DMEM) supplemented with 3% fetal bovine serum or calf serum and 10 μg/ml gentamicin. Parasites and host cells were maintained at 37°C with 5% CO_2_. Pyrimethamine was used at 3 μM, xanthine (XA) was used at 50 μg/ml and mycophenolic acid (MPA) was used at 25 μg/ml.

CLAMP‐cKD was constructed by transfecting pCLAMP‐U1 (Sidik *et al*, 2016b) linearized with MfeI into Δku80/DiCre parasites (Pieperhoff *et al*, 2015) and selecting for integration with XA/MPA. Positive clones were isolated by limiting dilution and integration of the U1 construct was confirmed with immunofluorescence microscopy and western blotting using an anti‐HA antibody (Biolegend cat. no. 901501). Parasite lysate was not boiled prior to western blotting, as we found that boiling reduced our ability to detect CLAMP. CLAMP depletion was induced by treating parasites with 50 nM rapamycin for 2 h at least 24 h prior to analysis.

All other strains were constructed using CRISPR‐mediated homologous recombination, as described previously (Sidik *et al*, 2014). We added HA tags to CLIP in CLAMP‐Ty (Sidik *et al*, 2016b) and Δku80/Tati (Meissner *et al*, 2002) parasites by transfecting these strains with pU6‐Universal encoding a protospacer with the sequence CAGATGTGACTACCATTCGA along with a repair oligonucleotide made by duplexing the primers P1 and P2 (Table EV1). To integrate the TetO7SAG4 promoter upstream of CLIP‐HA in Δku80/Tati parasites, we amplified TetO7SAG4 from pDTS4 (van Dooren *et al*, 2008) using the primers P3 and P4. We transfected this PCR product into Δku80/Tati/CLIP‐HA parasites along with pU6‐Universal encoding a protospacer with the sequence GGTCAAGTACAGGAGTAATG and selected for integration. Positive clones were identified by PCR using the primers P5 and P6, and isolated using limiting dilution. A region spanning the junction the 3′ end of the Tet07SAG4 insert and the 5′ end of CLIP was amplified using the primers P7 and P8, and the junction was confirmed to be correct by Sanger sequencing using the primer P9. CLIP depletion was induced by treating parasites with 0.5–1 μg/ml anhydrotetracycline (ATc) for at least 24 h prior to analysis. For quantification of CLIP‐HA levels in the parental (CLIP‐HA) and cKD strain (CLIP‐HA cKD), both strains were treated with vehicle or ATc when infecting fresh 12.5 cm^2^ flasks of HFF cells. Lysed parasites were counted and harvested 2 days later into SDS–PAGE sample buffer with added benzonase. Parasite counts were normalized, run by SDS–PAGE, transferred to nitrocellulose, and immunoblotted with mouse anti‐HA and guinea pig anti‐CDPK1 (as a loading control). Fiji was used to quantify band intensities, which were then normalized to CDPK1, and analyzed using GraphPad Prism. Analysis of CLIP‐HA expression level was performed in triplicate (*n* = 3).

CLAMP‐Ty/SPATR‐HA parasites were constructed by transfecting CLAMP‐Ty parasites with pU6‐Universal carrying a protospacer with the sequence CAAGCAGAGAACCATTCTGA along with a repair oligonucleotide made by duplexing primers P10 and P11. Positive clones were isolated using limiting dilution and confirmed using immunofluorescence microscopy.

To insert a loxP site upstream of the CLAMP coding sequence, Δku80/DiCre parasites (Pieperhoff *et al*, 2015) were transfected with pU6‐Universal containing a protospacer with the sequence GGTCCGCTTTCCCCCGCCAA targeting a site 72 bp upstream of the start codon, along with a repair template made by PCR‐assembling 3 primers (P18‐20). The repair template contained the loxP site along with 40 bp 5′ and 3′ overhangs, and correct integration was verified by PCR and sanger sequencing after subcloning by limiting dilution. To make the CLAMP tagged strains in this background, a similar approach to making the CLAMP‐cKD strain was used, with linearized vectors containing a protospacer targeting the C terminus of CLAMP, a Ty tag, a DHFR selection marker, and a loxP preceding either a complement wild‐type or the ΔPRD sequence C terminally tagged with HA. These were co‐transfected with a Cas9‐expressing vector, and selected for integration with pyrimethamine, and single clones isolated by limiting dilution. After limiting dilution, the clones containing the wild‐type complement vector were found to have excised the CLAMP‐Ty and only expressed CLAMP‐HA, regardless of rapamycin treatment, presumably due to basal DiCre activity during subcloning. The stain with loxP‐CLAMP‐Ty‐loxP/CLAMP^ΔPRD^‐HA integrated behaved as expected upon rapamycin treatment, swapping from CLAMP‐Ty to CLAMP^ΔPRD^‐HA expression.

### Generation of conditional knockdown *P. falciparum* lines

Conditional knockdown (cKD) *P. falciparum* parasites were generated for *Pf*CLAMP (PF3D7_1030200) and *Pf*SPATR (PF3D7_0212600) by fusing the coding sequences and non‐coding RNA aptamer sequences in the 3′UTR, permitting translation regulation using the TetR‐DOZI system (Ganesan *et al*, 2016; Nasamu *et al*, 2021). Gene editing was achieved by CRISPR/SpCas9 using the linear pSN054 vector containing cloning sites for the left homology region (LHR) and the right homology region (RHR) as well a gene‐specific gRNA under control of the T7 promoter. Cloning into pSN054 was carried out following previously described procedures (Ganesan *et al*, 2016; Nasamu *et al*, 2021). The vector includes V5‐2xHA epitope tags, a 10× tandem array of TetR aptamers upstream of an Hsp86 3′UTR, and a multicistronic cassette for expression of TetR‐DOZI (regulation), blasticidin S‐deaminase (selection marker) and a *Renilla* luciferase (RLuc) reporter. The LHR and re‐coded regions were cloned in‐frame with the tandem V5‐2xHA tag such that CLAMP or SPATR were tagged at their C‐terminus with the V5‐2xHA tag post‐editing, directly upstream of the Tet regulatory aptamer array embedded in the 3′UTR. All primer and synthetic fragment sequences were generated using the BioXP™ system and are included in Table EV1. The final constructs were verified by restriction digestion and sequencing.

Transfection into Cas9 and T7 RNA polymerase‐expressing NF54 parasites was carried out by preloading erythrocytes with the donor vector as previously described (Deitsch *et al*, 2001). Parasite cultures were maintained continuously in 500 nM anhydrotetracycline (ATc, Sigma‐Aldrich cat. 37919) and drug selection with 2.5 μg/ml of Blasticidin S (RPI Corp B12150‐0.1) was initiated 4 days after transfection. Cultures were monitored by Giemsa smears and RLuc measurements.

### *P. falciparum* growth assays

Assessment of proliferation rate upon *Pf*CLAMP and *Pf*SPATR knockdown used luminescence as a readout of growth. Synchronous ring‐stage parasites cultured in varying concentrations of ATc (50, 3, 1, and 0 nM) were set up in triplicate in a 96‐well U‐bottom plate (Corning, cat. 62406‐121). Dihydroartemisinin treatment (DHA; 500 nM) was used as a negative control for growth. Experiments were performed in triplicate (*n* = 3) for all growth assays. Luminescence readings were taken at 0, 72, and 120 h post‐invasion using the Renilla‐Glo® Luciferase Assay System (Promega cat. E2750) and the GloMax® Discover Multimode Microplate Reader (Promega). Luminescence values in the knockdown condition were normalized to maximum of ATc‐treated (100% growth) and dihydroartemisinin (DHA) treated (500 nM, no growth) samples and results were visualized on a bar graph using GraphPad Prism (GraphPad Software).

### Single intraerythrocytic cycle *P. falciparum* growth, egress microscopy, and ring and schizont counting

*P. falciparum* cKD strains were repeatedly synchronized to the ring stage every 48 h by 5% sorbitol treatment. *Pf*CLAMP and *Pf*SPATR knockdown was induced by ATc wash out following sorbitol treatment, with this being considered the initial time of RBC invasion (0 hpi). Constitutively expressed Renilla luciferase signals were measured 36 and 48 hpi in cultures with and without ATc as a metric for parasite biomass. Luciferase experiments were performed twice for each strain (*n* = 2). Concomitantly, Giemsa‐stained smears were analyzed by microscopy to verify the synchronicity and progress of parasites through the intracellular cycle stages. For ring and schizont counting, the relative proportion of either schizont‐containing RBCs or newly invaded, ring‐containing RBCs was quantified at 36 and 48 hpi in the smears mentioned above, with 500 RBCs counted across 4–5 microscopy fields per condition. Statistics and plotting of ring counting and luciferase experiments was performed with GraphPad Prism (GraphPad Software).

For live microscopy of egressing parasites, 5–10% parasitemia synchronized schizont‐stage parasite cultures (corresponding to 36+ hpi in the above timeline) were diluted 25‐fold in complete media and transferred to 35 mm #1.5 polymer μ‐Dish ibiTreat imaging dishes (Ibidi, cat# 81156). For the DNA‐stained parasites still images, media additionally had 0.5 μM DRAQ5 far‐red DNA stain added (BioLegend, cat# 424101). After allowing RBCs to settle for 5–10 min, dishes were gently mounted on a 60× oil objective in a 37°C humidified sample environment chamber with a 5% O_2_, 5% CO_2_, 90% N_2_ gas mixture. For videos, 30 min bright‐field videos were collected, capturing 4 frames per second using an Orca‐Fusion BT camera. Videos and images were cropped and annotated using NIS elements (Nikon) and Fiji (Schindelin *et al*, 2012), and are rendered at 10× speed (40 frames per second).

### Immunofluorescence microscopy

To analyze CLIP and CLAMP's localization, intracellular CLAMP‐Ty and CLAMP‐Ty/CLIP‐HA parasites were fixed in 4% paraformaldehyde on ice for 20 min, then permeabilized with cold methanol for 2 min. Cells were stained with a 1:1,000 dilution of mouse anti‐Ty (Bastin *et al*, 1996), a 1:1,000 dilution of mouse anti‐MIC2 (Achbarou *et al*, 1991), or a 1:1,000 dilution of mouse anti‐HA (Biolegends cat. No. 901501) and Hoechst (Santa Cruz cat. no. 394039, 2,000‐fold dilution).

For comparative localization of CLAMP‐HA and CLAMP^*ΔPRD*^‐HA, parasites seeded into fresh HFF flasks in presence of 50 nM rapamycin. After a 2–4 incubation, rapamycin and uninvaded parasites were washed away with 2× PBS washes, a D3C wash (5 min, 37°C), a final PBS wash, followed by addition of D3C for growth. After 2 days, extracellular parasites were added to glass coverslips pretreated with poly‐L‐lysine in D10, and spun down onto coverslips at 500 *g* for 5 min. After 30 min at 37°C, As stated above for CLAMP and CLIP localization, parasites were fixed with 4% formaldehyde, permeabilized with methanol, and stained with mouse anti‐HA and guinea pig anti‐CDPK1.

To analyze trafficking of CLIP, SPATR, MIC3, NHE3, and proM2AP, CLAMP‐HA cKD parasites or the parental strain were allowed to invade HFFs grown on glass coverslips in presence of vehicle control or rapamycin and washed as noted above. Parasites were allowed to grow for approximately 44 h and fixed with 4% formaldehyde. Parasites were permeabilized with ice‐cold methanol for 2 min, and stained with a 1:1,000 dilution of rat anti‐HA (Roche, clone 3F10), a 1:250 dilution of mouse anti‐SPATR (Huynh *et al*, 2014), a 1:5,000 dilution of rabbit anti‐CLIP‐N, a 1:100 dilution of rabbit anti‐proM2AP (Harper *et al*, 2006), a 1:100 dilution of guinea pig anti‐NHE3 (Francia *et al*, 2011), and Hoechst 33342 (1:20,000, Life Technologies H3570).

### Host cell adhesion assay

DiCre and DiCre/CLAMP‐U1‐HA parasites that switch from expressing killer red to YFP in response to rapamycin (Sidik *et al*, 2016b) were treated with 1 μM mycalolide B in Ringer's (155 mM NaCl, 3 mM KCl, 2 mM CaCl_2_, 1 mM MgCl_2_, 3 mM NaH_2_PO_4_, 10 mM HEPES, 10 mM Glucose, 1% fetal bovine serum) for 30 min at room temperature, then resuspended at 2 × 10^6^ parasites/ml in Ringer's solution lacking mycalolide B. These parasites were perfused through microfluidic channels (Ibidi cat. no. 80196) seeded with HFF cells at a rate of 20 μl/min for 5 min, then at a rate of 50 μl/min while imaging at 10× magnification every 2 s for 5 min. Videos were analyzed with a custom script that identified attached parasites, which were defined as parasites whose centers of mass did not move more than five pixels between any two consecutive frames for at least 20 s. The number of attached parasites was normalized to the average number of parasites per frame of a given video to account for small differences in parasite concentrations. When parasites were treated with rapamycin, only YFP‐expressing parasites were analyzed. HFF cells were pre‐treated with 10 mg/ml heparin in Ringer's for approximately 1 h when indicated.

### Coupled analysis of the total proteome and ESA fractions

At the time of infection, ten 15 cm plates of HFF cells were infected with the CLAMP‐HA cKD strain were treated with 50 nM rapamycin or vehicle control, such that there were six 15 cm tissue culture plates for rapa‐treated parasites and four for the vehicle treated condition. After 2 h, the rapamycin was removed with 3× PBS washes and a D3C wash. For the CLIP‐HA cKD strain, HFF cells were infected in presence of 0.5 μg/μl anhydrotetracycline or a vehicle control, with five 15 cm cell culture dishes infected for each treatment condition. Forty‐eight hours later, extracellular parasites were filtered using 3 μm membranes to remove host cell debris. Parasites were pelleted at 1,000 *g* for 5 min at 4°C, washed in DMEM with 0% serum (D0), centrifuged again and resuspended in 130 μl D0 for each plate of parasites. For the ESA fraction experiments, 75 μl of parasites for each condition were then pretreated with 75 μl of 300 μM BAPTA‐AM in D0 or a DMSO vehicle control in 1.5 ml microcentrifuge tubes, and incubated in a 37°C water bath for 15 min. For the ESA fractions, 75 μl of 750 μM zaprinast or a DMSO vehicle control was added to each tube, then tubes were further incubated in a 37°C water bath for 30 min. The parasites were then spun down (4°C, 1,000 *g*, 5 min) and the ESA‐containing supernatant was pipetted into a protein LoBind tube. The supernatant was then spun down and the supernatant removed a second time to a new protein LoBind tube to ensure removal of all parasites.

During the zaprinast incubation, the remaining parasites not used for generation of the ESAs were spun down and lysed by resuspension in Ringer's containing 0.8% Igepal CA‐630 (155 mM NaCl, 3 mM KCl, 2 mM CaCl_2_, 1 mM MgCl_2_, 3 mM NaH_2_PO_4_, 10 mM HEPES, 10 mM Glucose, 0.8% Igepal CA‐630, Halt protease inhibitors (Life Technologies cat. no. 87786)).

For MS analysis, parasite lysates were treated with 25 units of benzonase nuclease for 10 min at room temperature, then both lysate and ESA samples were desalted and cleared of detergents using hydrophilic and hydrophobic SP3 beads (GE Healthcare cat. no. 45152105050250 and 65152105050250) as previously described (Hughes *et al*, 2019). Proteins were resuspended in 50 mM triethylammonium bicarbonate (TEAB, Sigma cat. no. T7408), reduced for 1 h at 55°C using 10 mM tris(2‐carboxyethyl)phosphine (TCEP, Pierce cat. no. 20490), alkylated for 1 h at 24°C using 25 mM iodoacetamide (CLAMP‐HA cKD experiments, Sigma cat. no. I1149) or methyl methanethiosulfonate (CLIP‐HA cKD experiments, Pierce cat. no. 23011), then digested overnight using sequencing grade modified trypsin (Promega cat. no. V5113). Peptides were incubated for 1 h at 22°C in the presence of a 2:1 mass ratio of TMT10plex reagent (Life Technologies cat. no. 90111) to peptides, and excess TMT10plex reagent was quenched using addition of hydroxylamine to 0.3%. ESA samples were combined and desalted using a Sep‐Pak Light cartridge (Waters cat. no. WAT023501) while lysate samples were combined, then desalted and fractionated using a reversed‐phase fractionation kit (Pierce cat. no. 84868). Peptides were analyzed using a Thermo Orbitrap Exploris 480 Quadrupole‐Orbitrap mass spectrometer, and downstream analysis was performed using Proteome Discoverer Version 2.4. The results of total proteome and ESA fraction analysis can be found in Dataset EV2.

For immunoblot analysis of ESA fractions of CLIP‐HA cKD, the indicated ESA supernatant was boiled in SDS–PAGE sample buffer and 3 μg of each sample was run with a 4–15% TGX gradient gel (Bio‐Rad cat. no. 4561086), then transferred to a nitrocellulose membrane. The membrane was immunoblotted with anti‐CLIP‐N and anti‐MIC2 (Achbarou *et al*, 1991).

### Anti‐CLIP‐N antibody generation

*E. coli* codon optimized CLIP coding sequence (TGGT1_212270) for residues 1–187 was gibson cloned into pVP57K, resulting in a His‐MBP‐TEV‐CLIP‐1_187 expression construct. For protein expression pVP57K‐CLIP‐1_187 was transformed into BL21 Star (DE3) *E. coli* (Invitrogen cat. no. K10101), then grown with shaking at 37°C in LB media to an optical density at 600 nm (OD600) of between 0.6 and 0.8, then cooled quickly on ice and induced with 0.5 mM Isopropyl β‐D‐thiogalactoside (IPTG, Sigma cat. no. I6758) and allowed to incubate with shaking overnight at 16°C. The cells were pelleted by centrifugation (3,000 *g*, 4°C) and resuspended in a 50 ml tube with 40 ml of binding buffer (250 mM NaCl, 50 mM Tris 7.5, 10 mM imidazole, 2.5% glycerol), and frozen in liquid nitrogen. For purification, the cells were thawed on ice, 1 mM phenylmethylsulfonyl fluoride (PMSF, Sigma cat. no. 10837091001) was added, and the sample was homogenized with a microfluidizer (Microfluidics M‐110L). The soluble fraction was separated from debris by centrifugation (9,000 *g*, 4°C) and incubated for 60 min with Ni‐NTA beads (Qiagen, cat. no. 30230) pre‐equilibrated in binding buffer. The resin was captured on a gravity flow column and washed with > 100 ml wash buffer (binding buffer with 35 mM imidazole). His‐MBP‐TEV‐CLIP‐1_187 was eluted from the resin by 2 ml volumes of elution buffer (binding buffer with 300 mM imidazole) until elution was complete. The protein was then dialyzed overnight to remove the imidazole (250 mM NaCl, 50 mM Tris pH7.5, 2.5% glycerol, 0.25 M EDTA, 0.5 mM DTT), concentrated using a 10,000 MWCO spin concentrator (Amicon) to > 2 mg/ml, the particulates were removed with a 0.22 μm spin filter (Corning cat. no. 8160) and the protein was aliquoted into ~200 μg samples and stored at −80°C.

Custom rabbit anti‐CLIP‐N antiserum was made by Covance Laboratories Inc. Briefly, 250 μg of His‐MBP‐TEV‐CLIP‐1_187 protein emulsified with Freund's Complete Adjuvant was used for the primary immunization, followed by three boosts of 125 μg antigen with Freund's Incomplete Adjuvant at periods of 21 days starting from the initial immunization (day 0). The final termination bleed is referred to as anti‐CLIP‐N and reactivity towards endogenous CLIP confirmed by immunoblot.

### Electron microscopy

Extracellular parasites were treated with 1 μM mycalolide B or 1 μM cytochalasin D in invasion media (sodium bicarbonate‐free DMEM containing 20 mM HEPES and 1% fetal bovine serum, pH 7.4) for 30 min at room temperature, then combined with K562 cells at an MOI of ~10. Parasites and host cells were incubated at 37°C for 15–30 min, then fixed for 45 min on ice with 2% OsO_4_ and 1% glutaraldehyde in 0.5× phosphate buffer (1× phosphate buffer consists of 38.4 mM K_2_HPO_4_, 161.6 mM KH_2_PO_4_, pH 6.2). Samples were rinsed in cold dH_2_O, then stained en bloc staining with 1% aqueous uranyl acetate (Ted Pella Inc.) at 4°C for 3 h. Samples were rinsed, then dehydrated in a graded series of ethanol and embedded in Eponate 12 resin (Ted Pella Inc.). 95‐nm sections were cut with a Leica Ultracut UCT ultramicrotome (Leica Microsystems Inc., Bannockburn, IL), stained with uranyl acetate and lead citrate, and imaged on a JEOL 1200 EX transmission electron microscope (JEOL USA Inc.) equipped with an AMT 8‐megapixel digital camera and AMT Image Capture Engine V602 software (Advanced Microscopy Techniques).

### Evacuole and pSTAT6 assays

To visualize evacuoles, extracellular parasites were suspended in 0.5 μM cytochalasin D in endo buffer (44.7 mM K_2_SO_4_, 10 mM Mg_2_SO_4_, 106 mM sucrose, 5 mM glucose, 20 mM Tris pH 8.2, 0.35% (wt/vol) BSA), then applied to monolayers on HFF cells grown on coverslips. Parasites and HFF cells were incubated at 37°C with 5% CO_2_ for 15 min to allow the parasites to adhere to the host cells, then the media was gently replaced with 1 μM cytochalasin D in HHE (HBSS (Sigma cat. no. H2387) containing 0.1 mM EGTA and 10 mM HEPES) and parasites and host cells were incubated at 37°C with 5% CO_2_ for an additional 20 min. For quantification, cells were fixed in cold methanol for 10 min, stained with anti‐ROP1 (Bradley & Boothroyd, 1999), anti‐CDPK1 (Waldman *et al*, 2020), and Hoechst (Santa Cruz cat. no. 394039), then imaged with an epifluorescence microscope (example images in Fig EV2B). For high resolution example images of evacuole formation (Fig 2C), the cells were fixed with formaldehyde, permeabilized with 0.25% Triton X‐100, stained with anti‐ROP1, anti‐CDPK1, and Hoescht, then imaged on a Zeiss 980 confocal with a 63× objective and equipped with a Airyscan detector.

To visualize parasite‐induced nuclear accumulation of host pSTAT6, parasites were pretreated with 1 μM mycalolide B (VWR cat. no. 89165‐002) in invasion media (sodium bicarbonate‐free DMEM containing 20 mM HEPES, pH 7.4) supplemented with 1% fetal bovine serum for 30 min at room temperature, then washed three times with PBS. Parasites concentrations were then normalized to 1 × 10^6^ parasites per ml in DMEM containing 3% serum and 10 μg/ml gentamicin, and 200 μl of parasites were applied to each well of HFF cells grown in a 96‐well plates with transparent bottoms (Perkin Elmer cat. no. 6055300). Plates were centrifuged at 290 *g* for 5 min, then incubated at 37°C with 5% CO_2_ for 4 h. Cells were fixed in 4% formaldehyde on ice for 20 min, then permeabilized in PBS containing 0.25% Triton X‐100, 5% fetal bovine serum and 1% normal goat serum for 8 min at room temperature. Cells were stained using anti‐pSTAT6 (Cell Signaling cat. no. 9361S or Abcam cat. no. ab188080), anti‐CDPK1 (Waldman *et al*, 2020), and Hoechst (Santa Cruz cat. no. 394039). Cells were imaged using a Cytation 3 high‐content plate reader (BioTek), and pSTAT6‐positive host cell nuclei were counted using a custom image analysis script.

### Immunoprecipitations

For anti‐Ty immunoprecipitations, Ty antibody (Bastin *et al*, 1996) was conjugated to agarose beads carrying protein G (Life Technologies cat. no. 22851) using 22 mM DMP, then washed in 0.2 M sodium borate pH 9.0 and 0.2 M ethanolamine, 0.2 M NaCl pH 8.5. Beads were washed in 100 mM glycine pH 2.5 followed by neutralization buffer (50 mM HEPES pH 7.4, 1 mM EGTA, 1 mM MgCl_2_, 300 mM KCl, 10% glycerol) to remove excess antibody prior to use. For reciprocal immunoprecipitations, pre‐conjugated magnetic anti‐HA beads were used (Pierce 88836).

For CLAMP‐Ty and reciprocal IPs, approximately 3–5 × 10^8^ Parasites were lysed in lysis buffer (150 mM NaCl, 20 mM Tris pH 8.0, 0.1% SDS, 1% Triton X‐100, Halt protease inhibitors (Life Technologies cat. no. 87786)), then cell debris was removed by centrifugation. In the case of anti‐Ty IPs, lysates were additionally precleared by incubating with Protein G slurry for ~1 h at 4°C. Antibody‐conjugated Ty or HA beads (Pierce 88,836) that had been pre‐washed with lysis buffer were then incubated with lysates for for ~1 h at 4°C. Beads were washed in lysis buffer, then conjugated proteins were eluted using 200 mM glycine pH 2.5, which was neutralized with an equal volume of 1 M Tris pH 8.5 prior to SDS–PAGE or MS analysis (Dataset EV1).

For CLAMP‐HA, CLAMP^ΔPRD^, and parental strain anti‐HA immunoprecipitations, approximately 3–5 × 10^8^ parasites were lysed in SDS‐free lysis buffer (150 mM NaCl, 20 mM Tris pH 8.0, 1% Triton X‐100, with 1× Halt protease inhibitors), then clarified by centrifugation. Pre‐washed anti‐HA beads were added to soluble fractions and incubated at room temperature for 30 min, followed by 3× washes with lysis buffer. Elution of bound proteins from beads was performed by incubation of beads in S‐trap elution buffer (5% SDS, 50 mM TEAB, pH 7.55 using H_3_PO_4_) at 37°C for 10 min. The elution was transferred to a new tube, and the elution was repeated at 70°C. Elutions were pooled, and the standard S‐trap micro workup protocol (Protifi) was used to prepare samples for MS analysis (Dataset EV3).

### CLIP phylogenetic analysis

ToxoDB (Gajria *et al*, 2008), PlasmoDB (Aurrecoechea *et al*, 2009), and CryptoDB (Heiges *et al*, 2006) were searched for CLIP homologs. Clustal Omega (Madeira *et al*, 2019) was used to align these homologs in the PHYLIP file format, which was then used with the seqboot, proml, and consense programs from the PHYLIP phylogenetics package (Felsenstein, 1989). Bootstrapping was performed 100 times, and the input order was randomized twice when using proml. The Jones‐Taylor‐Thornton probability model was used. Output from consensus was used to draw trees in Figtree (http://tree.bio.ed.ac.uk/software/figtree/). Motifs that are shared by CLIP homologs were identified using the MEME suite for motif‐based sequence analysis (Bailey *et al*, 2009).

### Membrane association assays

Approximately 1 × 10^8^ CLAMP‐Ty or CLAMP‐Ty/CLIP‐HA parasites were passed through a 3 μm filter to remove host cell debris, then split into two samples. Parasites were pelleted and resuspended in IC buffer (1.37 M KCl, 50 mM NaCl, 200 mM HEPES, 100 mM MgCl_2_, Halt protease inhibitors) with or without 2% Triton X‐100. Samples were cycled between liquid nitrogen and a 37°C water bath three times, then disrupted five times with a dounce homogenizer (VWR cat. no. 62400‐595). Following mechanical disruption, samples were centrifuged at 100,000 RPM (~400,000 *g*) for 1 h. Supernatants were collected, and pellets were resuspended in IC buffer containing 2% Triton X‐100. Samples were immunoblotted using anti‐Ty (Bastin *et al*, 1996), anti‐Actin (Dobrowolski *et al*, 1997), anti‐SAG1 (Burg *et al*, 1988), anti‐HA (Biolegend cat. no. 901501), and anti‐Aldolase (Starnes *et al*, 2009).

### Proteinase K protection assays

A lysed 15 cm dish of CLAMP‐Ty parasites (approximately 3e8) were passed through a 5 μm filter to remove host cell debris, then washed and resuspended in 260 μl chilled PBS. Parasites were divided into twelve 20 μl aliquots. To four tubes each, 20 μl PBS with 0, 0.05, or 0.1% digitonin (Invitrogen, BN2006) was added, to make parasites in PBS with final concentrations of 0, 0.025, or 0.05% digitonin. After 3 min of incubation on ice, 10 μl of prediluted proteinase K (or PBS for untreated condition) was then added. Two concentrations of proteinase K were tested, such that the final concentration after addition to parasites was 0.5 or 0.1 μg/ml. The best combination, as measured by MIC2 cytosolic tail degradation and size was 0.025% digitonin and 0.1 μg/ml proteinase K, which are used for the results presented in Figs 1C and 3G. After 30 min in proteinase K on ice, the irreversible serine/threonine inhibitor PMSF was added to a final concentration of 5 mM (3.5 μl of 100 mM stock in 100% ethanol) to inhibit proteinase K. After 5 min, 16.5 μl of 5× SDS–PAGE sample buffer containing benzonase and 1.5 mM MgCl_2_ was added to each tube, and the samples were heated at 37°C for 10 min. Samples were then clarified by a 5 min > 16,000 *g* spin, and run out on SDS–PAGE gels followed by the indicated anti‐Ty (Bastin *et al*, 1996), anti‐MIC2 (Achbarou *et al*, 1991), and anti‐CLIP immunoblotting.

### Plaque assays

Five hundred CLIP‐cKD or parental strain parasites were applied to monolayers of HFF cells in the presence of 1 μg/ml anhydrous tetracycline or vehicle control. Cells were incubated at 37°C with 5% CO_2_ for 8 days, then washed in PBS and fixed with 100% ethanol. Monolayers were stained with crystal violet solution (2% crystal violet, 0.8% ammonium oxalate, 20% ethanol), washed with water, and air‐dried.

### Alphafold prediction

To predict the *T. gondii* (GT1) and *P. falciparum* (3D7) CLAMP, CLIP, and SPATR complex structures, the ColabFold server was used (Mirdita *et al*, 2022), using Alphafold2 and MMseqs2 default settings, and the full‐length proteins for all protein sequences. The displayed structure for both *T. gondii* and *P. falciparum* was the top‐ranked structure for each prediction. The predicted model structures were aligned and rendered using PyMOL. For both complex predictions, the unstructured loops were trimmed manually, including the CLAMP C‐terminal proline‐rich domain and the internal region of CLIP linking the N‐terminal structured region to the C‐terminal structured region.

### Invasion assays

Gene depletion was induced using either 50 nM rapamycin for DiCre/CLAMP‐HA‐U1 (CLAMP‐HA cKD) or 0.5 μg/ml anhydrous tetracycline for TATi/TetO7‐CLIP‐HA (CLIP‐HA cKD) 2 days prior to assaying invasion. 2 × 10^5^ parasites were applied to each well of a clear‐bottomed 96‐well plate (Perkin Elmer cat. no. 6055300) seeded with HFF cells. In the case of CLAMP‐HA cKD invasion assays, parasites were fluorescently labeled using the CellTrace™ CFSE Cell Proliferation Kit (Invitrogen) according to the manufacturer protocol prior to addition to HFFs. Plates were centrifuged at 290 *g* for 5 min, then incubated at 37°C for 10 min. Cells were fixed in 4% formaldehyde on ice for 20 min, blocked with 5% fetal bovine serum and 1% normal goat serum (blocking buffer) then extracellular parasites were stained with anti‐SAG1 (Burg *et al*, 1988). For CLIP‐HA cKD invasion assays, cells were permeabilized with 0.25% Triton X‐100 in PBS containing 5% fetal bovine serum and 1% normal goat serum, then all parasites were stained with anti‐CDPK1 (Waldman *et al*, 2020) and host cell nuclei were stained with Hoechst (Santa Cruz cat. no. 394039). For CLAMP‐HA cKD parasites, parasites and host cells remained intact, the CFSE stain was used to monitor intracellular parasites rather than anti‐CDPK1, and the host cell nuclei were stained with cell‐permeable Hoechst 33342 (Life Technologies H3570). Three or four wells were used for each technical replicate strain, and each biological replicate was performed at least three times. Assays were imaged using a Cytation 3 high‐content plate reader (BioTek). Intracellular parasites, extracellular parasites, and host cell nuclei were identified using a custom image analysis script, and the number of intracellular parasites was normalized to the number of host cells.

## Author contributions

**Dylan Valleau:** Conceptualization; investigation; writing – original draft; writing – review and editing. **Saima M Sidik:** Conceptualization; data curation; investigation; writing – original draft. **Luiz C Godoy:** Investigation. **Yamilex Acevedo‐Sánchez:** Investigation. **Charisse Flerida A Pasaje:** Investigation. **My‐Hang Huynh:** Investigation. **Vern B Carruthers:** Supervision; writing – review and editing. **Jacquin C Niles:** Supervision; writing – review and editing. **Sebastian Lourido:** Conceptualization; supervision; visualization; writing – original draft; project administration; writing – review and editing.

## Disclosure and competing interests statement

The authors declare that they have no conflict of interest.

## Supporting information



Expanded View Figures PDF

Table EV1

Movie EV1

Movie EV2

Movie EV3

Movie EV4

Movie EV5

Movie EV6

Dataset EV1

Dataset EV2

Dataset EV3

Source Data for Expanded View

PDF+

Source Data for Figure 1

Source Data for Figure 2

Source Data for Figure 3

Source Data for Figure 4

Source Data for Figure 5

Source Data for Figure 6

## Data Availability

The immunoprecipitation and MS‐ESA mass spectrometry data have been deposited to the ProteomeXchange Consortium via the PRIDE (Perez‐Riverol *et al*, 2022) partner repository with the identifiers: PXD044091 (http://www.ebi.ac.uk/pride/archive/projects/PXD044091) and PXD044182 (http://www.ebi.ac.uk/pride/archive/projects/PXD044182).
