# Supplementary material for: A conserved complex of microneme proteins mediates rhoptry discharge in Toxoplasma
Source: EMBO J. 2023 Oct 27;42(23):e113155. doi: 10.15252/embj.2022113155 (PMC10690463; doi:10.15252/embj.2022113155)
Supplement: Supplementary file 1 — Expanded View Figures PDF [file EMBJ-42-e113155-s003.pdf]

Expanded View Figures

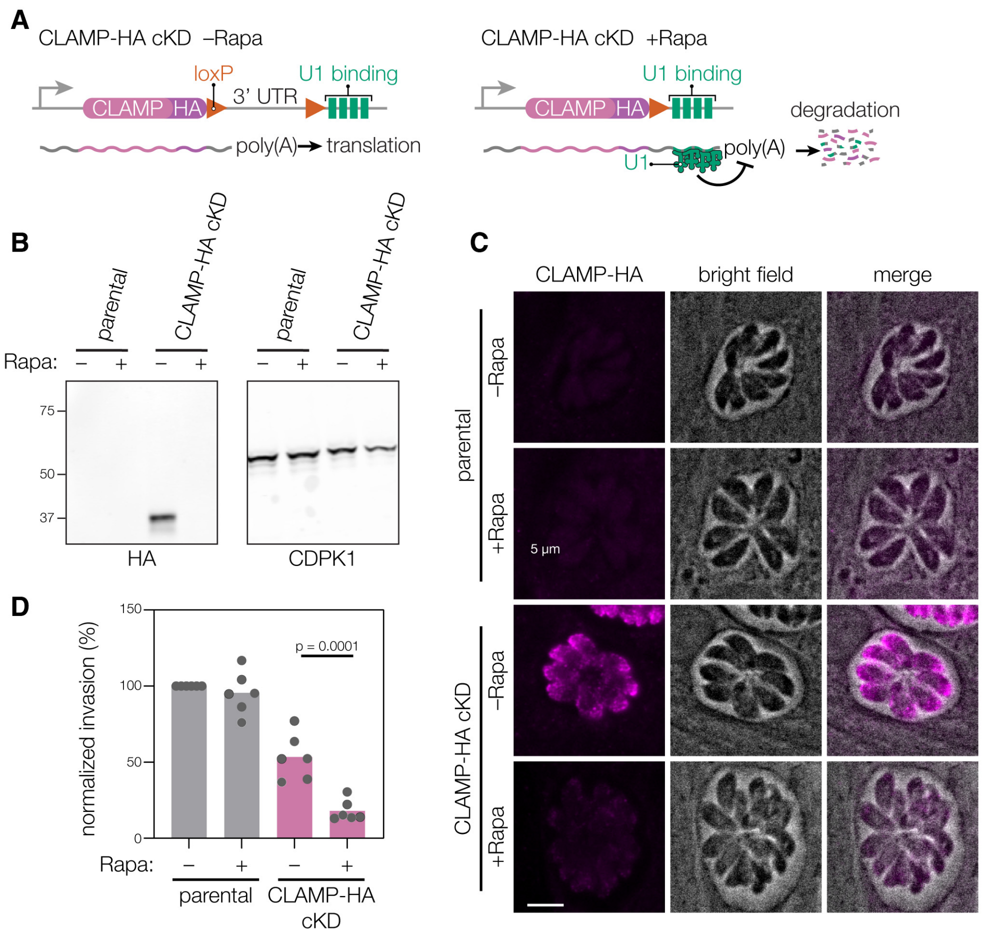

Figure EV1. Generation of a non-fluorescent CLAMP conditional knockdown in T. gondii strain.

A Diagram of the CLAMP-HA cKD scheme. Induction of DiCre dimerization by rapamycin (Rapa) treatment leads to excision of the 3′UTR between the stop codon and an array of U1 binding sites, leading to U1-mediated degradation of the mRNA transcript.

B, C Knockdown of CLAMP-HA expression was assessed following a 2 h treatment with rapamycin. Samples were analyzed 48 h after the treatment by anti-HA immunoblotting (B), or after 24 h after the treatment by anti-HA immunofluorescence (C).

D CLAMP-HA knockdown leads to inhibition of host cell invasion for parasites allowed to invade for 20 min. Normalized percent invasion refers to the number of intracellular parasites/host cell nuclei, which is then normalized to the vehicle-treated parental condition. *P* values derive one-way ANOVA followed by Sidak's multiple comparison test for $n = 6$ biological replicates.

Source data are available online for this figure.

 © 2023 The Authors

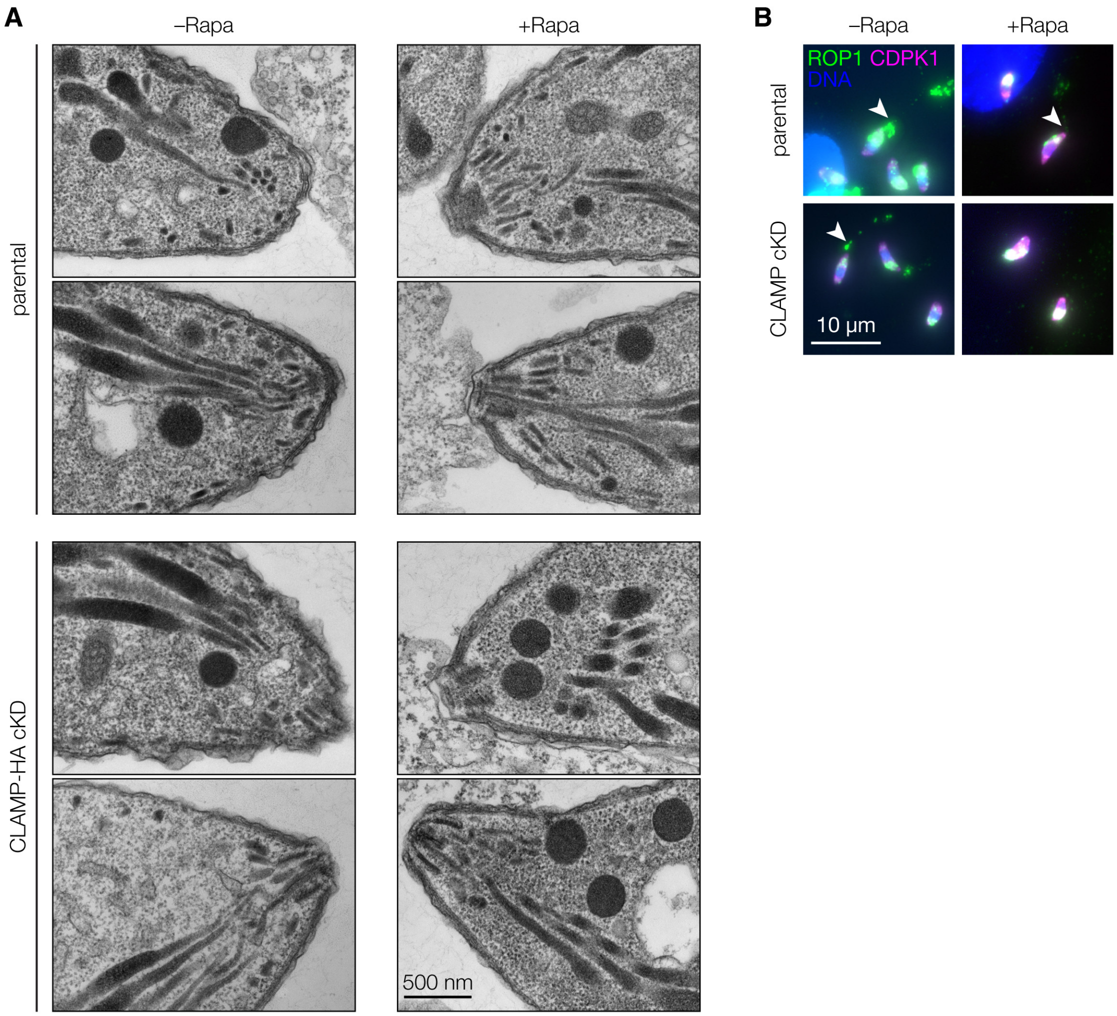

Figure EV2. CLAMP knockdown does not alter apical-complex morphology in T. gondii.

A Additional images of CLAMP-HA cKD and parental parasites prepared for electron microscopy 48 h after a 2 h treatment with 50 nM rapamycin (+Rapa) or a DMSO vehicle control (–Rapa).

B Representative images of evacuoles collected with an epifluorescence microscope and used for evacuole quantification. Arrowheads indicate examples of evacuole formation used in quantification.

Figure EV3. Extended analysis of CLIP homologs.

A Neighbor-joining phylogenetic tree of CLIP homologs. Bootstrap values for 1,000 trials are displayed. The logo plots for the top five motifs identified using MEME analysis are shown in boxes colored to match the domains on the protein models. E-value for each motif within the set of 20 CLIP homologs is indicated in parentheses. Sequence analysis suggests some of the models are incomplete (*), and a *N. caninum* sequence syntenic with TGGT1_212275 could be detected, but is not annotated in the current version of the genome (ToxoDB release 60). Abbreviated species names are provided for *Toxoplasma gondii*, *Plasmodium vivax*, *Plasmodium falciparum*,*Theileria parva*, *Theileria annulata*, *Babesia bovis*, *Babesia microti*, *Neospora caninum*, *Sarcocystis neurona*, *Hammondia hammondi*, *Eimeria tenella*, and *Cryptosporidium parvum*.

B Diagram of strains carrying HA tags on CLIP and/or SPATR used for reciprocal immunoprecipitations SPATR was tagged at its C terminus and CLIP internally, following Arg210.

C The Alphafold model of the *T. gondii* CLAMP complex showing the conserved CLIP motifs identified in EV3A. Secondary structure cartoons are displayed for CLAMP and SPATR, while for CLIP the motifs are highlighted in a space-filling model. Two views are displayed with the indicated rotation. Colors for CLIP correspond to the assigned motif colors in Fig EV3A.

D, E The *T. gondii* and *P. falciparum* Alphafold complex models for CLAMP, CLIP and SPATR share consistent interaction surfaces, with SPATR binding the N-terminal portion of CLIP (D) and CLAMP binding the C-terminal portion of CLIP (E).

Data information: For C–E, the models have been trimmed of their large unstructured regions as in Fig 3K.

© 2023 The Authors

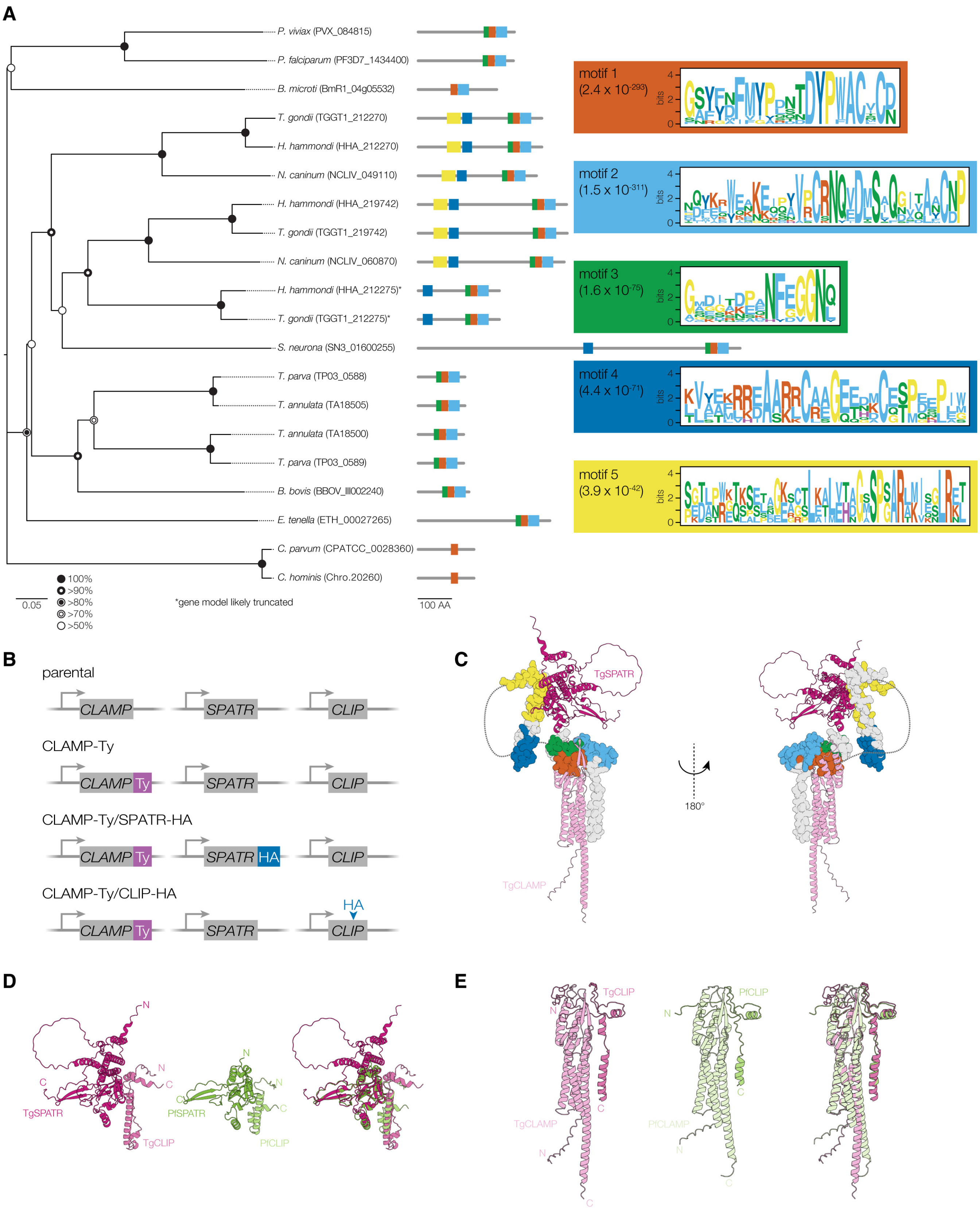

Figure EV3.

 © 2023 The Authors

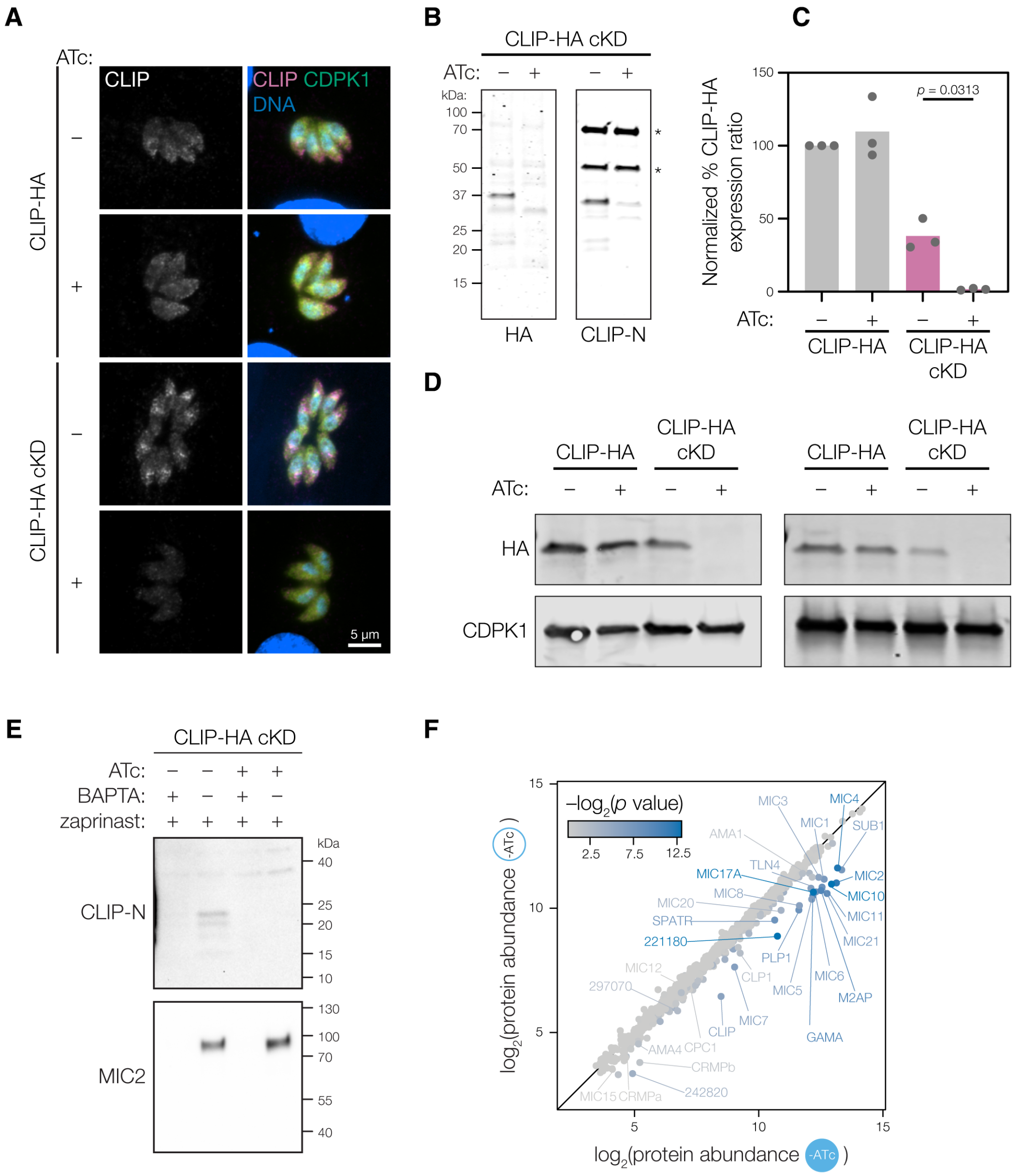

Figure EV4. Conditional CLIP knockdown disrupts microneme localization and blocks detection of CLIP in the extracellular secreted antigen fraction.

A Treatment with anhydrotetracycline (ATc) leads to loss of CLIP-HA signal in the CLIP-HA cKD strain, as visualized by anti-HA immunofluorescence.

B Further validation of CLIP knockdown with anti-HA and the anti-CLIP-N antibody generated against the N-terminal 187 residues of CLIP. Non-specific bands detected by the anti-CLIP-N serum are marked with an asterisk (*).

C, D Addition of the TetO7 operator upstream of the CLIP coding sequence leads to a reduction in basal expression of CLIP, while treatment of this strain with ATc completely blocks CLIP expression (C). Quantification of CLIP-HA and CLIP-HA cKD strains in the presence and absence of vehicle or ATc was done by western blot in triplicate, using the gel shown in Fig 4B as well as the 2 displayed in (D). *P* values in C derive from one-way ANOVA followed by Sidak's multiple comparison test for $n = 3$ biological replicates.

E Probing the CLIP-HA cKD MS-ESA samples with anti-CLIP-N confirms that a form of cleaved CLIP is found in the ESA fraction, as is the microneme protein MIC2. Knockdown of CLIP with ATc leads to loss of CLIP in the ESA fraction. As expected, microneme secretion is inhibited by BAPTA-AM and induced by zaprinast treatment.

F Microneme proteins are generally found in the ESA fraction of the vehicle-treated CLIP-HA cKD strain as shown by treatment with BAPTA-AM to inhibit secretion (*y* axis) or a vehicle control (*x* axis).

Source data are available online for this figure.

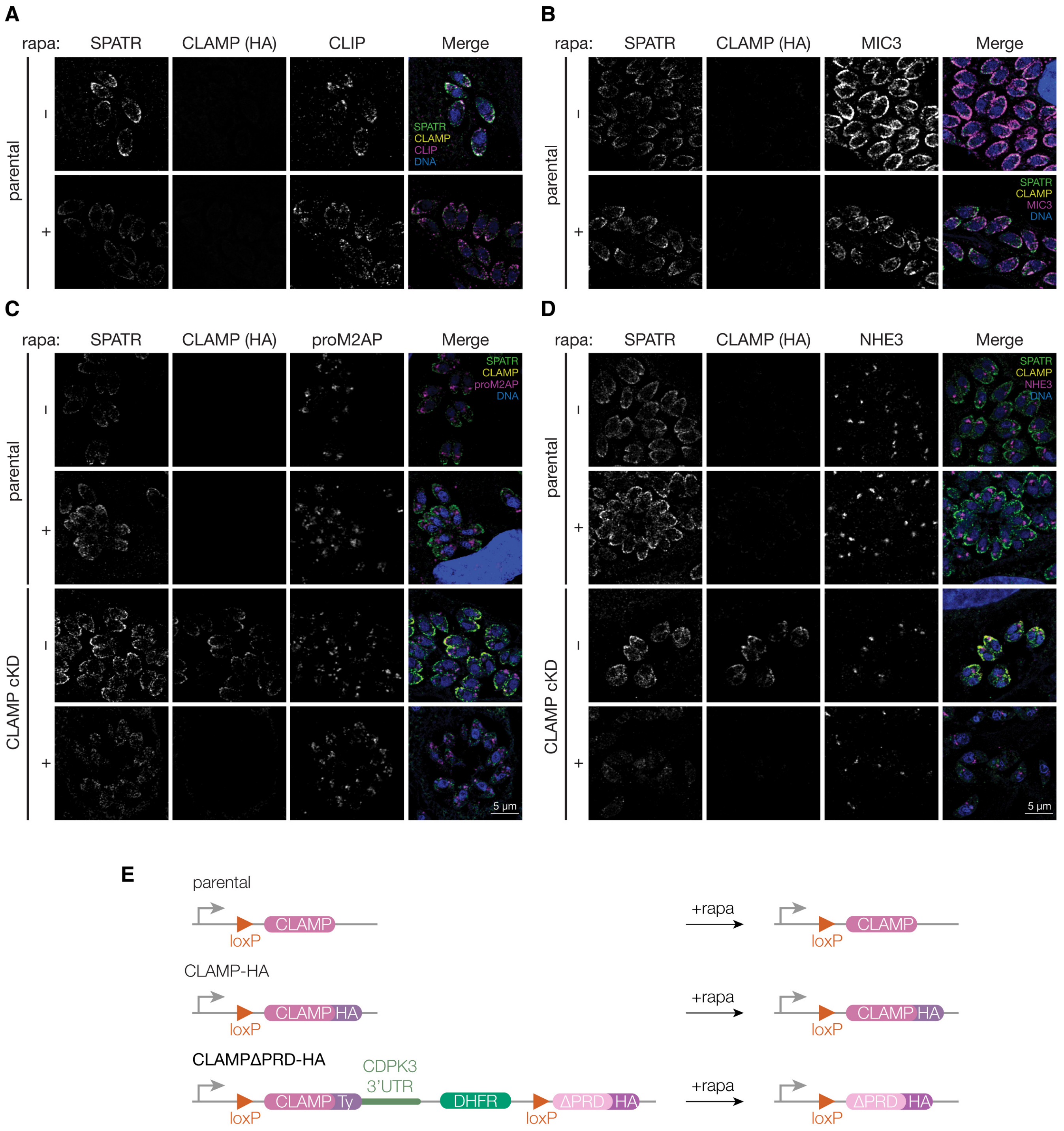

Figure EV5. SPATR trafficking is affected by CLAMP knockdown.

A The SPATR and CLIP localization is unchanged regardless of rapamycin or vehicle treatment in the parental strain for CLAMP-HA cKD. Complements Fig 4H.

B The SPATR and MIC3 localization is unchanged regardless of rapamycin or vehicle treatment in the parental strain for CLAMP-HA cKD. Complements Fig 4I.

C, D Probing parental and CLAMP-HA cKD with anti-SPATR upon rapamycin treatment reveals partial overlap with anti-proM2AP when SPATR is mislocalized following CLAMP knockdown (C). However, there is no overlap between mislocalized SPATR signal and NHE3 when CLAMP is knocked down (D).

E A schematic for CLAMP locus before and after rapamycin treatment in the three strains used for the CLAMP$^{\Delta PRD}$ experiments (Fig 5G–J).

© 2023 The Authors
