# Supplementary material for: A conserved complex of microneme proteins mediates rhoptry discharge in Toxoplasma
Source: EMBO J. 2023 Oct 27;42(23):e113155. doi: 10.15252/embj.2022113155 (PMC10690463; doi:10.15252/embj.2022113155)
Supplement: Supplementary file 2 — Table EV1 [file EMBJ-42-e113155-s016.doc]

**Table EV1.** Primers and dsDNA fragments used to generate the parasite strains in this study.

| Primer Name | Description | Sequence |
| --- | --- | --- |
| P1 | CLIP-HA repair oligonucleotide | gaagacacaagagagcaccgcagatgtgactaccattcgaTACCCATACGATGTTCCTGACTATGCGagAccaaagcgacaacgctggaagccacctgttatcaaga |
| P2 | CLIP-HA repair oligonucleotide | tcttgataacaggtggcttccagcgttgtcgctttggTctCGCATAGTCAGGAACATCGTATGGGTAtcgaatggtagtcacatctgcggtgctctcttgtgtcttc |
| P3 | Tet07SAG4 amplification | gtgcccgctaagaatgggtccaacgatattaggtcaagtacaggagtaAAGCTTCGCCAGGCTGTAAATCC |
| P4 | Tet07SAG4 amplification | gaggaacccaggcacttcagcagcaaaatgggcctcacagcgaaacatTGGTTGAAGACAGACGAAAGCAGTTG |
| P5 | Tet07SAG4 integration screening | gaaagacgacattgccgtctg |
| P6 | Tet07SAG4 integration screening | GACAGTACGCGTCTACTAAG |
| P7 | Tet07SAG4 amplification after integration | GTGGATGGACGAAGAAGACC |
| P8 | Tet07SAG4 amplification after integration | gaggaacccaggcacttcagcagcaaaatgggcctcacagcgaaacatTGGTTGAAGACAGACGAAAGCAGTTG |
| P9 | Tet07SAG4 sanger sequencing | cagaagctgcccgtctctcg |
| P10 | SPATR-HA repair oligonucleotide | ggaggtgacggagctccgatcttgcaatcagccttcgtctTACCCATACGATGTTCCTGACTATGCGtaaaaaagctttcagaatggttctctgcttggcacttccc |
| P11 | SPATR-HA repair oligonucleotide | gggaagtgccaagcagagaaccattctgaaagcttttttaCGCATAGTCAGGAACATCGTATGGGTAagacgaaggctgattgcaagatcggagctccgtcacctcc |
| P12 | CLAMP cKD RHR forward sequence | gtacggtacaaacccggaattcgagctcggCATTTGATTCACAGAAACCAT |
| P13 | CLAMP cKD RHR reverse sequence | aagacgagagattgggtattagacctagggataacagggtaatATTTTTTTAAACCAGTGTTGCTATC |
| P14 | CLAMP sgRNA target site | GAATATTGCCTTGATTGTTG |
| P15 | SPATR cKD RHR forward sequence | gtacggtacaaacccggaattcgagctcggGCGTTCATGTTAAAAAAAATAAAA |
| P16 | SPATR cKD RHR reverse sequence | aagacgagagattgggtattagacctagggataacagggtaatCCATTATTAAAATTACCTGTACGG |
| P17 | SPATR sgRNA target site | CAGGCACTCCAATCTGACCA |
| P18 | CLAMP 5utr-H1-loxP Forward1 | gtttttttgatggccggtggtccgctttcccccgccaaATAACTTCGTATAGCATACATT |
| P19 | CLAMP 5utr-H1-loxP-H2 Reverse1 | agaggggttacctgATAACTTCGTATAATGTATGCTATACGAAGTTATttggcg |
| P20 | CLAMP 5utr-loxP-H2 Reverse2 | CATcctgggaaacgcttccggagagggagaggggttacctgATAACTTCGTAT |
