# Supplementary figures and images for: A conserved complex of microneme proteins mediates rhoptry discharge in Toxoplasma
Source: EMBO J. 2023 Oct 27;42(23):e113155. doi: 10.15252/embj.2022113155 (PMC10690463; doi:10.15252/embj.2022113155)

Figure 1B

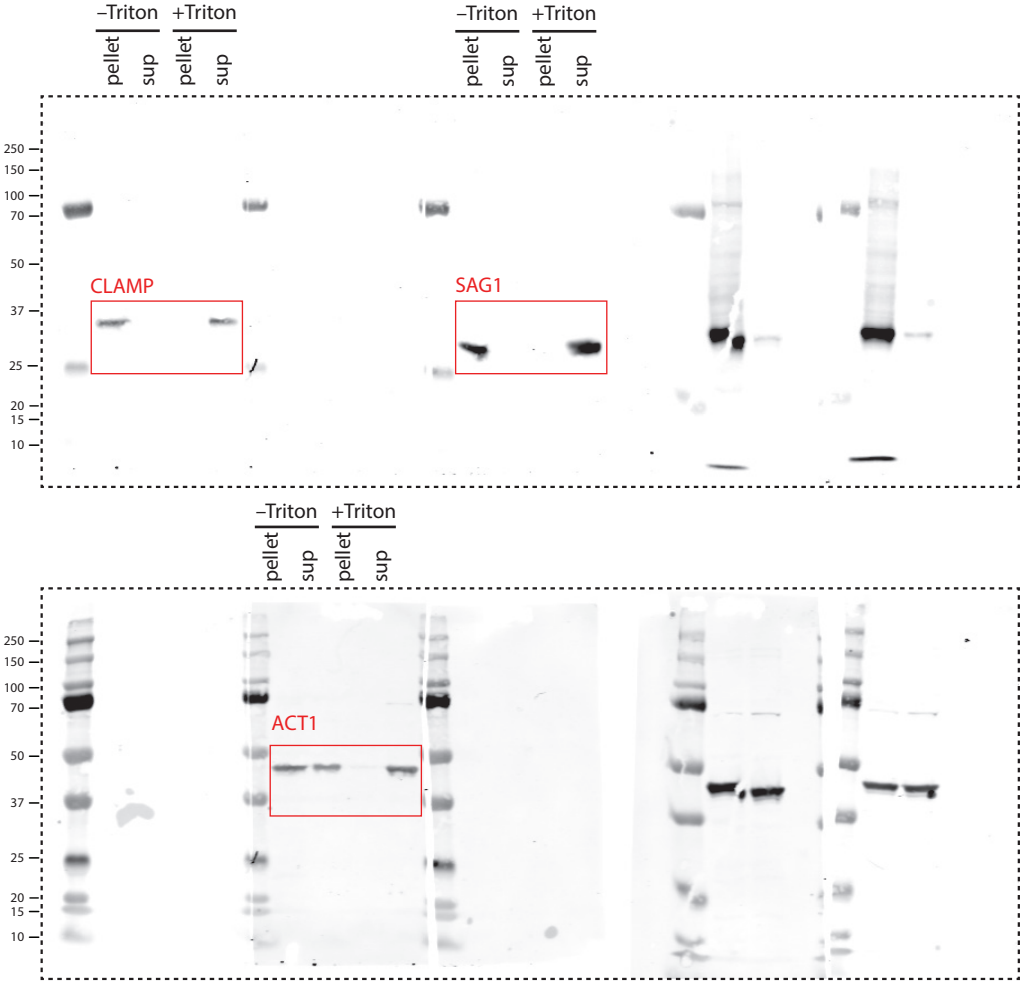

Supplement: Supplementary file 14 — Source Data for Figure 1 [file EMBJ-42-e113155-s014.zip › Figure_1/1B.pdf]

Figure 1C

|       |   |   |   |   |
|-------|---|---|---|---|
| Dig   | - | - | + | + |
| ProtK | - | + | - | + |

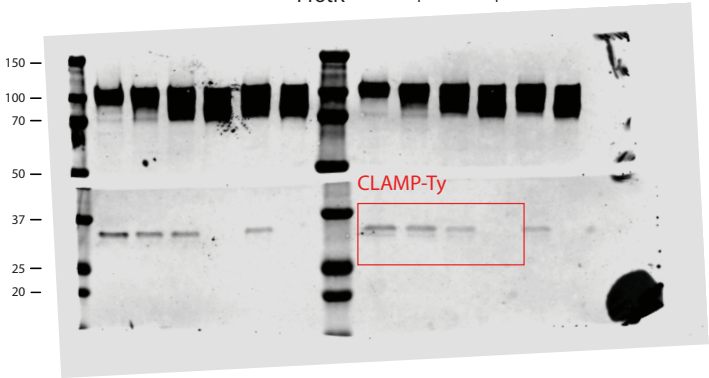

|       |   |   |   |   |
|-------|---|---|---|---|
| Dig   | - | - | + | + |
| ProtK | - | + | - | + |

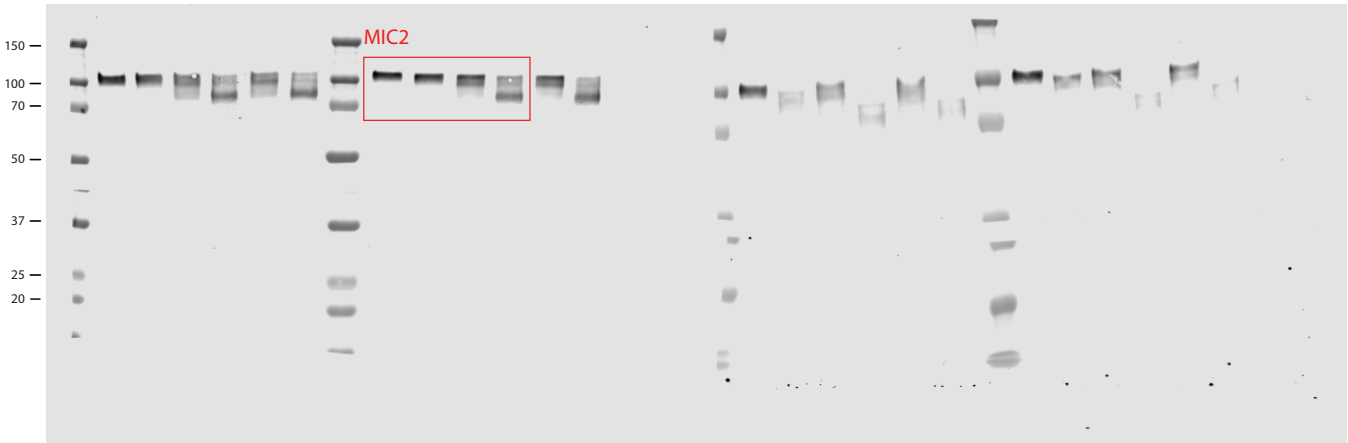

Supplement: Supplementary file 14 — Source Data for Figure 1 [file EMBJ-42-e113155-s014.zip › Figure_1/1C.pdf]

Figure 3F

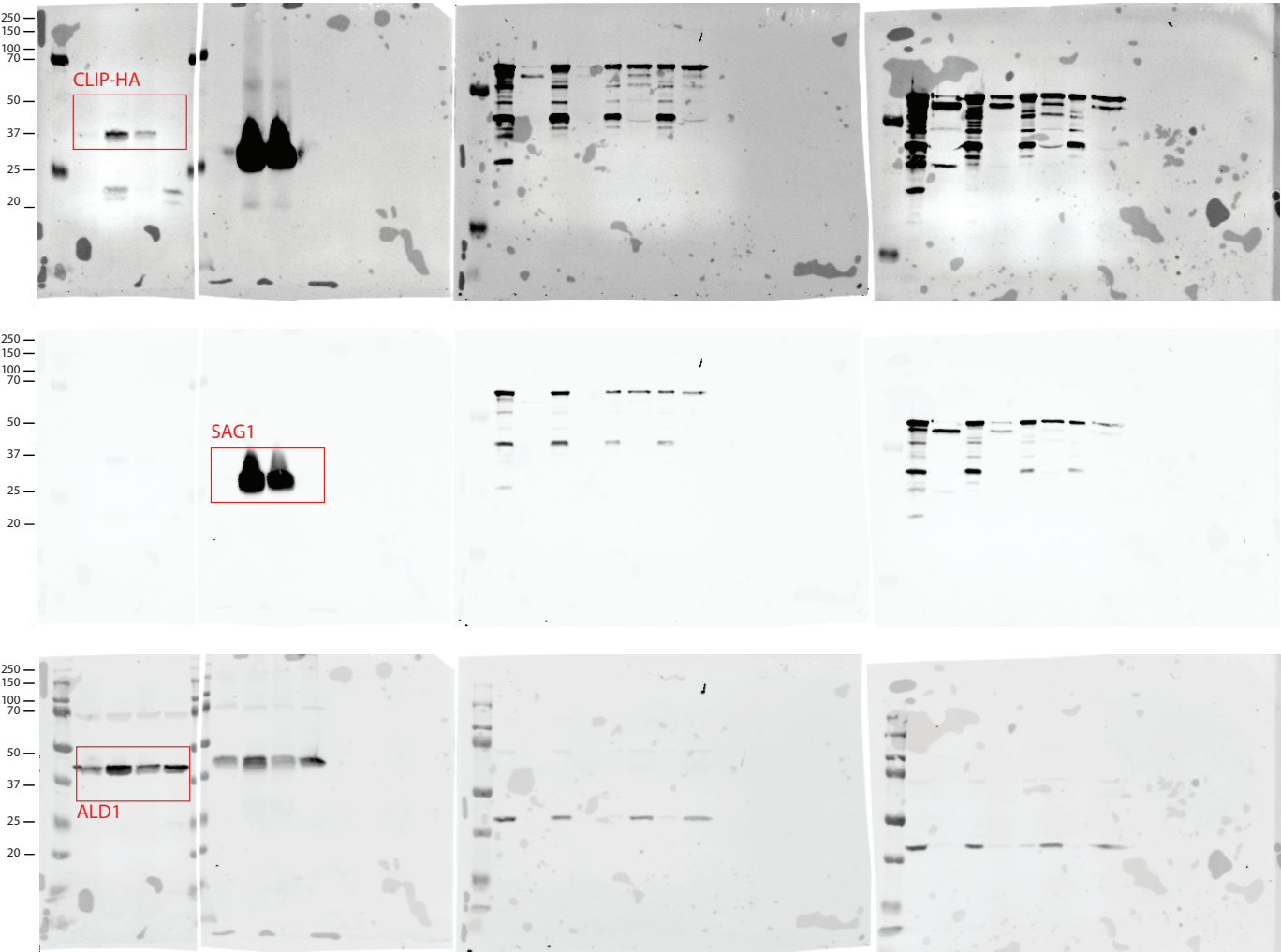

Supplement: Supplementary file 16 — Source Data for Figure 3 [file EMBJ-42-e113155-s017.zip › Figure_3/3F.pdf]

Figure 3G

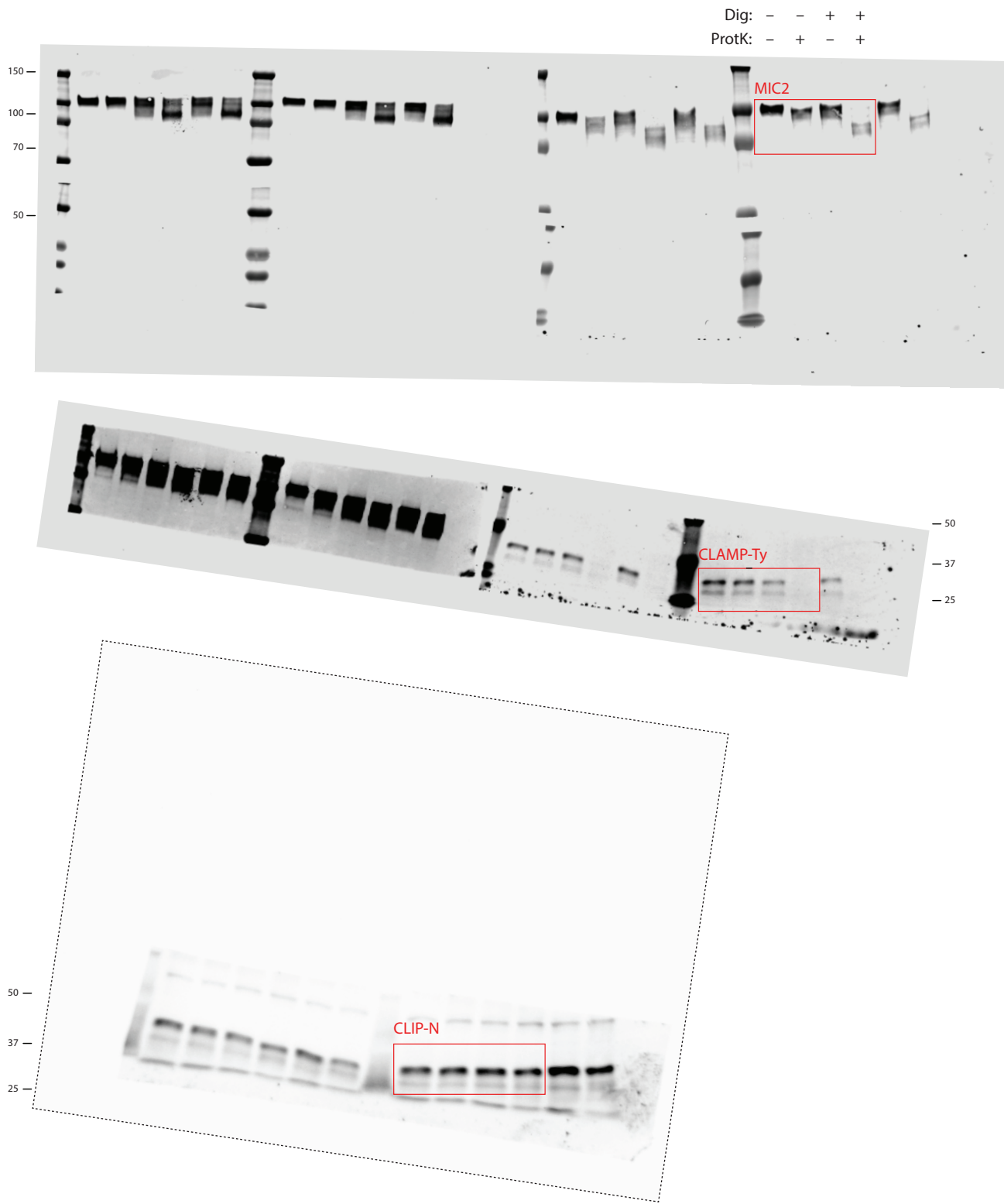

Supplement: Supplementary file 16 — Source Data for Figure 3 [file EMBJ-42-e113155-s017.zip › Figure_3/3G.pdf]

Figure 3I

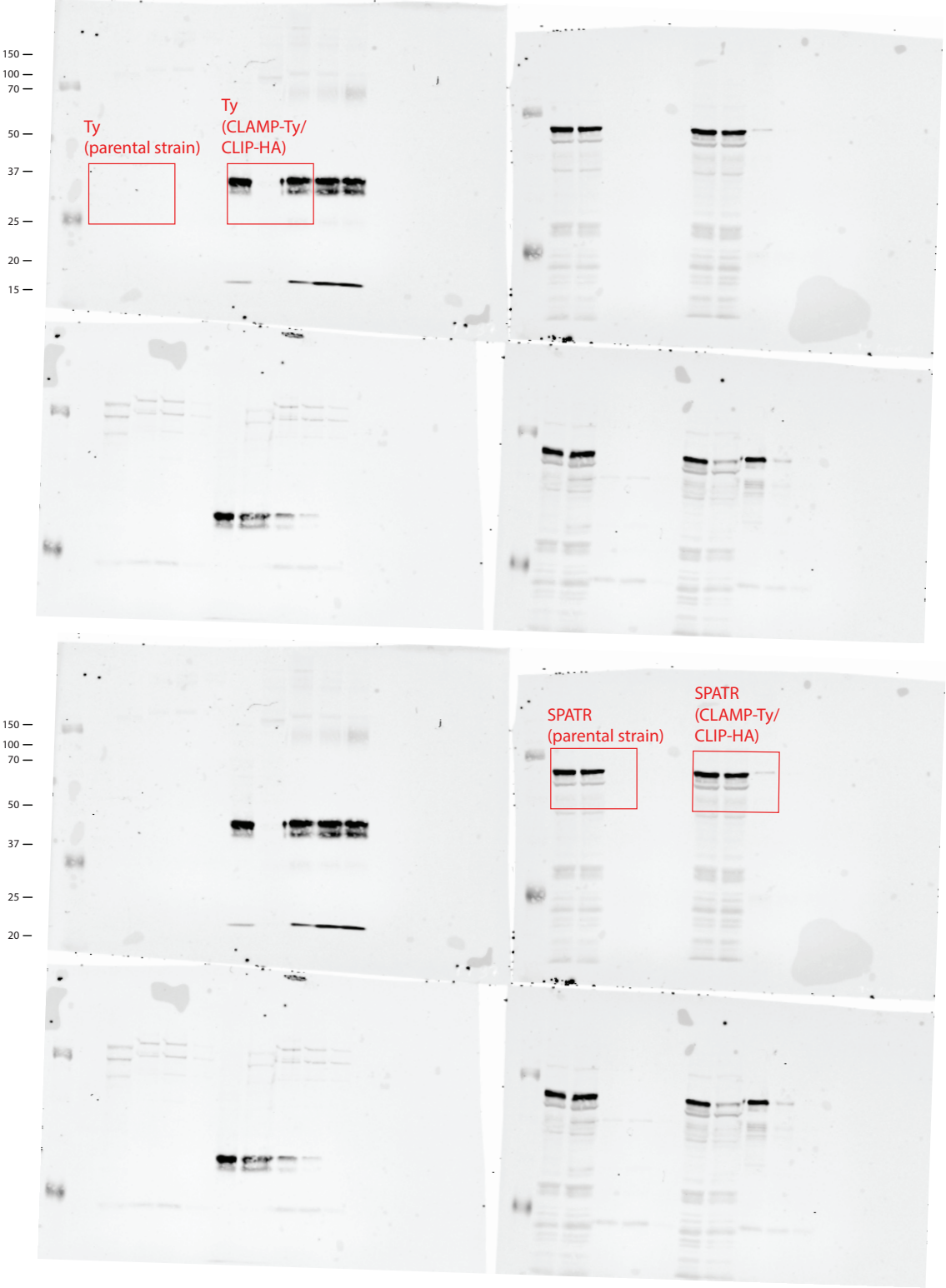

Figure 3I continued

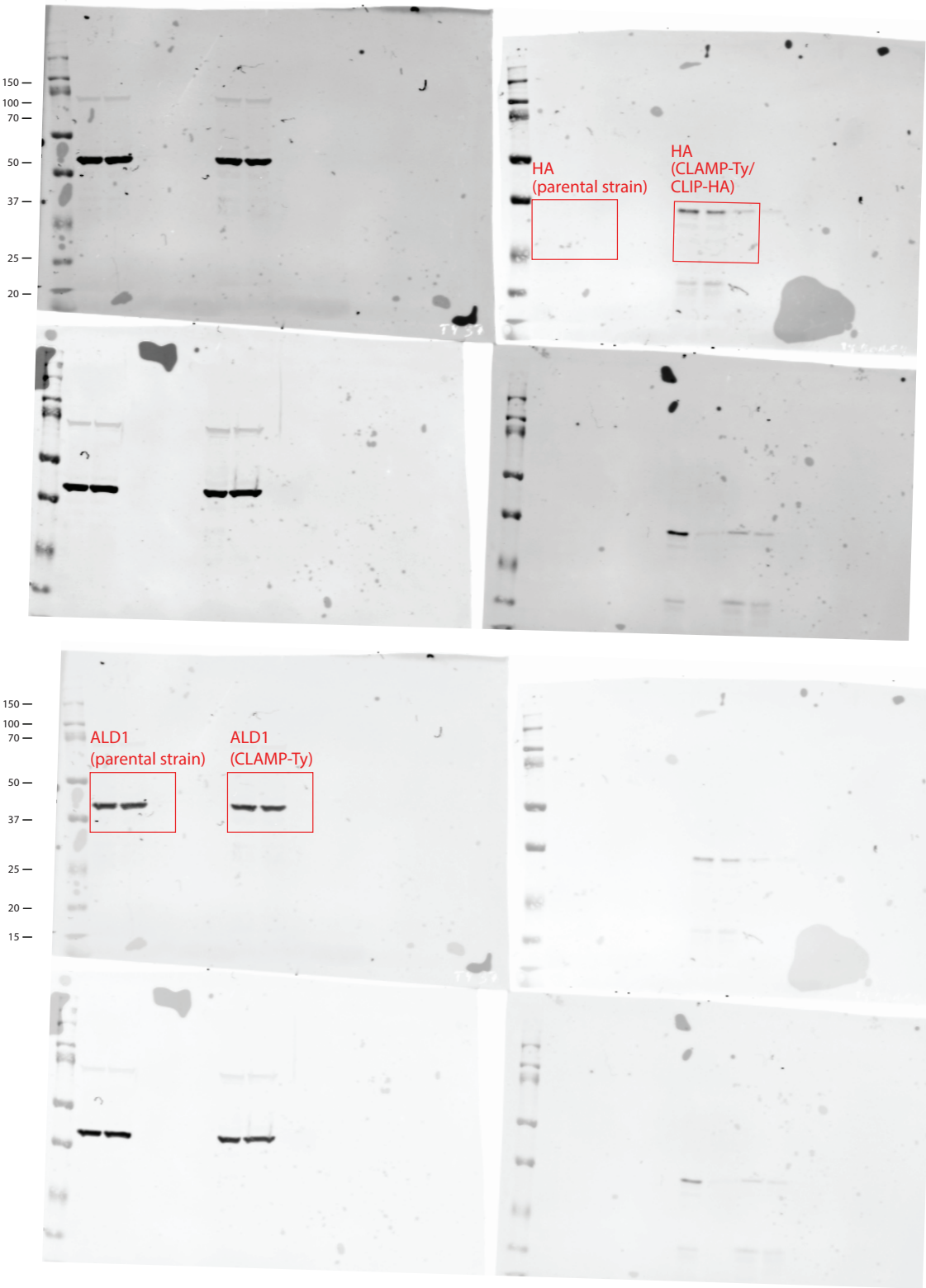

Supplement: Supplementary file 16 — Source Data for Figure 3 [file EMBJ-42-e113155-s017.zip › Figure_3/3I.pdf]

Figure 3J

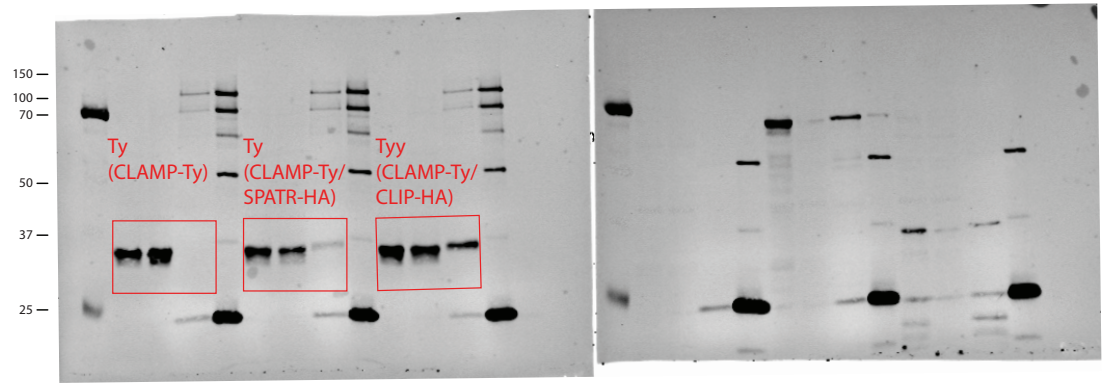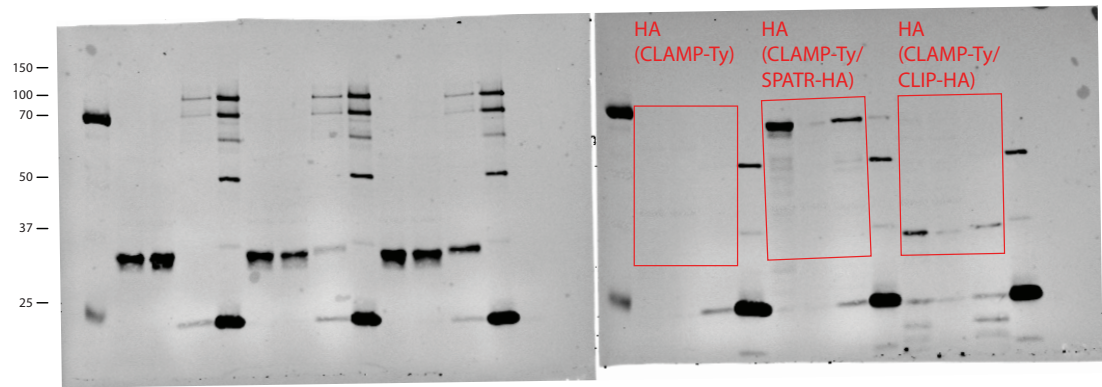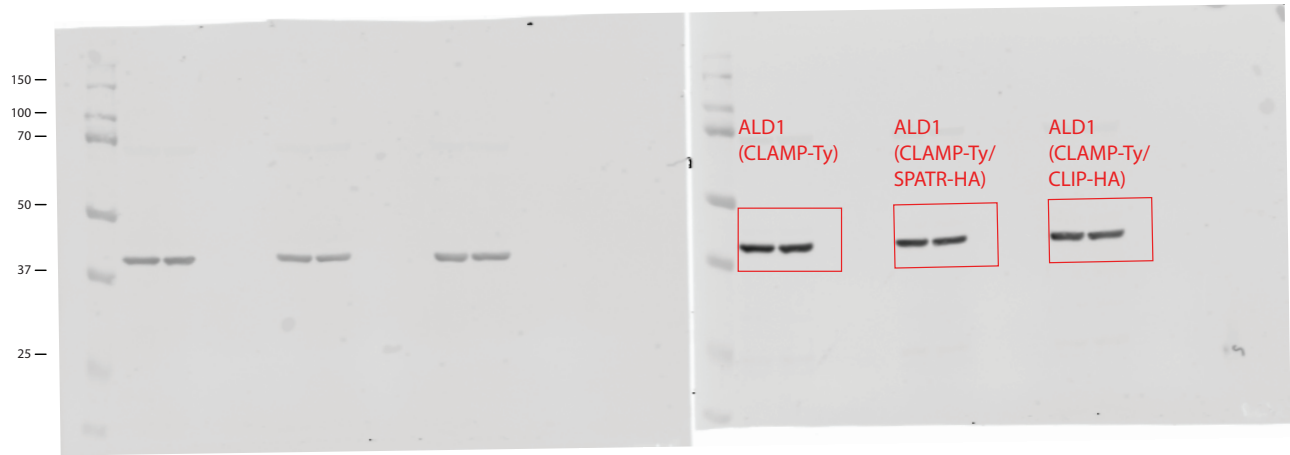

Supplement: Supplementary file 16 — Source Data for Figure 3 [file EMBJ-42-e113155-s017.zip › Figure_3/3J.pdf]

Figure 4B

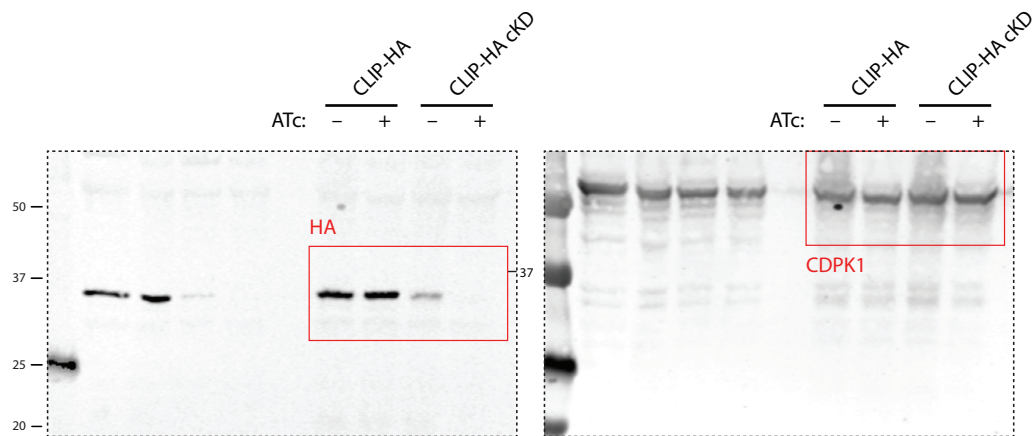

Supplement: Supplementary file 17 — Source Data for Figure 4 [file EMBJ-42-e113155-s005.zip › Figure_4/4B.pdf]

Figure 5H

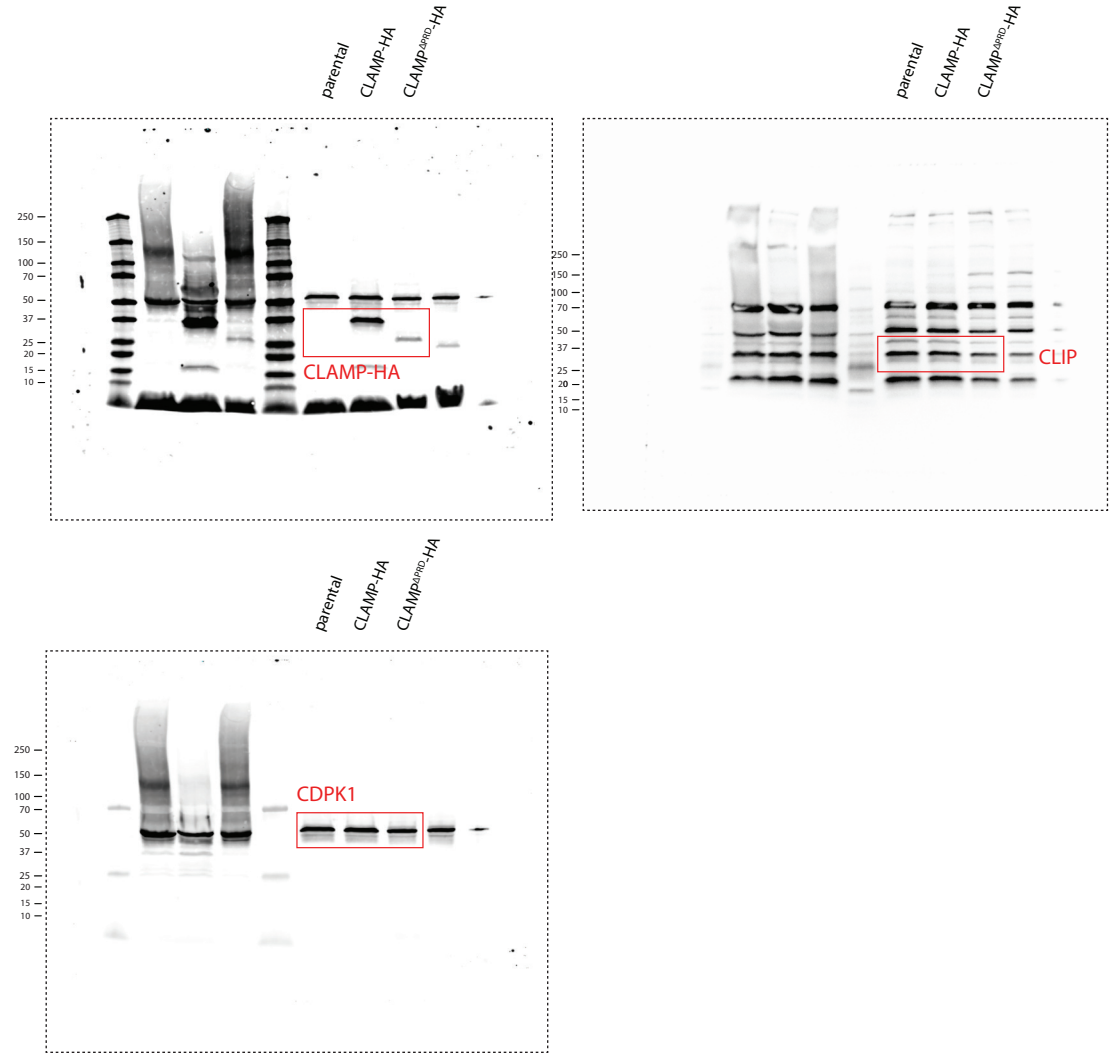

Supplement: Supplementary file 18 — Source Data for Figure 5 [file EMBJ-42-e113155-s001.zip › Figure_5/5H.pdf]
