# Supplementary material for: A conserved complex of microneme proteins mediates rhoptry discharge in Toxoplasma
Source: EMBO J. 2023 Oct 27;42(23):e113155. doi: 10.15252/embj.2022113155 (PMC10690463; doi:10.15252/embj.2022113155)
Supplement: Supplementary file 20 [file EMBJ-42-e113155-s006.pdf]

THE EMBO JOURNAL

A conserved complex of microneme proteins mediates rhoptry discharge in Toxoplasma

Dylan Valleau, Saima Sidik, Luiz Godoy, Yamilex Acevedo-Sánchez, Charisse Flerida Pasaje, My-Hang Huynh, Vern B. Carruthers, Jacquin Niles, and Sebastian Lourido
DOI: 10.15252/embj.2022113155

Corresponding author: Sebastian Lourido (lourido@wi.mit.edu)

Review Timeline:

Submission Date:	27th Nov 22
Editorial Decision:	2nd Jan 23
Revision Received:	19th Jul 23
Editorial Decision:	22nd Aug 23
Revision Received:	19th Sep 23
Accepted:	25th Sep 23

Editor: Kelly Anderson

Transaction Report:

(Note: With the exception of the correction of typographical or spelling errors that could be a source of ambiguity, letters and reports are not edited. Depending on transfer agreements, referee reports obtained elsewhere may or may not be included in this compilation. Referee reports are anonymous unless the Referee chooses to sign their reports.)

Dear Prof. Lourido,

Thank you for submitting your manuscript for consideration by the EMBO Journal. It has now been seen by three referees whose comments are shown below.

As you will notice, there is some question regarding whether the findings are sufficiently novel for The EMBO Journal, raised mainly by Referee 3 with Referee 2 also mentioning a lack of new mechanistic insight. To overcome the novelty concerns, please focus your revision on providing some mechanistic understanding of how these factors influence rhoptry discharge.

Should you be able to address these criticisms in full, we could consider a revised manuscript. I should remind you that it is EMBO Journal policy to allow a single round of revision only and that, therefore, acceptance or rejection of the manuscript will depend on the completeness of your responses in this revised version. It would be good to discuss your plan to address the referee concerns and I am available to do so via email or zoom in the coming weeks.

I have attached a guide for revisions for your convenience. Please bear in mind that all decision letters, referee comments, and your point-by-point response will form part of the Review Process File and will therefore be available online to the community. For more details on our Transparent Editorial Process, please visit our website: https://www.embo.org/embo-press

We generally allow three months as standard revision time. As a matter of policy, competing manuscripts published during this period will not negatively impact on our assessment of the conceptual advance presented by your study. However, we request that you contact the editor as soon as possible upon publication of any related work, to discuss how to proceed. Should you foresee a problem in meeting this three-month deadline, please let us know in advance and we may be able to grant an extension.

Thank you for the opportunity to consider your work for publication. I look forward to your revision.

Yours sincerely,

Kelly M Anderson, PhD
Editor
The EMBO Journal
k.anderson@embojournal.org

Further information is available in our Guide For Authors: https://www.embopress.org/page/journal/14602075/authorguide

The revision must be submitted online within 90 days; please click on the link below to submit the revision online before 2nd Apr 2023.

Referee #1:

Sidik et al. report on the function of the microneme protein CLAMP and its interactions with the proteins SPATR and a newly characterised protein CLIP. They find that the members of this protein complex have important functions during Toxoplasma growth and invasion into host cells. This complex is identified to have a role in discharge of proteins from the parasites rhoptry organelles. The manuscript is well written and clear, interpretation of data is fair and new insights into parasite invasion biology are described. The data is high quality and the MS-ESA technique developed is a useful addition to the field.

Major comments.
-Figure 2C. The CLAMO cKD plus Rapamycin image is difficult to interprete with the parasites appearing to be rounded up compared to others and the nucleus hard to discern such that its not totally clear which end is the apical tip. In the lower parasite, it also appears that green staining surrounds the parasite with an intensity at least as strong as the e-vacuoles highlighted in the control plus Rapamycin. Is a different example image available or can the end with apical tip be highlight? Does the green surrounding the lower parasite indicate that rhoptry discharge has occurred in this parasite and it is highlighting the parasitophorous vacuole?
-The authors state that: 'Comparing the abundance ratios of proteins in the ESA fraction and whole-parasite lysates suggests

that CLAMP knockdown leads to decreased TGGT1_212270 stability, whereas the reduction of SPATR in the ESA fraction is likely due to a secretion or trafficking defect (Fig. 2H). Prior studies suggest that SPATR and TGGT1_212270 localize to micronemes where CLAMP could influence their trafficking and stability (Barylyuk et al, 2020; Sidik et al, 2016b; Huynh et al, 2014).'

To check whether there is a secretion or trafficking defect of SPATR, it should be relatively simple to tag this protein using the existing construct on the CLAMP cKO line and image what happens to SPATR localisation with CLAMP knock-down. TGGT1_212270 stability would be harder to quantify, but possible using the same approach.

-The authors state that: 'We conclude that CLAMP and CLIP most likely form a membrane-embedded complex, with the CLIP ectodomain, CLAMP loops 1 and 3, and SPATR exposed on the parasite surface after secretion from micronemes.'

As far as I can see, there is no compelling evidence in this study that suggests CLAMP loops 1 and 3 are involved in binding CLIP or SPATR. If evidence is available from previous studies, then this needs to be summarised and incorporated for this statement to be made. If there is no evidence, then this needs to be framed as a speculation and the reasons for this described.

-Figure 4E. The CDPK1 loading control indicates that protein loading on the gel is relatively constant. The CLIP-HA cKD line without Rapamycin has significantly less protein that the CLIP-HA without Rapamycin. Does this indicate that addition of the CLIP-HA cKD plasmid is reducing expression of the gene even in the absence o Rapamycin? Can this be tested using additional gels and quantifying any potential protein expression loss? There appears to be no impact on growth for CLIP-HA cKD in 4F & G, but it may that the parasites can tolerate some protein loss before a growth defect is evident, which would be of interest.

Minor Comments

-In the results the authors state and cite:

'show peaks of expression during the chronic stages (TGGT1_212275) or in the definitive host (TGGT1_219742) (Ramakrishnan et al, 2019).'

In the discussion, the authors state and cite:

'TGGT1_212275 is upregulated in bradyzoites (Waldman et al, 2020). TGGT1_219742 is expressed at very low levels throughout the asexual lifecycle but appears to be upregulated in merozoites within the definitive host (Waldman et al, 2020; Hehl et al, 2015; Ramakrishnan et al, 2019).

It seems to me there may be some references that could represent the data earlier in the results?

-Please state the number of repeat experiments for the P. falciparum growth assays.

Referee #2:

This manuscript describes an expansion of previous work on the micronemal protein CLAMP.

CLAMP is a micronemal protein previously determined to be needed for invasion. In hthis new work they show that this defect is likely due to a defect in rhoptry secretion, which is consistent with the known role of certain micronemal proteins in rhoptry secretion. Furthermore, the identified two interactors whose secretion and/or stability depends on CLAMP. They call one of these CLIP and show that it is also needed for invasion. The data presented is strong and well-controlled for. While the work does not add new mechanistic information, it does expand on our understanding of rhoptry secretion. As they include data on the Plasmodium homologs of CLAMP and its interactor SPATR, the work has broad impact. Some specific items that could be address before publication include:

For figure 1C, in the absence of digitonin there is a shift in the relative signals of MIC2 and CLAMP upon ProK treatment. What explains this result?

For figure 2, previous work showed that CLAMP is needed for invasion. Since they generated a new knockdown for the work presented in this manuscript, the invasion phenotype should be confirmed and the results shown in the supplemental figure. This is important for the interpretation of the phenotypic assays presented in figure 2.

In figure 3C, the eluted CLAMP1 appears to migrate differently than the one in the input. Why would that be? Interestingly the same thing is seen in figure 3 F.

A model figure of the interactions and when and where they occur could be of help.

Some minor issues include:

Since figure 4 is so busy and the TET system is fairly standard there might not be a need for 4D.

Do structure predictions provide any hints about how these proteins might interact?

In the discussion, in the passage "and show broad conservation across apicomplexans (Mageswaran et al, 2021; Aquilini et al, 2021; Sparvoli et al, 2022) By contrast, orthologs of MIC8 are limited to T. gondii and its closest" a period is missing.

Referee #3:

This work by Sidik and Valleau et al describes a new protein complex involved in invasion in Toxoplasma with evidence that this might also be important for the wider apicomplexan phylum. In previous work the Lourido lab (Sidik et al 2016, Cell PMID: 27594426) has previously described CLAMP - a multipass transmembrane domain protein that was isolated as a conserved, highly fitness conferring gene, that is important for Toxoplasma invasion, but not egress or motility. They also demonstrated that CLAMP follows the moving junction, further implicating this protein in invasion.

The authors extend this work here and show that CLAMP is required for secretion of the rhoptry organelles and use mass spectrometry to confirm that CLAMP is not required for microneme secretion and demonstrate it complexes with at least 2 other proteins. The authors provide strong evidence that CLAMP complexes with a previously partially characterized protein SPATR and a newly identified protein CLIP. They show that loss of CLIP has a similar phenotype to CLAMP, which is somewhat different to SPATR, which was previously published not to have such a strong phenotype (Huynh et al 2014 Inf Imm PMID: 25092910). They then go on to confirm that CLAMP is required for asexual growth of P. falciparum and that SPATR too is important. They do not however, show that these proteins are required for invasion in P. falciparum, although that is most likely.

This paper is nicely put together, is clear and concise and the data robust. My main issue that I have with this manuscript is novelty. This lab has already identified CLAMP as an important factor required for invasion (but not egress or motility) (Sidik et al 2016, Cell), and they also showed that this protein also plays a role in P. falciparum growth. SPATR has previously been characterised (Huynh et al 2014) but CLIP is new. The fact that they form a complex is also new. Loss of CLIP and CLAMP result in a defect in rhoptry discharge, however, this is not novel phenotype. MIC8 and a recently described CRMP complex also have the same phenotype and thus the characterisation of this complex does not shed any new light on the invasion in any novel way.

This works discoveries are not as broad and/or novel as I would expect to see published in EMBO J. I feel like this manuscript is more of a confirmation of their findings that they published in Cell and a follow up that the protein resides as a complex.

An interesting comparator in this regard in Sparvoli et al, which was recently published in EMBO J. They also describe a new protein complex involved in invasion and provide evidence that it is involved in rhoptry discharge. Their work goes much further however, as this complex has an interesting and novel localisation at the very tip of the parasite and the authors provide compelling evidence of its evolutionary origins which has profound implications in understanding how rhoptries and more generally invasion evolved in this group of parasites.

Other points:

Its important that the introduction and discussion include discussion on how other apicomplexan parasites recognise their host cells. Or at least point to a review that covers this area and state that you only talking about proteins and complexes that are conserved across the phylum. There is a lot known about P. falciparum invasion in particular.

I see little value in the electron micrographs provided in figure 2B. There is no clear phenotype, by the authors admission, and thus I don't think they should be included.

Why SPATR has an apparent milder phenotype than loss of CLAMP and CLIP is interesting and worth more explicitly talking about in the discussion.

RESPONSE TO REVIEWERS

Referee #1:

Sidik et al. report on the function of the microneme protein CLAMP and its interactions with the proteins SPATR and a newly characterized protein CLIP. They find that the members of this protein complex have important functions during Toxoplasma growth and invasion into host cells. This complex is identified to have a role in discharge of proteins from the parasites rhoptry organelles. The manuscript is well written and clear, interpretation of data is fair and new insights into parasite invasion biology are described. The data is high quality and the MS-ESA technique developed is a useful addition to the field.

Major comments.

-Figure 2C. The CLAMP cKD plus Rapamycin image is difficult to interpret with the parasites appearing to be rounded up compared to others and the nucleus hard to discern such that its not totally clear which end is the apical tip. In the lower parasite, it also appears that green staining surrounds the parasite with an intensity at least as strong as the e-vacuoles highlighted in the control plus Rapamycin. Is a different example image available or can the end with apical tip be highlight? Does the green surrounding the lower parasite indicate that rhoptry discharge has occurred in this parasite and it is highlighting the parasitophorous vacuole?

The evacuoles were counted by epifluorescence, and because of the low relative intensity of evacuoles compared to the rhoptry contents in the parasites, a high exposure and light intensity was required to detect the evacuoles. Under these conditions, the signal surrounding the parasites is bleed-through from the internal ROP1, and does not indicate a discharge event. To the reviewer's second point, we performed this assay in presence of the actin inhibitor cytocholasin D, which blocks parasite invasion and formation of the parasitophorous vacuole. To avoid confusion, we collected some representative images by confocal microscopy, leading to better resolution and reduced background from the internal ROP1 (now displayed in **Fig. 2C**). Since quantification was performed with the epifluorescence images, we have retained these additional example images in **Fig. S2B**, and noted this in the materials and methods.

-The authors state that: 'Comparing the abundance ratios of proteins in the ESA fraction and whole-parasite lysates suggests that CLAMP knockdown leads to decreased TGGT1_212270 stability, whereas the reduction of SPATR in the ESA fraction is likely due to a secretion or trafficking defect (Fig. 2H). Prior studies suggest that SPATR and TGGT1_212270 localize to micronemes where CLAMP could influence their trafficking and stability (Barylyuk et al, 2020; Sidik et al, 2016b; Huynh et al, 2014).'

To check whether there is a secretion or trafficking defect of SPATR, it should be relatively simple to tag this protein using the existing construct on the CLAMP cKO line and image what happens to SPATR

localisation with CLAMP knock-down. TGGT1_212270 stability would be harder to quantify, but possible using the same approach.

We agree that confirming whether there is a secretion and/or trafficking defect of SPATR is a valuable addition to this manuscript. These experiments can now be found in **Fig. 5I–J** and **S5A–D**. We also performed SPATR co-localization with antibodies raised against CLIP (TGGT1_212270), MIC3, proM2AP, and NHE3 during CLAMP knockdown. These new experiments confirm that the localization of other microneme proteins (e.g. MIC3) was unaltered, while SPATR was largely depleted from micronemes (**Fig. 5J**) and the remaining signal was internal. In the absence of CLAMP, SPATR did not colocalize with an endosomal marker (NHE3) and only partially with the trans-Golgi marker proM2AP. Despite imperfect colocalization with the markers examined, these new results suggest that SPATR is mislocalized upon CLAMP knockdown, rather than there being merely destabilized. By contrast, CLAMP depletion leads to the degradation of CLIP (**Fig. 5I**), consistent with MS results.

-The authors state that: 'We conclude that CLAMP and CLIP most likely form a membrane-embedded complex, with the CLIP ectodomain, CLAMP loops 1 and 3, and SPATR exposed on the parasite surface after secretion from micronemes.'

As far as I can see, there is no compelling evidence in this study that suggests CLAMP loops 1 and 3 are involved in binding CLIP or SPATR. If evidence is available from previous studies, then this needs to be summarized and incorporated for this statement to be made. If there is no evidence, then this needs to be framed as a speculation and the reasons for this described.

We have amended the text to indicate that the topology of the CLAMP loops remains speculative, despite support from biochemical and computational predictions. Topology predictions were confirmed by proteinase K protection and membrane association studies. New data from a strain that swaps wild-type CLAMP with a version lacking the C-terminal cytosolic proline-rich domain shows that the C terminus is not required for complex formation, as predicted by our model. Furthermore, we used AlphaFold Multimer to predict the structure of this complex, which aligns the transmembrane helices of CLIP and CLAMP, and orients the CLAMP loops 1 and 3 to form a structure that binds CLIP's C-terminal region while CLIP's Nv terminus binds SPATR (**Fig. 3K**). Notably, the region predicted to bind CLAMP is the most conserved portion of CLIP (**Fig. S3C**) and the *Plasmodium falciparum* CLAMP, CLIP, and SPATR adopt the same configuration in AlphaFold predictions (**Fig. S3D–E**). We agree with the reviewer's assessment that further work will be required to experimentally validate the model of the complex.

-Figure 4E. The CDPK1 loading control indicates that protein loading on the gel is relatively constant. The CLIP-HA cKD line without Rapamycin has significantly less protein that the CLIP-HA without Rapamycin. Does this indicate that addition of the CLIP-HA cKD plasmid is reducing expression of the gene even in the absence of Rapamycin? Can this be tested using additional gels and quantifying any potential protein expression loss? There appears to be no impact on growth for CLIP-HA cKD in 4F & G, but it may that the parasites can tolerate some protein loss before a growth defect is evident, which would be of interest.

Replacement of the endogenous CLIP promoter with TetO7-SAG1 indeed lowers the overall expression of CLIP-HA, which we consistently observed, even in media containing ATc-free FBS. We quantified this reduction in expression over three replicates (**Fig. S4D**) and quantified the CDPK1-normalized expression of CLIP-HA, which found a 62% reduction in CLIP-HA even in absence of ATc knockdown (**Fig. S4C**). We expect this is a common effect of using the Tet-knockdown system in general; however, this comparison is not always performed by other studies using the system. As noted by the reviewer, there is no impact on growth or invasion despite the lowered level of CLIP-HA, suggesting some protein loss is tolerated under the conditions tested. As suggested, we have updated the results section to describe this noteworthy detail.

Minor Comments

-In the results the authors state and cite:

'show peaks of expression during the chronic stages (TGGT1_212275) or in the definitive host (TGGT1_219742) (Ramakrishnan et al, 2019).'

In the discussion, the authors state and cite:

'TGGT1_212275 is upregulated in bradyzoites (Waldman et al, 2020). TGGT1_219742 is expressed at very low levels throughout the asexual lifecycle but appears to be upregulated in merozoites within the definitive host (Waldman et al, 2020; Hehl et al, 2015; Ramakrishnan et al, 2019).

It seems to me there may be some references that could represent the data earlier in the results?

Thank you to the reviewer for noticing this oversight. We have now updated the references in the results section that support these statements.

-Please state the number of repeat experiments for the P. falciparum growth assays.

We thank the reviewer for noticing this oversight. The growth assays (now in **Fig. 6B-E**) were all performed in triplicate (n = 3), which has now been noted in both the appropriate figure legend and the Materials and Methods.

Referee #2:

This manuscript describes an expansion of previous work on the micronemal protein CLAMP.

CLAMP is a micronemal protein previously determined to be needed for invasion. In this new work they show that this defect is likely due to a defect in rhoptry secretion, which is consistent with the known role of certain micronemal proteins in rhoptry secretion. Furthermore, the identified two interactors whose secretion and/or stability depends on CLAMP. They call one of these CLIP and show that it is also needed for invasion. The data presented is strong and well-controlled for. While the work does not add new mechanistic information, it does expand on our understanding of rhoptry secretion. As they

include data on the Plasmodium homologs of CLAMP and its interactor SPATR, the work has broad impact.

We would like to thank the reviewer for their detailed assessment of this work. In an ongoing effort to expand the broad impact of this manuscript, we have performed additional experiments in *P. falciparum* to more specifically show there is an RBC invasion defect when SPATR and CLAMP are knocked down, further demonstrating the functional importance of the complex. While we agree there are still many questions surrounding rhoptry discharge, we believe that identifying one of a few protein complexes that influence this process is an important step towards the broader goal of uncovering how the process works mechanistically.

Some specific items that could be address before publication include:

For figure 1C, in the absence of digitonin there is a shift in the relative signals of MIC2 and CLAMP upon ProK treatment. What explains this result?

The ProK protection assay performed here, and previously reported in the field, is often noisy due to the required TCA precipitation step, needed to inactivate ProK and concentrate proteins. We repeated this assay using a serine proteinase inhibitor (PMSF) instead of TCA precipitation, which allowed us to miniaturize the assay obviating protein concentration. As now seen in the revised **Fig. 1C**, the relative signals of MIC2 and CLAMP-Ty are more consistent between samples, while reaffirming the key result that the CLAMP C terminus is exposed to the cytosol. We also used this opportunity to examine the topology of CLIP by ProK protection (**Fig. 3G**), which we think is a valuable addition to the manuscript.

For figure 2, previous work showed that CLAMP is needed for invasion. Since they generated a new knockdown for the work presented in this manuscript, the invasion phenotype should be confirmed and the results shown in the supplemental figure. This is important for the interpretation of the phenotypic assays presented in figure 2.

We performed the requested experiment, which confirmed that rapamycin induced knockdown of CLAMP in the CLAMP-HA cKD leads to inhibition of invasion (**Fig. S1D**).

In figure 3C, the eluted CLAMP1 appears to migrate differently than the one in the input. Why would that be? Interestingly the same thing is seen in figure 3 F.

We thank the reviewer for pointing this out. The shift in migration appears to result from differences in buffer compositions between the immunoprecipitated samples and whole-cell lysates or flowthrough samples. Notably, in the reciprocal immunoprecipitations (now **Fig. 3I** and **3J**), we notice subtle shifts in migration for SPATR and CLIP-HA. We can attribute changes in migration to several possible factors, such as detergent to protein ratios, ion concentrations, or the carrier effect of co-migrating proteins in the lysate. Overall, we think that this is a general effect from SDS-PAGE that does not impact the conclusions of the manuscript.

A model figure of the interactions and when and where they occur could be of help.

We agree that a schematic of the assembled complex in micronemes and on the parasite surface is a useful addition that was overlooked in the initial submission. A model schematic of these proteins can be found in **Fig. 3H**, which replaces the CLIP-specific model we had previously included in **Fig. 4**.

Some minor issues include:

Since figure 4 is so busy and the TET system is fairly standard there might not be a need for 4D.

We certainly appreciate trying to keep the figures straightforward. We feel it makes the manuscript more accessible to include the diagrams since we use a Tet-based system with the opposite ATc response (i.e., one is ATc-ON, while the other is ATc-OFF) to regulate gene expression in *P. falciparum* (Figure 6).

Do structure predictions provide any hints about how these proteins might interact?

We appreciate the helpful suggestion. We were able to use AlphaFold to predict a model for the CLAMP, CLIP, and SPATR complex (**Fig. 3K**), which is consistent with the biochemical results and topology predictions summarized in **Fig. 3H**. Notably, the C-terminal TM of CLIP bundles with the 4 TM helices of CLAMP in our model, and clear CLAMP-CLIP and CLIP-SPATR interfaces coincide with the most conserved regions in each protein. The CLIP interface with CLAMP consisted of the most conserved stretches of CLIP which interacted with the CLAMP extracellular loops. Despite lower conservation of motifs in the N-terminal region CLIP, where CLIP is modeled to contact SPATR, the interfaces appeared conserved in AlphaFold models of the *P. falciparum* complex. In support of this model, we have added an experiment showing that a version of CLAMP lacking the cytosolic proline-rich domain ($CLAMP^{\Delta PRD}$) still interacts with CLIP and SPATR (**Fig. 5I**). The PRD was predicted to be unstructured in our AlphaFold structure and had no contacts with the rest of the model. These models and more details are now described in the manuscript and presented in **Fig. 3K** and **Fig. S3C–E**.

In the discussion, in the passage "and show broad conservation across apicomplexans (Mageswaran et al, 2021; Aquilini et al, 2021; Sparvoli et al, 2022) By contrast, orthologs of MIC8 are limited to T. gondii and its closest" a period is missing.

We have fixed this typo.

Referee #3:

This work by Sidik and Valleau et al describes a new protein complex involved in invasion in Toxoplasma with evidence that this might also be important for the wider apicomplexan phylum. In previous work the Lourido lab (Sidik et al 2016, Cell PMID: 27594426) has previously described CLAMP - a multipass transmembrane domain protein that was isolated as a conserved, highly fitness conferring gene, that is

important for Toxoplasma invasion, but not egress or motility. They also demonstrated that CLAMP follows the moving junction, further implicating this protein in invasion.

The authors extend this work here and show that CLAMP is required for secretion of the rhoptry organelles and use mass spectrometry to confirm that CLAMP is not required for microneme secretion and demonstrate it complexes with at least 2 other proteins. The authors provide strong evidence that CLAMP complexes with a previously partially characterized protein SPATR and a newly identified protein CLIP. They show that loss of CLIP has a similar phenotype to CLAMP, which is somewhat different to SPATR, which was previously published not to have such a strong phenotype (Huynh et al 2014 Inf Imm PMID: 25092910). They then go on to confirm that CLAMP is required for asexual growth of P. falciparum and that SPATR too is important. They do not however, show that these proteins are required for invasion in P. falciparum, although that is most likely.

We agree that further assessing the role of the CLAMP complex in *P. falciparum* is important. We have therefore performed additional experiments to determine which stage of the *P. falciparum* lytic cycle is impacted by loss of *Pf*CLAMP or *Pf*SPATR. Using synchronized blood-stage cultures, we found that the parasites replicate normally, formed schizonts, and egressed; however, the number of rings generated during the next round of invasion is significantly lowered when *Pf*CLAMP or *Pf*SPATR is knocked down (**Fig. 6F-H**, Supplemental **Videos 3-6**). These results implicate *Pf*CLAMP and *Pf*SPATR in invasion of erythrocytes, presumably due to a defect in rhoptry secretion.

This paper is nicely put together, is clear and concise and the data robust. My main issue that I have with this manuscript is novelty. This lab has already identified CLAMP as an important factor required for invasion (but not egress or motility) (Sidik et al 2016, Cell), and they also showed that this protein also plays a role in P. falciparum growth. SPATR has previously been characterised (Huynh et al 2014) but CLIP is new. The fact that they form a complex is also new. Loss of CLIP and CLAMP result in a defect in rhoptry discharge, however, this is not novel phenotype. MIC8 and a recently described CRMP complex also have the same phenotype and thus the characterisation of this complex does not shed any new light on the invasion in any novel way.

This works discoveries are not as broad and/or novel as I would expect to see published in EMBO J. I feel like this manuscript is more of a confirmation of their findings that they published in Cell and a follow up that the protein resides as a complex.

An interesting comparator in this regard in Sparvoli et al, which was recently published in EMBO J. They also describe a new protein complex involved in invasion and provide evidence that it is involved in rhoptry discharge. Their work goes much further however, as this complex has an interesting and novel localisation at the very tip of the parasite and the authors provide compelling evidence of its evolutionary origins which has profound implications in understanding how rhoptries and more generally invasion evolved in this group of parasites.

We appreciate the reviewer's comments regarding the quality and presentation of our data. We understand that the effects of CLAMP and SPATR in invasion were previously described by our lab and others; however, we firmly believe that there is significant novelty with the integration of these observations under a unified model that includes complex formation and a specific role in rhoptry discharge. Though other proteins (i.e. MIC8 and CRMPs) have been shown to play similar roles in rhoptry discharge, we do not believe that diminishes the novelty of our work. Instead, we argue that it is critically important to identify all of the conserved proteins that participate at this essential step in the parasite life cycle because it is not the mere existence of such proteins that matters but rather their precise identity and how they come together. From that perspective we believe our study is as novel as that of Sparvoli et al. and helps set the stage for future research integrating the various protein complexes into a unified mechanistic model of rhoptry discharge.

To expand on the Sparvoli et al. study specifically, since both the CRMP and CLAMP complexes are required for rhoptry secretion and track with the moving junction, their functions are potentially coordinated during rhoptry secretion and invasion. While CRMPs are conserved beyond apicomplexans and function in the secretion apparatuses of free-living ciliates, CLAMP and CLIP appear to specify these functions in apicomplexans. In the revised manuscript, we have further explored the interaction between components of the CLAMP complex, functionally separating, in the case of CLAMP, complex formation from its function triggering rhoptry discharge (**Fig. 5G-J**). It is established that host-cell contact is required for rhoptry discharge but, to our knowledge, this is the first evidence that a specific protein complex is responsible for transmitting a required signal across the parasite plasma membrane. Together with the newly established importance in *P. falciparum*, our work argues for the broad importance of the CLAMP complex in apicomplexans. We hope the reviewer will appreciate the mechanistic importance of these discoveries even if, as a field, we still lack a unified model for rhoptry discharge. We hope the reviewer will appreciate the mechanistic importance of these discoveries even if, as a field, we still lack a unified model for rhoptry discharge.

Other points:

Its important that the introduction and discussion include discussion on how other apicomplexan parasites recognise their host cells. Or at least point to a review that covers this area and state that you only talking about proteins and complexes that are conserved across the phylum. There is a lot known about *P. falciparum* invasion in particular.

We appreciate the extensive literature on host cell recognition by *P. falciparum*. While we have focused on the factors relevant to rhoptry secretion in *T. gondii*, we have added additional information to the introduction and discussion addressing what is known about rhoptry secretion in *P. falciparum* and pointing to a review in the introduction with more details on the various components of apicomplexan invasion machinery (Cova *et al.*, 2022).

I see little value in the electron micrographs provided in figure 2B. There is no clear phenotype, by the authors admission, and thus I don't think they should be included.

Seeing parasites adhering apically and establishing a close apposition of their plasma membrane with that of the host helps us characterize the role of CLAMP in the interaction with host cells. The images in **Fig. 2B** show that CLAMP-deficient parasites form a close apposition between their apical ends and the host plasma membrane, but that apposition does not progress towards a more comprehensive interaction. This supports our conclusion that rhoptry discharge is specifically defective in the absence of CLAMP, rather than the defect originating from global changes in cell adhesion. For these reasons, we have opted to retain the images in the current version of the manuscript, and hope the reviewer understands our reasoning for this choice.

Why SPATR has an apparent milder phenotype than loss of CLAMP and CLIP is interesting and worth more explicitly talking about in the discussion.

We agree that the milder phenotype of SPATR is interesting, and have acted on the reviewer's suggestion to expound on the potential causes and mechanistic implications of this result in the manuscript. Specifically, we discuss how recent work has found that SPATR is more important during mouse infection relative to cell culture (Giuliano, *et al.* 2023), implying that the permissive environment of cell culture is partly responsible for its dispensability. Additionally, in the predicted model of the complex (**Fig. 3K**), SPATR interfaces with CLIP's less conserved N-terminal region, which is consistent with SPATR's lowered conservation amongst apicomplexans relative to CLIP and CLAMP. SPATR may therefore be an accessory component of the complex, enhancing the activity of the essential components and dispensable in certain contexts. We have amended the text to highlight these points in the discussion.

1st Revision - Editorial Decision 22nd Aug 2023

Dear Sebastian,

Congratulations on a great revision! Overall the referees have been positive, however referee 3 asks for quantification of Pf egress and invasion to address their concerns. While we realize this would require extra work and add a few more weeks before final publication, we feel this will greatly strengthen your important study.

When you submit your revised version, please also take care of the following editorial items and add this also to your point-by-point response:

1. Please add up to five keywords, which may or may not appear in the title, should be given in alphabetical order, below the abstract, each separated by a slash (/).

2. Please review our new policy on conflict of interests on the EMBO author guide website and update the title of this section to: Disclosure and competing interests statement

3. Please remove the author contributions section from the main manuscript.

4. Please upload EV figures as figure files and not expanded view files.

5. For dataset EV1-3 and Table EV1 should be unzipped and the legend should be provided as an additional sheet in the excel file. Typically Tables are not in excel format, if you'd like to keep it in Excel, please refer to it as a dataset.

6. We require the publication of source data, particularly for electrophoretic gels and blots and graphs, with the aim of making primary data more accessible and transparent to the reader. It would be great if you could provide me with a PDF file per figure that contains the original, uncropped and unprocessed scans of all or key gels used in the figure or for graphs, an Excel spreadsheet with the original data used to generate the graphs. The PDF files should be labeled with the appropriate figure/panel number, and should have molecular weight marker; further annotation could be useful but is not essential. The PDF files will be published online with the article as supplementary "Source Data" files.

7. We include a synopsis of the paper (see http://emboj.embopress.org/). Please provide me with a general summary statement and 3-5 bullet points that capture the key findings of the paper.

8. We also need a summary figure for the synopsis. The size should be 550 wide by 200-440 high (pixels). You can also use something from the figures if that is easier.

9. Our publisher has also done their pre-publication check on your manuscript. I have attached the "Data Edited Manuscript file". Please take a look at the word file and the comments regarding the figure legends and respond to the issues. In particular, we generally don't allow statistics on N=2, rather this should be displayed as individual data points.

10. Please correct the order of sections to: abstract, introduction, results, discussion, materials & methods, data availability section, acknowledgments, disclosure statement and competing interests, references, main figure legends, tables, expanded figure legends.

Thank you for the opportunity to consider your work for publication. I look forward to your revision.

Kind regards,

Kelly

Kelly M Anderson, PhD
Editor, The EMBO Journal
k.anderson@embojournal.org

Further information is available in our Guide For Authors: https://www.embopress.org/page/journal/14602075/authorguide

Use the link below to submit your revision:

Referee #1:

The authors have addressed my comments and provide new data on P. falciparum invasion and structure predictions. These data support the authors conclusions on broad apicomplexan function and structure of these proteins in complex. I believe the manuscript is significantly improved, provides novel insights into a protein complex needed for parasites invasion and describes adequately described the function of conserved proteins required for invasion. I would suggest that the supplementary videos of P. falciparum rupture are cropped and shortened to focus on a shorter timescale around invasion and on the specific parasite rupturing. This would make it easier to identify and follow the cell of interest. Otherwise, this is a good manuscript and provides information of importance to the field of apicomplexan biology.

Referee #2:

The authors did an excellent job addressing the comments of the reviewers. The added data, model figures and edits to the manuscript make the document more complete and impactful.

Referee #3:

The authors have provided additional data to support their findings. Some limitations that I previously highlighted remain:

Novelty: There is no new data that supports a drastic increase in novelty. As previously stated, the impact of this paper is taken away by previous work by the same group and others. The identity of CLIP is novel and the fact that the complex is made of at least 3 components is also. This is the third protein complex that has been identified to be required for rhoptry release. The authors do provide data to suggest that it might be the Proline Rich Domain (PRD), of CLAMP might be important for signaling (but not complex formation) into the cell upon complex engagement. This, as they state, could be analogous to how claudin transduce signal through the membrane is not required for complex formation but it needed for rhoptry release. This does give additional insight on mechanism. I see that novelty has not been brought up by the other referees, so perhaps my views do not represent the greater community.

P. falciparum data: More data has been provided that make it more likely that the phenotype is what they think it is, without directly showing this. No definitive results can be deduced from one video, containing one egressing schizont, in each condition. This is not at standard for the field. There are widely available protocols to look at Pf egress and invasion quantitatively (eg see Blackmans work). It remains possible, yet unlikely given the work in Toxo, that loss of SPATR and CLAMP cause defects in parasite replication.

Referee #1:

The authors have addressed my comments and provide new data on P. falciparum invasion and structure predictions. These data support the authors conclusions on broad apicomplexan function and structure of these proteins in complex. I believe the manuscript is significantly improved, provides novel insights into a protein complex needed for parasites invasion and describes adequately described the function of conserved proteins required for invasion. I would suggest that the supplementary videos of P. falciparum rupture are cropped and shortened to focus on a shorter timescale around invasion and on the specific parasite rupturing. This would make it easier to identify and follow the cell of interest. Otherwise, this is a good manuscript and provides information of importance to the field of apicomplexan biology.

Thank you to the referee for their careful reading of our manuscript. We agree with the reviewers suggestion in regards to the supplementary videos of *P. falciparum* rupture, and have cropped the dimensions and shortened the lengths of all four videos to focus on the key egress events.

Referee #2:

The authors did an excellent job addressing the comments of the reviewers. The added data, model figures and edits to the manuscript make the document more complete and impactful.

We appreciate the reviewer for their review, and thank them for all of their suggestions, which have improved the quality and impact of this manuscript.

Referee #3:

The authors have provided additional data to support their findings. Some limitations that I previously highlighted remain:

Novelty: There is no new data that supports a drastic increase in novelty. As previously stated, the impact of this paper is taken away by previous work by the same group and others. The identity of CLIP is novel and the fact that the complex is made of at least 3 components is also. This is the third protein complex that has been identified to be required for rhoptry release. The authors do provide data to suggest that it might be the Proline Rich Domain (PRD), of CLAMP might be important for signaling (but not complex formation) into the cell upon complex engagement. This, as they state, could be analogous to how claudin transduce signal through the membrane is not required for complex formation but it needed for rhoptry release. This does give additional insight on mechanism. I see that novelty has not been brought up by the other referees, so perhaps my views do not represent the greater community.

P. falciparum data: More data has been provided that make it more likely that the phenotype is what they think it is, without directly showing this. No definitive results can be deduced from one video, containing one egressing schizont, in each condition. This is not at standard for the field. There are widely available protocols to look at Pf egress and invasion quantitatively (eg see Blackmans work). It remains possible, yet unlikely given the work in Toxo, that loss of SPATR and CLAMP cause defects in parasite replication.

Thank you to the reviewer for again carefully reviewing our manuscript. To attempt to better address these concerns about the role of the complex in *P. falciparum*, we have fully quantified the prevalence of schizonts and rings at 36 and 48 hours post invasion in synchronized parasite culture, and have included this data in Figure 6H-I. This data demonstrates that loss of both *Pf*CLAMP and *Pf*SPATR does not significantly affect the prevalence of schizonts at 36 h or 48 h, providing strong evidence that the CLAMP complex is not required for egress or replication in *P. falciparum.* In contrast, when either *Pf*CLAMP or *Pf*SPATR are knocked down there is a significant difference in the number of ring-stage parasites only at 48 h, demonstrating the CLAMP complex's role in invasion.

We feel this new data, along with our previous experiments showing monitoring growth and egress (Fig 6F-G, videos EV3-6), demonstrates convincingly that *Pf*CLAMP and *Pf*SPATR knockdown parasites replicate and egress normally, but fail to invade and form new-ring stage parasites – phenocopying our extensive work on the *T. gondii* CLAMP complex. Though we have not quantified invasion events directly for *Plasmodium* – which would require setting up this time consuming assay in our lab – this data further resolves the precise stage at which the *P. falciparum* parasites are impacted by the loss of the CLAMP complex, without delaying publication of the manuscript.

Dear Sebastian,

Congratulations on an excellent manuscript, I am pleased to inform you that your manuscript has been accepted for publication in the EMBO Journal. Thank you for your comprehensive response to the referee concerns and for providing detailed source data. It has been a pleasure to work with you to get this to the acceptance stage.

I will begin the final checks on your manuscript before submitting to the publisher next week. Once at the publisher, it will take about 3 weeks for you manuscript to be published online. As a reminder, the entire review process, including referee concerns and your point-by-point response, will be available to readers.

I will be in touch throughout the final editorial process until publication. In the meantime, I hope you find time to celebrate!

Kind regards,

Kelly

Kelly M Anderson, PhD
Editor, The EMBO Journal
k.anderson@embojournal.org

Your manuscript will be processed for publication in the journal by EMBO Press. Manuscripts in the PDF and electronic editions of The EMBO Journal will be copy edited, and you will be provided with page proofs prior to publication. Please note that supplementary information is not included in the proofs.

You will be contacted by Wiley Author Services to complete licensing and payment information. The required 'Page Charges Authorization Form' is available here: https://www.embopress.org/pb-assets/embo-site/tej_apc.pdf - please download and complete the form and return to embopressproduction@wiley.com

EMBO Press participates in many Publish and Read agreements that allow authors to publish Open Access with reduced/no publication charges. Check your eligibility: https://authorservices.wiley.com/author-resources/Journal-Authors/open-access/affiliation-policies-payments/index.html

Should you be planning a Press Release on your article, please get in contact with embojournal@wiley.com as early as possible, in order to coordinate publication and release dates.

EMBO Press Author Checklist

Corresponding Author Name: Sebastian Lourido
Journal Submitted to: The EMBO Journal
Manuscript Number: EMBOJ-2022-113155

USEFUL LINKS FOR COMPLETING THIS FORM
The EMBO Journal - Author Guidelines
EMBO Reports - Author Guidelines
Molecular Systems Biology - Author Guidelines
EMBO Molecular Medicine - Author Guidelines

Reporting Checklist for Life Science Articles (updated January
This checklist is adapted from Materials Design Analysis Reporting (MDAR) Checklist for Authors. MDAR establishes a minimum set of requirements in transparent reporting in the life sciences (see Statement of Task: 10.31222/osf.io/9sm4x). Please follow the journal's guidelines in preparing your
Please note that a copy of this checklist will be published alongside your article.

Abridged guidelines for figures
1. Data
The data shown in figures should satisfy the following conditions:
- the data were obtained and processed according to the field's best practice and are presented to reflect the results of the experiments in an accurate and unbiased manner.
- ideally, figure panels should include only measurements that are directly comparable to each other and obtained with the same assay.
- plots include clearly labeled error bars for independent experiments and sample sizes. Unless justified, error bars should not be shown for technical
- if n<5, the individual data points from each experiment should be plotted. Any statistical test employed should be justified.
- Source Data should be included to report the data underlying figures according to the guidelines set out in the authorship guidelines on Data

2. Captions
Each figure caption should contain the following information, for each panel where they are relevant:
- a specification of the experimental system investigated (eg cell line, species name).
- the assay(s) and method(s) used to carry out the reported observations and measurements.
- an explicit mention of the biological and chemical entity(ies) that are being measured.
- an explicit mention of the biological and chemical entity(ies) that are altered/varied/perturbed in a controlled manner.
- the exact sample size (n) for each experimental group/condition, given as a number, not a range;
- a description of the sample collection allowing the reader to understand whether the samples represent technical or biological replicates (including how many animals, litters, cultures, etc.).
- a statement of how many times the experiment shown was independently replicated in the laboratory.
- definitions of statistical methods and measures:
  - common tests, such as t-test (please specify whether paired vs. unpaired), simple χ2 tests, Wilcoxon and Mann-Whitney tests, can be unambiguously identified by name only, but more complex techniques should be described in the methods section;
  - are tests one-sided or two-sided?
  - are there adjustments for multiple comparisons?
  - exact statistical test results, e.g., P values = x but not P values < x;
  - definition of 'center values' as median or average;
  - definition of error bars as s.d. or s.e.m.

Please complete ALL of the questions below.
Select "Not Applicable" only when the requested information is not relevant for your study.

Materials

Newly Created Materials	Information included in the manuscript?	In which section is the information available? (Reagents and Tools Table, Materials and Methods, Figures, Data Availability Section)
New materials and reagents need to be available; do any restrictions apply?	Yes	No restrictions. Materials and Methods

Antibodies	Information included in the manuscript?	In which section is the information available? (Reagents and Tools Table, Materials and Methods, Figures, Data Availability Section)
For **antibodies** provide the following information:<br>- Commercial antibodies: RRID (if possible) or supplier name, catalogue number and or/clone number<br>- Non-commercial: RRID or citation	Yes	Materials and Methods

DNA and RNA sequences	Information included in the manuscript?	In which section is the information available? (Reagents and Tools Table, Materials and Methods, Figures, Data Availability Section)
**Short novel DNA or RNA including primers, probes**: provide the sequences.	Yes	Supplemental Table 1, Materials and Methods.

Cell materials	Information included in the manuscript?	In which section is the information available? (Reagents and Tools Table, Materials and Methods, Figures, Data Availability Section)
**Cell lines:** Provide species information, strain. Provide accession number in repository **OR** supplier name, catalog number, clone number, and/**OR** RRID.	Yes	Materials and Methods
**Primary cultures:** Provide species, strain, sex of origin, genetic modification status.	Not Applicable	
Report if the cell lines were recently **authenticated** (e.g., by STR profiling) and tested for mycoplasma contamination.	Not Applicable	

Experimental animals	Information included in the manuscript?	In which section is the information available? (Reagents and Tools Table, Materials and Methods, Figures, Data Availability Section)
**Laboratory animals or Model organisms:** Provide species, strain, sex, age, genetic modification status. Provide accession number in repository **OR** supplier name, catalog number, clone number, **OR** RRID.	Not Applicable	
**Animal observed in or captured from the field:** Provide species, sex, and age where possible.	Not Applicable	
Please detail **housing and husbandry conditions**.	Not Applicable	

Plants and microbes	Information included in the manuscript?	In which section is the information available? (Reagents and Tools Table, Materials and Methods, Figures, Data Availability Section)
**Plants:** provide species and strain, ecotype and cultivar where relevant, unique accession number if available, and source (including location for collected wild specimens).	Not Applicable	
**Microbes:** provide species and strain, unique accession number if available, and source.	Yes	Materials and Methods

Human research participants	Information included in the manuscript?	In which section is the information available? (Reagents and Tools Table, Materials and Methods, Figures, Data Availability Section)
If collected and within the bounds of privacy constraints report on age, sex and gender or ethnicity for all study participants.	Not Applicable	

Core facilities	Information included in the manuscript?	In which section is the information available? (Reagents and Tools Table, Materials and Methods, Figures, Data Availability Section)
If your work benefited from core facilities, was their service mentioned in the acknowledgments section?	Yes	ACKNOWLEDGEMENTS

Design

Study protocol	Information included in the manuscript?	In which section is the information available? (Reagents and Tools Table, Materials and Methods, Figures, Data Availability Section)
If study protocol has been **pre-registered, provide DOI in the manuscript**. For clinical trials, provide the trial registration number **OR** cite DOI.	Not Applicable	
Report the **clinical trial registration number** (at ClinicalTrials.gov or equivalent), where applicable.	Not Applicable	

Laboratory protocol	Information included in the manuscript?	In which section is the information available? (Reagents and Tools Table, Materials and Methods, Figures, Data Availability Section)
Provide DOI OR other citation details if **external detailed step-by-step protocols** are available.	Not Applicable	

Experimental study design and statistics	Information included in the manuscript?	In which section is the information available? (Reagents and Tools Table, Materials and Methods, Figures, Data Availability Section)
Include a statement about **sample size** estimate even if no statistical methods were used.	Not Applicable	
Were any steps taken to minimize the effects of subjective bias when allocating animals/samples to treatment (e.g. **randomization procedure**)? If yes, have they been described?	Not Applicable	
Include a statement about **blinding** even if no blinding was done.	Not Applicable	
Describe **inclusion/exclusion criteria** if samples or animals were excluded from the analysis. Were the criteria pre-established?<br><br>If sample or data points were omitted from analysis, report if this was due to attrition or intentional exclusion and provide justification.	Not Applicable	
For every figure, are **statistical tests** justified as appropriate? Do the data meet the assumptions of the tests (e.g., normal distribution)? Describe any methods used to assess it. Is there an estimate of variation within each group of data? Is the variance similar between the groups that are being statistically compared?	Yes	Materials and Methods, Figures

Sample definition and in-laboratory replication	Information included in the manuscript?	In which section is the information available? (Reagents and Tools Table, Materials and Methods, Figures, Data Availability Section)
In the figure legends: state number of times the experiment was **replicated** in laboratory.	Not Applicable	
In the figure legends: define whether data describe **technical or biological replicates**.	Yes	Materials and Methods, Figures.

Ethics

Ethics	Information included in the manuscript?	In which section is the information available? (Reagents and Tools Table, Materials and Methods, Figures, Data Availability Section)
Studies involving **human participants**: State details of **authority granting ethics approval** (IRB or equivalent committee(s), provide reference number for approval.	Not Applicable	
Studies involving **human participants**: Include a statement confirming that **informed consent** was obtained from all subjects and that the experiments conformed to the principles set out in the WMA Declaration of Helsinki and the Department of Health and Human Services Belmont Report.	Not Applicable	
Studies involving **human participants: For** publication of **patient photos**, include a statement confirming that consent to publish was obtained.	Not Applicable	
Studies involving experimental **animals**: State details of **authority granting ethics approval** (IRB or equivalent committee(s), provide reference number for approval. Include a statement of compliance with ethical regulations.	Not Applicable	
Studies involving **specimen and field samples:** State if relevant **permits** obtained, provide details of authority approving study; if none were required, explain why.	Not Applicable	

Dual Use Research of Concern (DURC)	Information included in the manuscript?	In which section is the information available? (Reagents and Tools Table, Materials and Methods, Figures, Data Availability Section)
Could your study fall under dual use research restrictions? Please check biosecurity documents and list of **select agents and toxins** (CDC): https://www.selectagents.gov/sat/list.htm	Not Applicable	
If you used a select agent, is the security level of the lab appropriate and reported in the manuscript?	Not Applicable	
If a study is subject to dual use research of concern regulations, is the name of the **authority granting approval and reference number** for the regulatory approval provided in the manuscript?	Not Applicable	

Reporting

The MDAR framework recommends adoption of discipline-specific guidelines, established and endorsed through community initiatives. Journals have their own policy about requiring specific guidelines and recommendations to complement MDAR.

Adherence to community standards	Information included in the manuscript?	In which section is the information available? (Reagents and Tools Table, Materials and Methods, Figures, Data Availability Section)
State if relevant guidelines or checklists (e.g., **ICMJE, MIBBI, ARRIVE, PRISMA**) have been followed or provided.	Not Applicable	
For **tumor marker prognostic studies,** we recommend that you follow the **REMARK** reporting guidelines (see link list at top right). See author guidelines, under 'Reporting Guidelines'. Please confirm you have followed these guidelines.	Not Applicable	
For **phase II and III randomized controlled trials**, please refer to the **CONSORT** flow diagram (see link list at top right) and submit the CONSORT checklist (see link list at top right) with your submission. See author guidelines, under 'Reporting Guidelines'. Please confirm you have submitted this list.	Not Applicable	

Data Availability

Data availability	Information included in the manuscript?	In which section is the information available? (Reagents and Tools Table, Materials and Methods, Figures, Data Availability Section)
Have **primary datasets** been deposited according to the journal's guidelines (see 'Data Deposition' section) and the respective accession numbers provided in the Data Availability Section?	Yes	Data Availability
Were **human clinical and genomic datasets** deposited in a public access-controlled repository in accordance to ethical obligations to the patients and to the applicable consent agreement?	Not Applicable	
Are **computational models** that are central and integral to a study available without restrictions in a machine-readable form? Were the relevant accession numbers or links provided?	Not Applicable	
If publicly available data were reused, provide the respective **data citations in the reference list**.	Not Applicable	
