# Supplementary material for: A conserved complex of microneme proteins mediates rhoptry discharge in Toxoplasma
Source: EMBO J. 2023 Oct 27;42(23):e113155. doi: 10.15252/embj.2022113155 (PMC10690463; doi:10.15252/embj.2022113155)
Supplement: Supplementary file 12 — Source Data for Expanded View [file EMBJ-42-e113155-s009.zip › EMBOJ-2022-113155R1-Figure_EV4_Source_Data-sd.zip!/Figure EV4/EV4D.pdf]

Figure EV4D

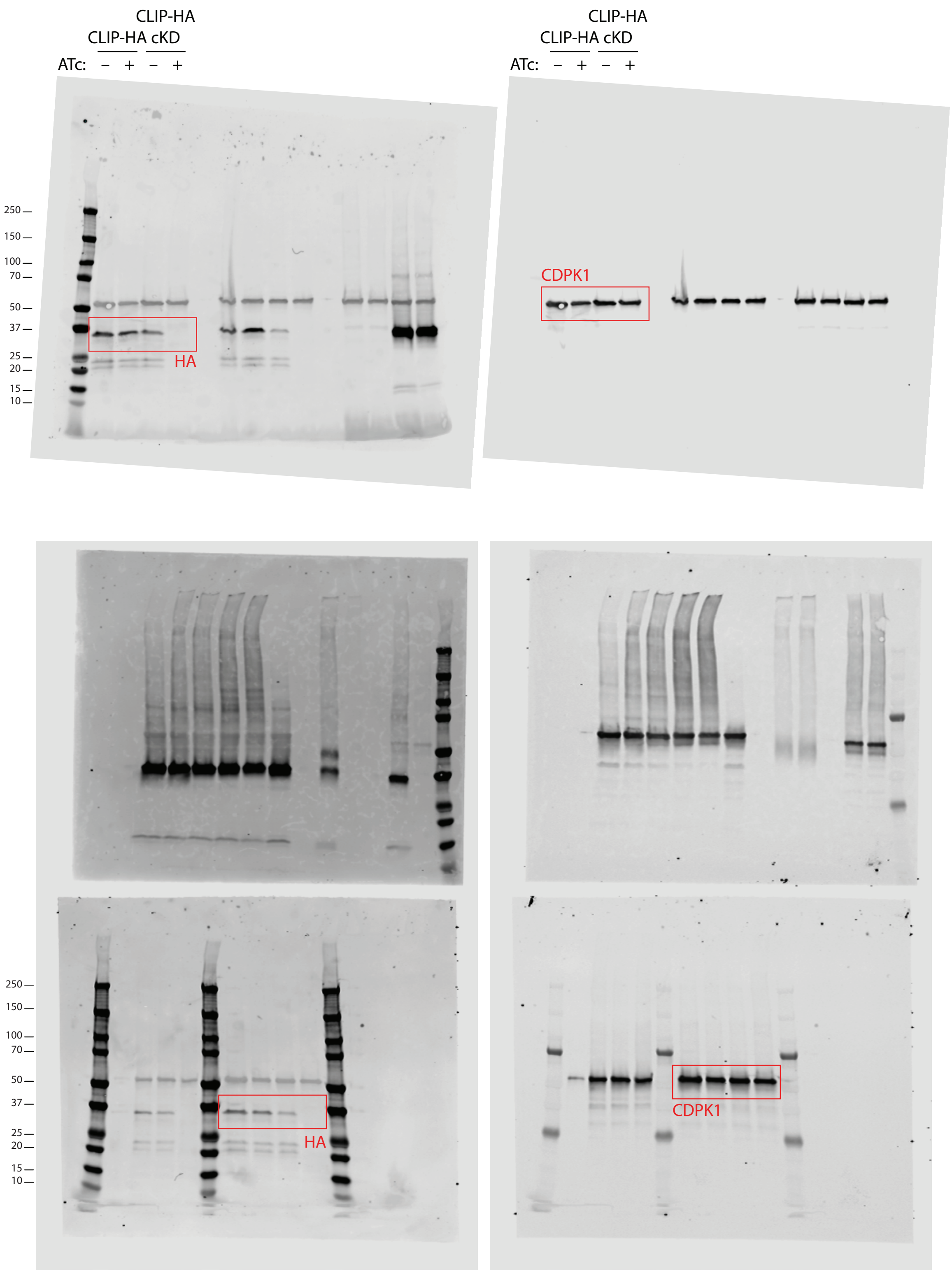
CLIP-HA
CLIP-HA cKD
ATc: – + – +
250
150
100
70
50
37
25
20
15
10
HA
CLIP-HA
CLIP-HA cKD
ATc: – + – +
CDPK1
250
150
100
70
50
37
25
20
15
10
HA
CDPK1
